# Dipyrrole-Fused Naphthalene Diimide as a Building Block for High-Performance Organic Semiconductors

**DOI:** 10.1021/jacs.6c07621

**Published:** 2026-07-17

**Authors:** Kexiang Zhao, Waner He, Xingyu Yang, Yuko Kishida, Hidehiro Uekusa, Zichen Yang, Hidetoshi Matsumoto, Keigo Nonaka, Yoshimitsu Sagara, Juntaro Nogami, Ken Tanaka, Tian-Yu Zhang, Ze-Fan Yao, Tsuyoshi Michinobu

**Affiliations:** † Department of Materials Science and Engineering, 13290 Institute of Science Tokyo, 2-12-1 Ookayama, Meguro-ku, Tokyo 152-8552, Japan; ‡ Department of Chemistry, School of Science, 13290 Institute of Science Tokyo, Tokyo 152-8551, Japan; § College of Chemistry and Molecular Engineering, Peking University, Beijing 100871, China

## Abstract

Core annulation of naphthalene diimide (NDI) represents a powerful strategy to modulate its electronic structure and solid-state organization. Here, we report the first pyrrole-fused NDI (NDPI) framework and elucidate its structural and electronic characteristics. Single-crystal X-ray diffraction reveals a highly planar π-core with short π–π stacking distance (3.33 Å) and strong intermolecular N–H···O hydrogen bonding (2.07 Å), highlighting the unique supramolecular features introduced by pyrrole annulation. Building on this motif, a series of donor–acceptor polymers was synthesized and systematically evaluated in OFET devices. The polymers exhibit optical bandgaps of 1.32–1.39 eV. Device mobilities range from 0.10 to 0.59 cm^2^ V^–1^ s^–1^, with charge transport behavior tunable from balanced ambipolar to unipolar n-type depending on donor strength. Correlation of AFM and GIWAXS analyses suggests that charge mobility is governed not solely by crystallinity, but by the interplay between π–π stacking and domain connectivity. This work establishes NDPI as a versatile building block for high-performance organic semiconductors.

## Graphical abstract

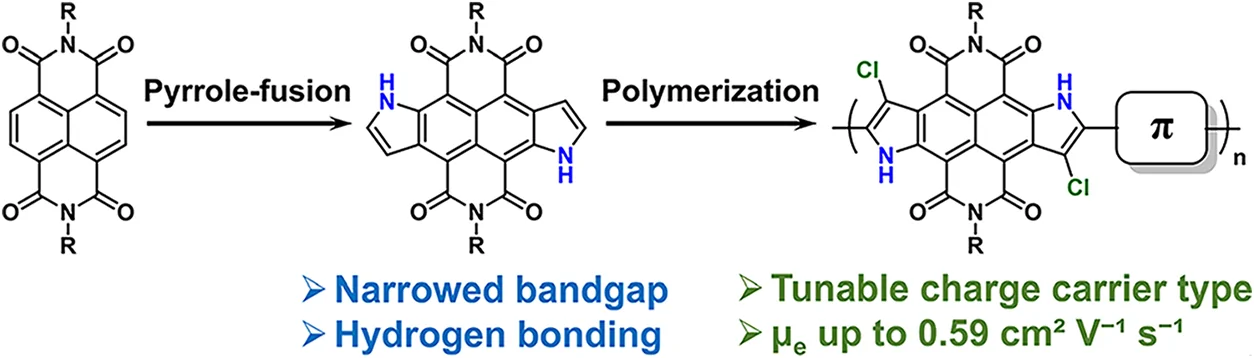

## Introduction

Naphthalene diimides (NDIs) are among the most extensively studied electron-deficient π-conjugated frameworks for organic electronic applications.^1^ Their rigid and planar aromatic core,^2^ high electron affinity,^3^ excellent thermal and chemical stability,^4^ and favorable solid-state organization^5^ have established NDIs as benchmark n-type semiconductors in organic field-effect transistors (OFETs),^6^ organic photovoltaics,^7^ and related optoelectronic devices.^8^ A key advantage of NDIs lies in their structural versatility, which enables systematic tuning of optoelectronic properties through molecular engineering at the imide positions or the conjugated core.^9^

Core annulation has emerged as a particularly powerful strategy for modulating the electronic structure and charge-transport behavior of NDIs.^10^ Fusion of additional aromatic or heteroaromatic rings directly onto the NDI core extends π-conjugation and enforces molecular planarity,^11^ often resulting in narrowed optical band gaps,^12^ enhanced intermolecular interactions,^13^ and improved charge-carrier mobilities.^14^ Accordingly, core-annulated NDIs have become an important platform for the development of high-performance organic semiconductors.

Within this context, annulation with five-membered heteroaromatic rings has attracted considerable attention. The smaller ring size allows efficient fusion to the NDI bay region while preserving planarity and minimizing steric congestion,^15^ whereas the presence of heteroatoms provides a powerful handle for tuning optoelectronic properties.^16^ The broad palette of available five-membered heteroaromatics, such as thiophene, furan, and pyrrole, offers exceptional flexibility for tailoring donor–acceptor interactions, intermolecular packing, and charge-transport pathways, advantages that are less accessible with more electronically uniform six-membered aromatic rings.^17^ Among these heteroaromatic motifs, pyrrole represents a particularly compelling yet unexplored candidate for NDI core annulation. Compared to its sulfur- and oxygen-containing analogs,^18^ pyrrole exhibits a stronger electron-donating character capable of reinforcing intramolecular donor–acceptor interactions with the electron-deficient NDI core (Figure 1). In addition, the pyrrolic nitrogen offers opportunities for enhanced intermolecular interactions through hydrogen bonding, which can be exploited in supramolecular assembly.^19^ Moreover, pyrrole is more reactive toward the electrophiles, which enables easier and versatile post functionalization. These attributes suggest that pyrrole fusion could endow NDIs with properties distinct from, and potentially superior to, those of previously reported five-membered heteroaromatic ring annulated NDIs.

**Figure 1:**
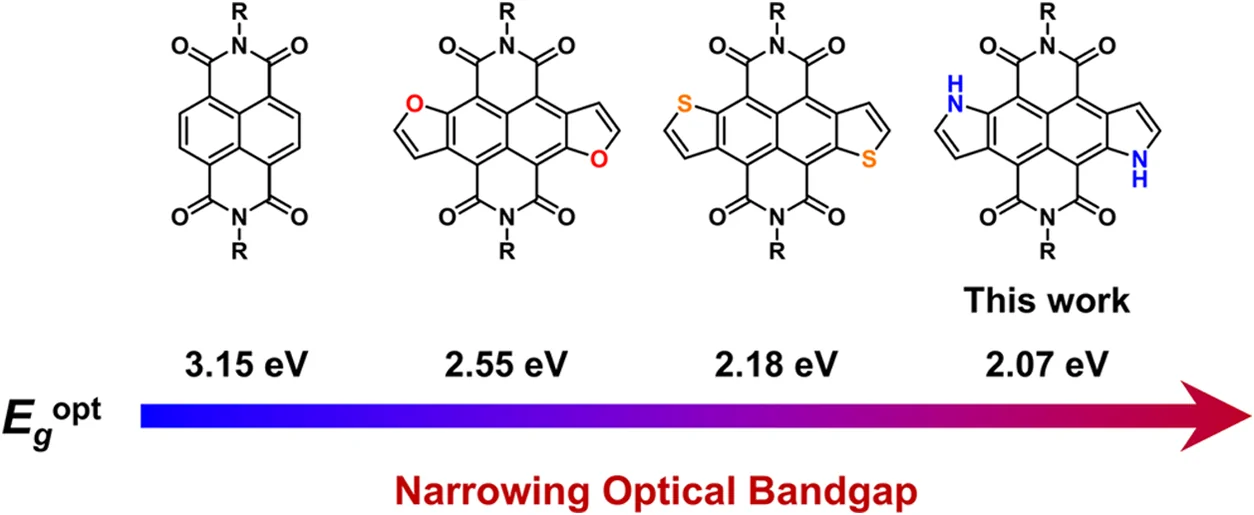
Fusion of pyrrole segments to the NDI core narrows the optical bandgap compared with those of previously reported five-membered aromatic heterocycle fused NDIs.

Despite these anticipated advantages, pyrrole-fused NDI derivatives have remained conspicuously absent from the literature. This omission can likely be attributed to intrinsic synthetic challenges in lateral construction of the pyrrole ring onto the NDI core using conventional methods, such as the Fisher indole synthesis.^20^ As a result, the influence of pyrrole annulation on the structure–property relationships of NDIs have not been previously elucidated. Herein, we report the successful synthesis of pyrrole-fused NDI (**NDPI**) and provide unambiguous structural insight through single-crystal X-ray diffraction analysis. Building on this newly accessible NDI motif, a series of donor–acceptor (D–A) polymers incorporating this unit were synthesized and systematically evaluated in OFET devices. These materials exhibit either ambipolar or unipolar n-type charge transport, with electron mobilities reaching up to 0.59 cm^2^ V^–1^ s^–1^.

## Results and Discussion

The fusion of a pyrrole ring onto the NDI core is conceptually analogous to the synthesis of indole from phenyl derivatives. In particular, the classical Fischer indole synthesis relies on hydrazone intermediates followed by a [3,3]-sigmatropic rearrangement.^21^ However, NDI-based hydrazones have not been reported, and more importantly, the strongly electron-withdrawing nature of the NDI core would be expected to disfavor the key rearrangement step. We therefore pursued an alternative annulation strategy based on the cyclization of 2-alkynylaniline derivatives.^22^ In this approach, the amino group is electronically deactivated while the alkynyl moiety is activated toward intramolecular cyclization, thereby effectively compensating for the electron deficiency of the NDI framework and enabling pyrrole fusion.

The synthesis of **NDPI** begins with diamino NDI **1** which has been synthesized by the SNAr reaction of dibromo NDI with allylamine followed by subsequent Pd-catalyzed deallylation (Scheme 1).^23^ However, this could not be reproduced when alkyl side chains were introduced, likely due to poor solubility of the intermediates. To address this limitation, allylamine was replaced with *p*-methoxybenzylamine (PMB amine), a commonly used amine-protecting group that can be readily cleaved under acidic conditions. Treatment with trifluoroacetic acid (TFA) afforded diamino NDI **1** in good to excellent yield. Bromination of **1** in dichloromethane with excess bromine then produced compound **2** in very good yield. Stille coupling of **2** with trimethylsilyl (TMS) acetylene stannane, followed by ZnI_2_-catalyzed cyclization, furnished the TMS-substituted **NDPIs**. Subsequent removal of the TMS group in THF using TBAF yielded the corresponding unsubstituted **NDPI** derivatives.

**Scheme 1:**
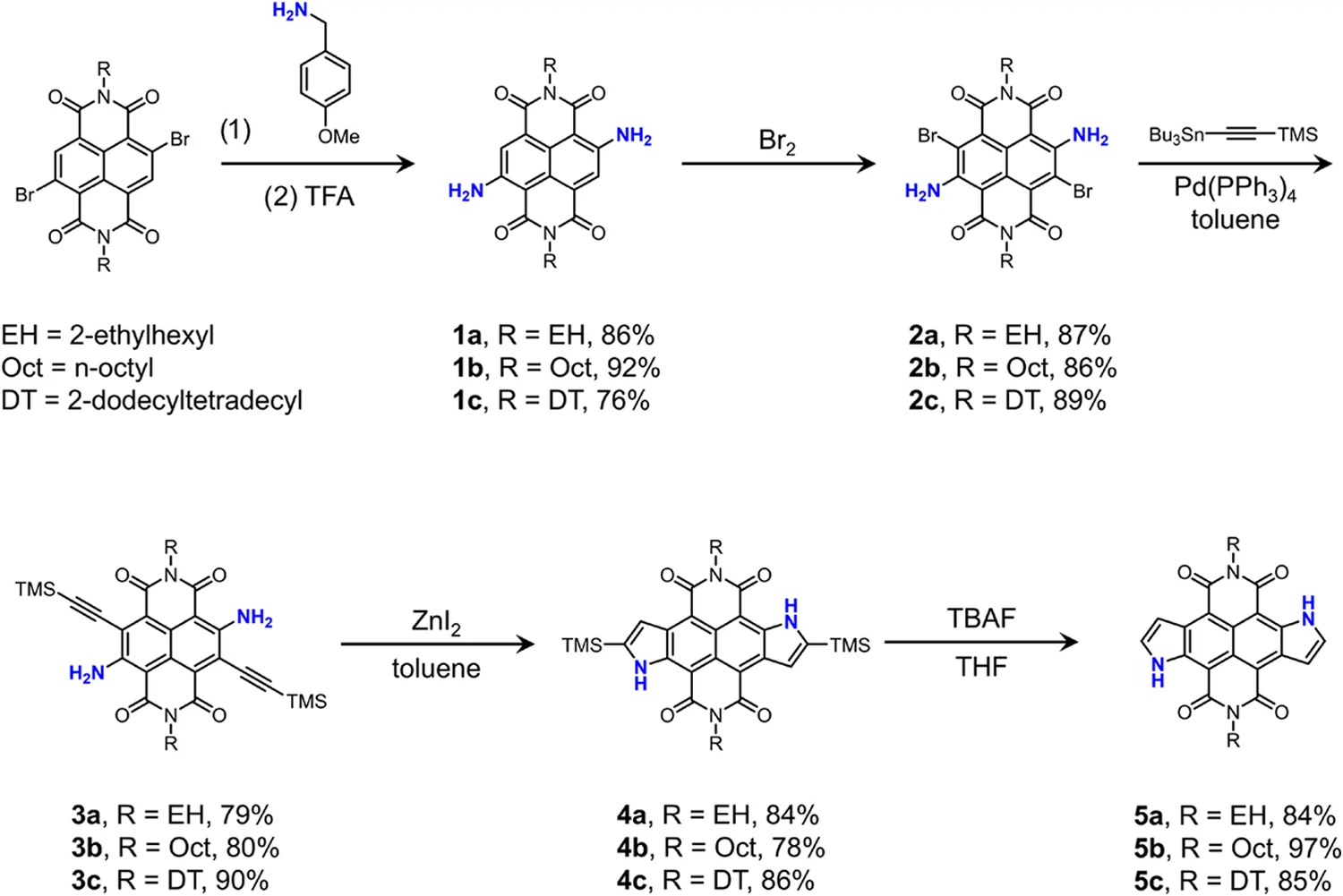
Synthetic Route toward the NDPI

Single crystals of **5b** suitable for X-ray diffraction were obtained by recrystallization from nitrobenzene (Figure 2). Compound **5b** adopts a columnar packing structure along the *b* axis. These columns are interconnected along the *c* axis through intermolecular hydrogen bonding between the pyrrolic NH and the carbonyl oxygen of adjacent columns, while molecules within neighboring columns are oriented nearly perpendicular to one another. The short intermolecular N–H···O distance of 2.07 Å indicates strong hydrogen bonding. In addition to this intermolecular interaction, the pyrrolic NH also forms an intramolecular hydrogen bond with a neighboring carbonyl group, with an N–H···O distance of 2.27 Å. Such dual hydrogen-bonding interactions may be further exploited in the construction of supramolecular architectures. Structurally, the molecular backbone of **5b** is highly planar. The calculated 2D-ICSS^24^ and ACID^25^ plots of **NDPI** (Figure S9) indicate the strong aromaticity of pyrrole-fused NDI core. Within each column, the π–π stacking distance is 3.33 Å, indicative of strong intermolecular electronic coupling.

**Figure 2:**
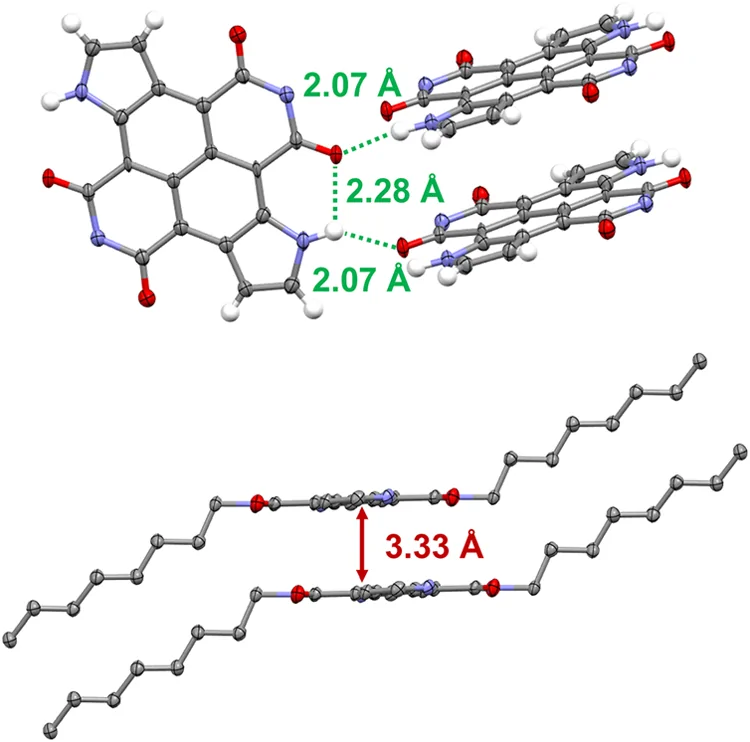
Crystal structure of **5b**. Thermal ellipsoids are drawn at the 50% probability level. The octyl chains of the top packing motif are omitted for clarity. H atoms are omitted for clarity except for those with intermolecular interactions.

The optical properties of **NDPI** further reflect the electronic consequences of pyrrole annulation (Figure S3). As shown in Figure 3, the absorption spectra of **5a**, NDI-EH, and NDTI-EH reveal substantial bathochromic shifts upon core annulation. Compared with pristine NDI, **5a** exhibits a redshift exceeding 200 nm, consistent with extended conjugation. The magnitude of this shift depends on both the size of the fused aromatic segment and the strength of intramolecular charge transfer (ICT). Among five-membered heteroaromatic rings, pyrrole is generally regarded as the strongest electron donor. Accordingly, **5a** displays more pronounced red-shifted absorption than NDTI due to enhanced donor–acceptor interaction. The photoluminescence quantum yield of **5a** is 0.31 with a lifetime of 7.4 ns. This quantum yield is lower than that of NDTI (Φ = 0.76), which can be attributed to stronger ICT character facilitating nonradiative decay. Cyclic voltammetry measurements (Figure S1) estimate *E*_HOMO_ and *E*_LUMO_ values of −5.55 and −3.59 eV, respectively, for **5a**. These frontier orbital levels are shallower than those of NDTI and NDI, reflecting the stronger electron-donating nature of pyrrole. The electrochemical bandgap (*E*_g_) of **5a** is 1.96 eV, in good agreement with the optical bandgap (2.07 eV) estimated from the absorption onset at 600 nm. The calculated HOMO/LUMO distributions of **NDPI** (Figure S8) are very similar to those of NDTI due to the structural similarity.

**Figure 3:**
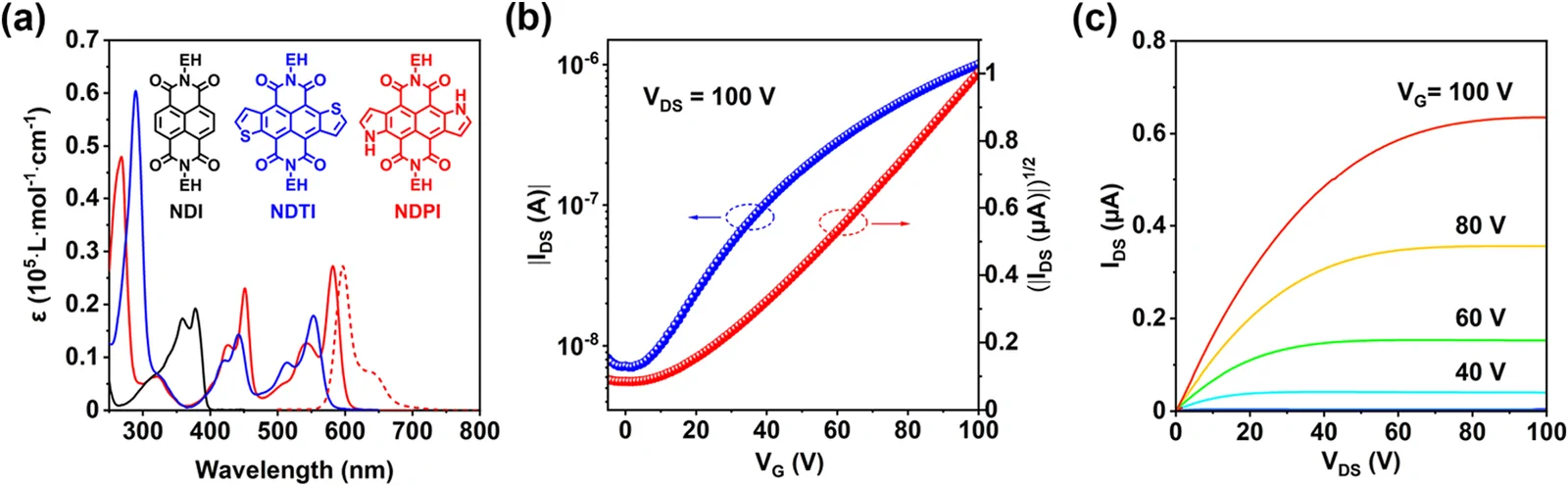
(a) Solid lines: absorption spectra of **5a**, NDTI-EH, and NDI-EH in chloroform (10^–5^ mol/L); dashed line: photoluminescence of **5a** in chloroform (10^–5^ mol/L) excited at 265 nm. (b) Transfer and (c) output characteristics of **5b** based OFET fabricated on ODTS-modified substrate. The channel length and width are 100 and 1000 μm, respectively. Electron mobilities were extracted from the saturation regime.

The **NDPIs** are bench-stable solids and exhibit no detectable degradation even after 18 months under ambient conditions (Figure S4). Moreover, thermogravimetric analysis shows good thermal stability of **NDPIs** with 5% weight-loss temperatures above 360 °C (Figure S6). Top-contact/bottom-gate (TC/BG) OFETs fabricated by vacuum deposition of **5b** onto ODTS-modified Si/SiO_2_ substrates followed by gold electrode deposition exhibit typical n-channel characteristics, with a maximum electron mobility of 0.029 cm^2^ V^–1^ s^–1^ and an average mobility of 0.024 cm^2^ V^–1^ s^–1^. The threshold voltage and on/off ratio for **5b** are 36.9 ± 1.8 V and (1.3 ± 0.4) × 10^2^, respectively. These values are comparable to those of the previously reported core-annulated NDI derivatives (Figure S12).^16a,16b,18a,26^ To gain insight into the influence of pyrrole fusion on charge transport, transfer integrals were calculated for representative **NDPI** dimers (Figure S10).^27^ The slipped cofacial dimer exhibits a moderate electron transfer integral of 44 meV. By comparison, the dimer between adjacent molecular columns affords a substantially larger transfer integral of 111 meV, suggesting that the intermolecular hydrogen-bonding network might strengthen electronic coupling and facilitate electron transport.

To employ **NDPI** as a versatile building block for conjugated polymers, selective functionalization of the pyrrole moiety is essential. In contrast to NDTI, where thiophene can be regioselectively brominated at the α-position, the pyrrole unit in **NDPI** is more susceptible to electrophilic substitution and exhibits distinct regioselectivity. Similar to indole, bromination preferentially occurs at the β-position. However, β-functionalization is unfavorable for polymerization because coupling at this position introduces significant steric hindrance between the carbonyl group and the comonomer. The resulting torsional disorder and large dihedral angles broaden the density of states and reduce intrachain mobility, effects that are particularly detrimental for n-type charge transport.^28^ Therefore, access to α-brominated **NDPI** is critical for high-performance polymers.

Due to the elevated HOMO energy level of **NDPI**, electrophilic bromination does not terminate at the β-position but proceeds further to the α-position under excess bromine, ultimately yielding tetrabrominated products unsuitable for linear polymer synthesis. To control regioselectivity, a chloride substituent was first introduced to block the β-position (Scheme 2). The subsequent Si–Br exchange afforded the desired α-brominated **NDPI** in good yield. The resulting dibromo monomer was polymerized with stannylated donor units via Stille coupling to generate donor–acceptor polymers **P-2T**, **P-TVT**, and **P-2F2T**. After polymerization, Soxhlet extraction using acetone, hexane, and dichloromethane removed oligomers and residual impurities. The final chloroform or chlorobenzene fractions were collected and reprecipitated into methanol to yield purified polymers suitable for characterization and device fabrication. The number-average molecular weights (*M*_n_) and dispersities (*Đ*) were determined by high-temperature GPC at 150 °C using trichlorobenzene as eluent (Figure S7). **P-2T**, **P-TVT**, and **P-2F2T** exhibit *M*_n_ values of 14.8, 19.9, and 27.5 kDa, with *Đ* values of 1.7, 2.2, and 2.4, respectively (Table 1). Thermogravimetric analysis confirms good thermal stability, with 5% weight-loss temperatures around 380 °C (Figure S6).

**Scheme 2:**
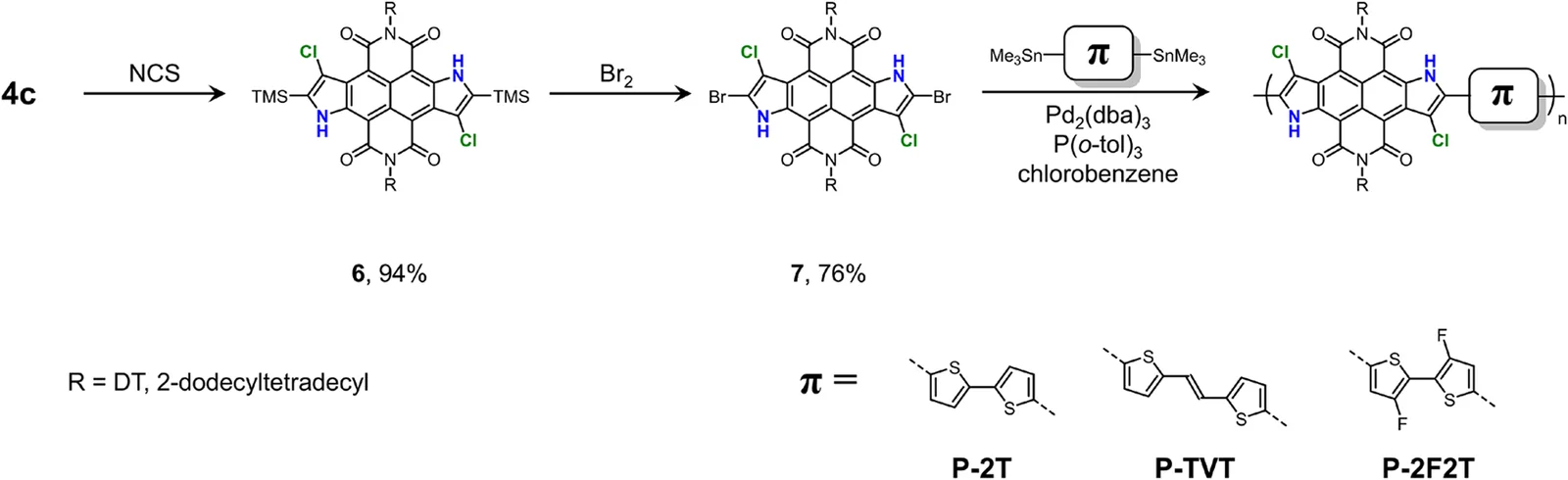
Synthetic Route toward the NDPI-Based Polymers

**Table 1:** Summary of Molecular Weights and Optoelectronic Properties of the NDPI-Based Polymers

polymer	*M*_n_ (kDa)^a^	*Đ*	λ_sol max,_ [nm]^b^	λ_film max,_ [nm]^c^	*E*_g_^opt^ [eV]^d^	*E*_HOMO_ [eV]^e^	*E*_LUMO_ [eV]^f^
**P-2T**	14.8	1.7	848	843	1.37	–5.4	–4.0
**P-TVT**	19.9	2.2	791	786	1.32	–5.3	–4.0
**P-2F2T**	27.5	2.4	829	829	1.39	–5.6	–4.2

a Molecular weight was measured by high-temperature GPC at 150 °C using trichlorobenzene as eluent.

b Absorption spectra were recorded in chlorobenzene solution.

c Polymer thin films were spin-coated onto the quartz substrates.

d *E*_g_^opt^ was calculated from the intercept of the absorption spectra (thin films). *E*_g_^opt^ = 1240 nm/λ_intercept_.

e HOMO levels were measured by photoemission yield spectroscopy in air (PYSA).

f *E*_LUMO_ = *E*_HOMO_+ *E*_g_^opt^.

DFT calculations on optimized trimeric models at the B3LYP-D3­(BJ)/def2-TZVP level (Figure 4d) reveal that steric repulsion between chloride substituents and adjacent carbonyl groups induces backbone twisting, with dihedral angles ranging from 13.5° to 14.1°. The maximum displacement of carbonyl oxygen atoms from the mean **NDPI** plane is approximately 0.74–0.76 Å. In contrast, the dihedral angles between **NDPI** and donor units are smaller than 5°, indicating nearly coplanar donor–acceptor junctions and effective conjugation. For the donor units, both difluorobithiophene (2F2T) and thiophene-vinylene-thiophene (TVT) show highly planar structure, whereas bithiophene (2T) shows slightly larger dihedral angle of 6.9°.

**Figure 4:**
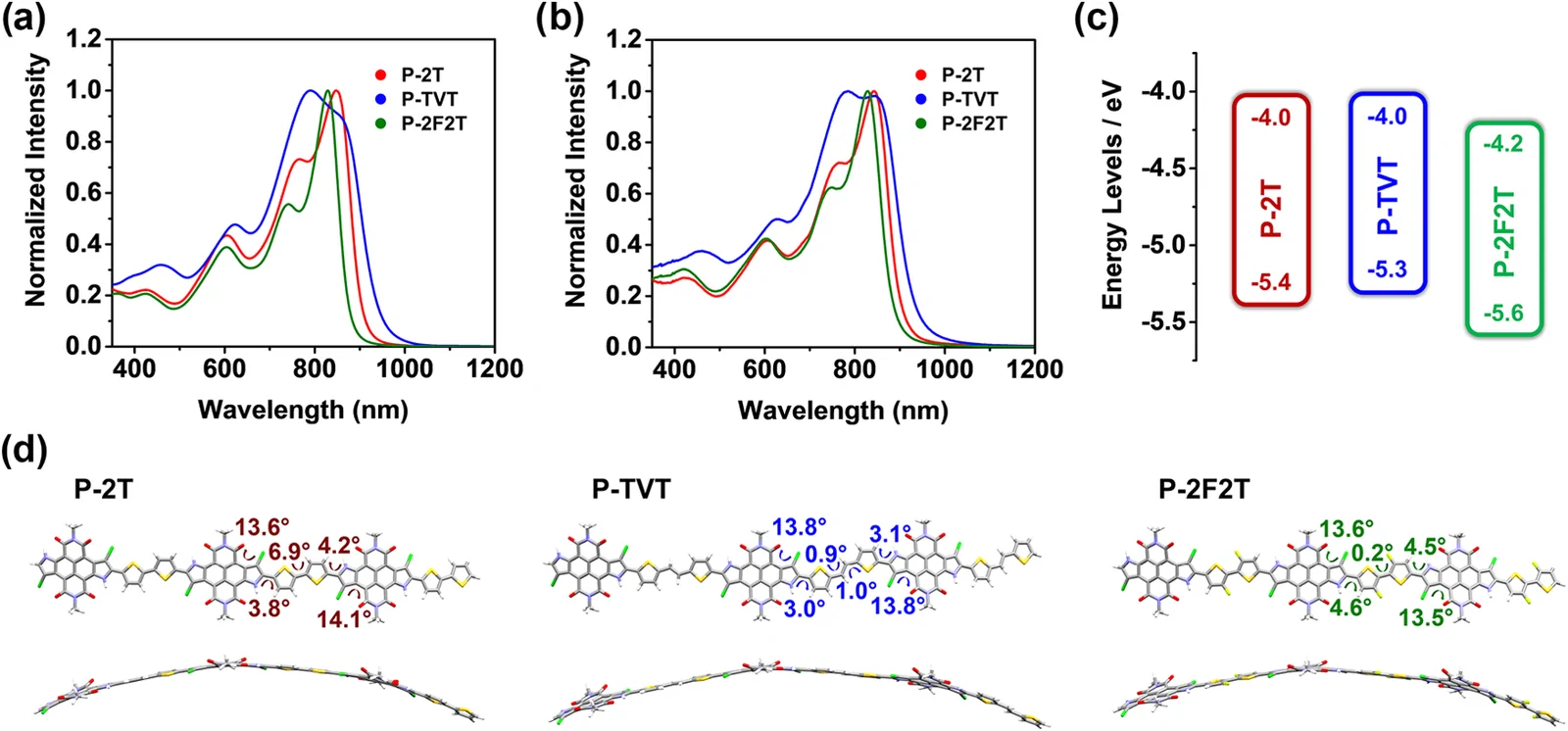
Normalized UV–vis absorption of **NDPI** based D–A polymers **P-2T**, **P-TVT** and **P-2F2T** measured (a) in chlorobenzene solutions and (b) as thin films. (c) Energy level diagrams and (d) DFT optimized structures of model oligomers.

The absorption spectra of the **NDPI**-based polymers (Figure 4a) display broad bands spanning 500–900 nm, with absorption tails extending to 1000 nm. Notably, thin-film spectra show no appreciable redshift relative to chlorobenzene solutions, suggesting weak aggregation due to backbone twisting. Consistently, variable-temperature absorption spectra of **NDPI**-based polymers (Figure S5) show negligible changes upon heating. Although the **NDPI** monomer exhibits stronger ICT and more red-shifted absorption than NDTI, the optical bandgaps (*E*_g_^opt^) of the polymers are 1.37 eV (**P-2T**), 1.32 eV (**P-TVT**), and 1.39 eV (**P-2F2T**). These values are slightly larger than that of the NDTI-based polymer with bithiophene (1.2 eV),^18^ consistent with reduced effective conjugation in the twisted **NDPI** backbone. Photoemission yield spectroscopy indicates the HOMO levels near −5.5 eV, and the LUMO levels, estimated from the HOMO levels and *E*_g_^opt^, are around −4.0 eV, supporting n-type transport (Figure 4c).^29^ Both the calculated (Figure S11) and experimental (Table 1) HOMO levels of **NDPI**-based polymers decrease in the order of **P-TVT**, **P-2T**, **P-2F2T**, which corresponds to the decreasing donor strength of the TVT, 2T, and 2F2T.

OFETs are fabricated using the TC/BG device architecture to investigate the charge transport properties of the **NDPI**-based polymers. The annealing temperatures of spin-coated films are optimized. As the donor strength of the comonomers decreases (TVT > 2T > 2F2T), the frontier molecular orbital energy levels of the corresponding polymers descend and the charge transport behavior (Table 2) switches from ambipolar (**P-TVT** and **P-2T**) to unipolar n-type (**P-2F2T**). It can be deduced that when acceptor comonomers, such as 2,1,3-benzothiadiazole, are used, the corresponding **NDPI**-based acceptor-acceptor (A-A) type polymers would exhibit even better electron charge transport behavior. Both **P-2T** (Figure 5a–d) and **P-TVT** (Figure 5e–h) showcase balanced ambipolar charge carrier behavior with electron-to-hole mobility ratios of 1.29 and 1.30, respectively. **P-2F2T**, on the other hand, showcases unipolar n-type mobility. The self-assembling (SAM) layer in OFETs could have large impact on device performance.^30^ 12-Cyclohexyldodecyl phosphonic acid (CDPA) modified SiO_2_/AlO_*x*_ acts as an excellent dielectric layer in OFETs and improves the field effect mobility.^31^ As the SAM layer switched from ODTS (Figure 5i,j) to CDPA (Figure 5k,l), the electron transport behavior of **P-2F2T** was improved with higher mobility (0.59 cm^2^ V^–1^ s^–1^) and larger on/off current ratio (>10^4^).

**Figure 5:**
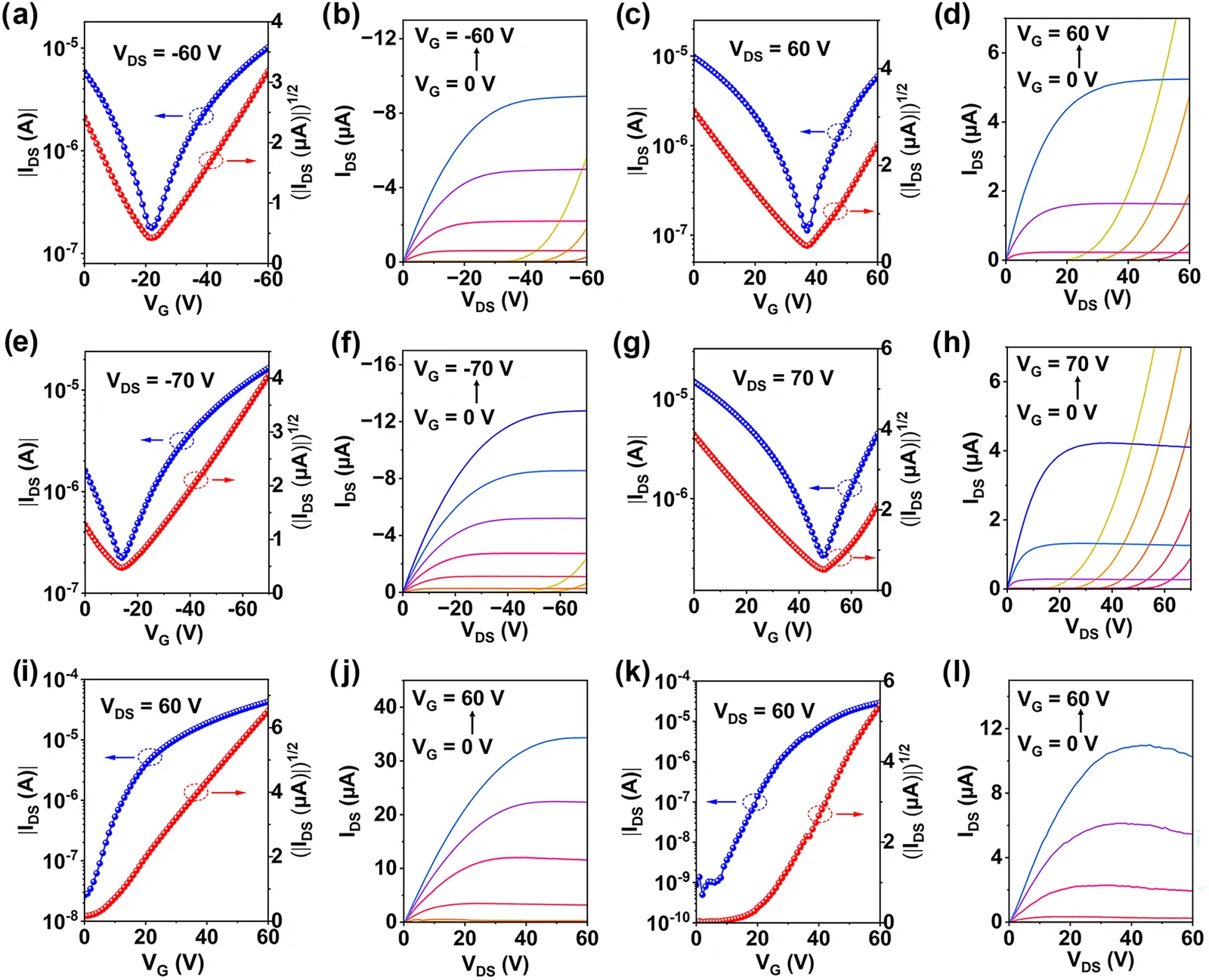
Transfer and output curves of OFETs based on **P-2T** (a–d), **P-TVT** (e–h), and **P-2F2T** (i–l). ODTS (a–j) or CDPA (k, l) was used as the SAM layer.

**Table 2:** Bottom-Gate/Top-Contact OFET Performance Parameters of the NDPI-Based Polymers

polymer	μ_h_ ^a^ (cm^2^ V^–1^ s^–1^)	*V*_th_/V	*I*_on_/*I*_off_	μ_e_ ^a^ (cm^2^ V^–1^ s^–1^)	*V*_th_/V	*I*_on_/*I*_off_
**P-2T**	0.11 (0.10)	–21.3 ± 1.3	(1.1 ± 0.4) × 10^2^	0.19 (0.13)	35.1 ± 1.6	(2.2 ± 0.8) × 10^2^
**P-TVT**	0.097 (0.085)	–4.9 ± 2.3	49 ± 11	0.18 (0.11)	48.8 ± 1.2	(1.1 ± 0.4) × 10^2^
**P-2F2T**				0.32 (0.25)	3.5 ± 2.2	(4.1 ± 1.6) × 10^3^
**P-2F2T** (CDPA)				0.59 (0.41)	22.0 ± 1.3	(4.9 ± 1.8) × 10^4^

a Maximum mobilities measured under vacuum, with average values (in parentheses) calculated from ten devices in the saturation regime.

To further rationalize the above results, we investigated the polymer film morphology and microstructure which were initially probed through atomic force microscopy (AFM) characterizations of the films prepared under the same conditions used for optimal device fabrication (Figure 6). AFM characterization reveals pronounced differences in thin-film morphology across the three polymers. Both **P-2T** and **P-TVT** form large crystalline domains with strong phase contrast, indicative of pronounced aggregation and domain segregation. The surface appears relatively coarse, suggesting the formation of extended crystalline grains with limited interdomain connectivity. In contrast, **P-2F2T** produces the smooth and homogeneous films on ODTS, and the film became even more smooth on CDPA, characterized by finer, more continuous fibrillar networks and reduced phase contrast. The moderated aggregation in **P-2F2T** results in improved lateral connectivity across the film, yielding improved grain connectivity and a more uniform surface coverage.

**Figure 6:**
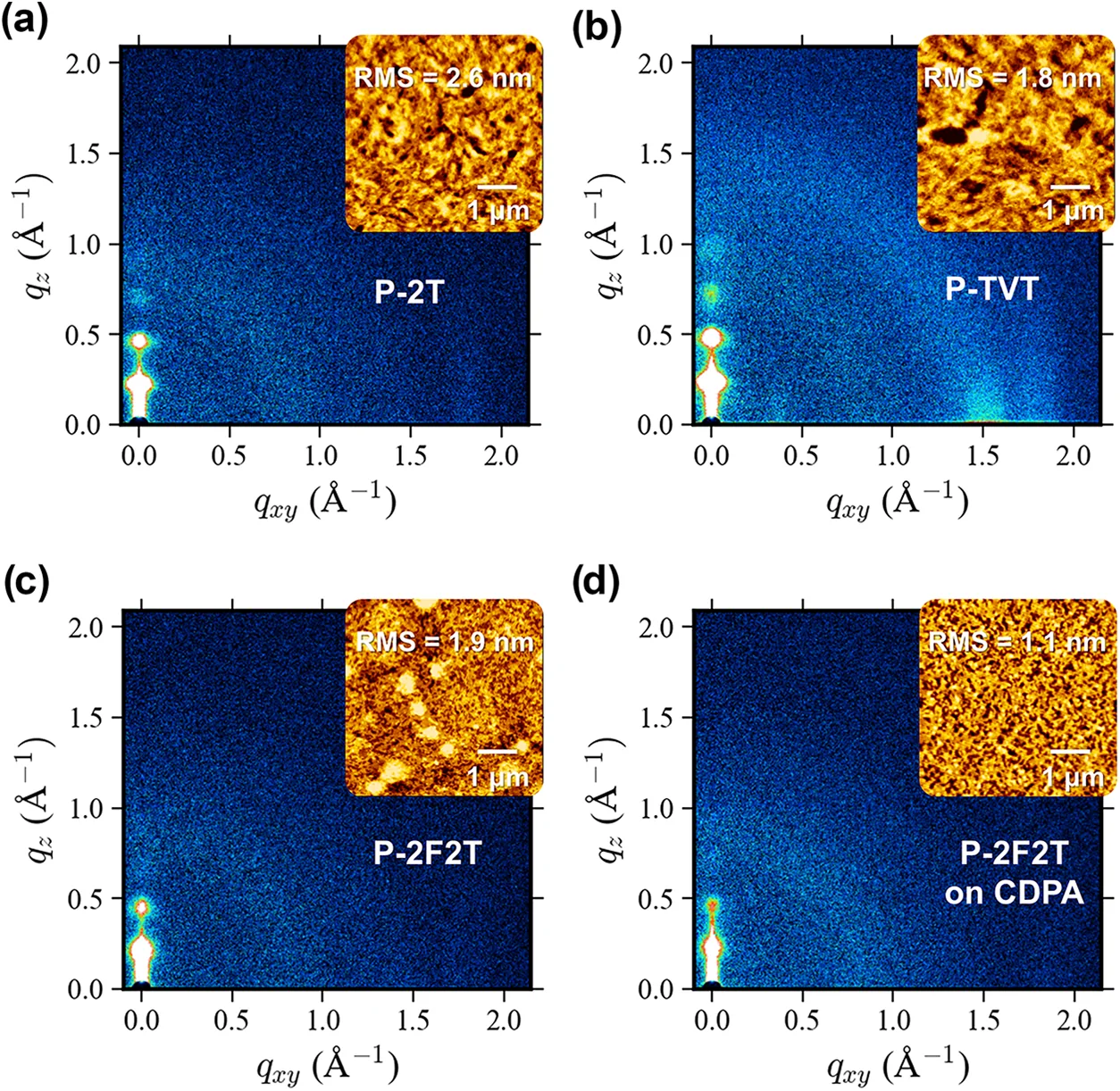
AFM height and GIWAXS images of (a) **P-2T**, (b) **P-TVT**, (c) **P-2F2T** on ODTS, and (d) **P-2F2T** on CDPA. Polymer films were processed under the optimal device fabrication conditions.

Grazing-incidence wide-angle X-ray scattering (GIWAXS) analysis further elucidates the underlying thin-film microstructure. All three polymers adopt a predominant edge-on orientation, which is generally considered to be the optimal orientation for OFET.^32^ The crystal coherence lengths (CCLs) of the **NDPI**-based polymers were calculated using the Sherrer eq (Table S1). The π–π stacking and lamellar stacking related reflections were tentatively assigned as (010) and (100), respectively. Among the series, **P-2T** displays the highest degree of long-range lamellar order (CCL_(100)_ = 147.1 Å), small π–π stacking distance (3.4 Å), and good π-stacking coherence length (CCL_(010)_ = 36.7 Å). The π–π stacking distance of **P-TVT** is expanded to 4.1 Å with a relatively short π-stacking coherence length (CCL_(010)_ = 24.2 Å), indicating weakened interchain electronic coupling along the transport direction. **P-2F2T** on OTDS exhibits reduced lamellar coherence (CCL_(100)_ = 73.1 Å) indicating lower long-range crystallinity, whereas **P-2F2T** on CDPA shows slightly increased lamellar coherence (CCL_(100)_ = 87.2 Å) and contracted lamellar *d*-spacing from 28.7 to 25.6 Å, showcasing a more ordered microstructure.

Correlating morphology, microstructure, and device performance reveals that OFET mobility does not scale directly with crystallinity. Although **P-2T** possesses the highest long-range order, its large and highly aggregated domains may reduce effective interdomain connectivity and contribute to less efficient charge transport. Conversely, **P-2F2T** achieves the highest electron mobility despite reduced crystallinity. Moreover, the fluorine-induced lowering of the LUMO level in **P-2F2T** reduces the electron injection barrier from the gold source-drain electrodes and stabilizes electron transport. These results underscore that optimal OFET performance in this series arises from a synergy between favorable π–π stacking, continuous film morphology, and energy level match, rather than maximized crystalline coherence alone.

## Conclusion

We have developed the first pyrrole-fused naphthalene diimide (**NDPI**) framework and demonstrated its viability as a building block for high-performance donor–acceptor polymers. Pyrrole annulation introduces strong intramolecular donor–acceptor interactions, short π–π stacking distances, and intermolecular hydrogen-bond for supramolecular architectures. **NDPI**-based polymers exhibit narrow optical bandgaps and tunable charge transport ranging from balanced ambipolar to unipolar n-type behavior. Importantly, combined AFM and GIWAXS analyses reveal that device performance is dictated not by crystallinity alone, but by the synergistic interplay of π-stacking and domain connectivity. Collectively, this work establishes pyrrole-fused NDIs as a previously unexplored subclass of core-annulated NDI semiconductors. By combining strong intramolecular donor–acceptor interactions with favorable solid-state organization and competitive device performance, pyrrole annulation emerges as an effective and versatile strategy for advancing NDI-based organic electronic materials.

## Experimental Section

The full experimental details and characterization methods can be found in the Supporting Information.

## Supplementary Material



## Notes

Synthetic procedures and characterization data, additional spectra, X-ray crystallographic data, and DFT calculation details (PDF)
