# Supplementary material for: Dipyrrole-Fused Naphthalene Diimide as a Building Block for High-Performance Organic Semiconductors
Source: J Am Chem Soc. 2026 Jul 17;148(37):39556–64. doi: 10.1021/jacs.6c07621 (PMC13614183; doi:10.1021/jacs.6c07621)
Supplement: Supplementary file 1 [file ja6c07621_si_001.pdf]

Supporting Information for

Dipyrrole-Fused Naphthalene Diimide as a Building Block for High Performance Organic Semiconductors

Kexiang Zhao, Waner He, Xingyu Yang, Yuko Kishida, Hidehiro Uekusa, Zichen Yang, Hidetoshi Matsumoto, Keigo Nonaka, Yoshimitsu Sagara, Juntaro Nogami, Ken Tanaka, Tian-Yu Zhang, Ze-Fan Yao, Tsuyoshi Michinobu**

* Email: zhao495356@outlook.com; michinobu@mct.isct.ac.jp

CORRESPONDENCE ADDRESS: Department of Materials Science and Engineering, Institute of Science Tokyo, 2-12-1 Ookayama, Meguro-ku, Tokyo 152-8552, Japan

Table of Contents

1. **Materials and Measurements** **S2**
2. **Synthetic Details** **S4**
3. **X-ray Crystallography Data** **S20**
4. **CV Curves** **S21**
5. **Photoemission Spectroscopy in Air** **S21**
6. **Optical Spectra** **S22**
7. **Thermal Analysis** **S23**
8. **HT-GPC** **S23**
9. **Computational Details** **S24**
10. **OFET Devices** **S26**
11. **GIWAXS Analysis** **S27**
12. **NMR Spectra** **S28**

1. Materials and Measurements

All commercially available chemicals were used without further purification unless otherwise noted. NDTI-EH was synthesized according to literature procedures.[1] Anhydrous solvents were obtained from an Ultra Solvent Purifior 3-1S-TIM solvent purification system. Column chromatography was performed with silica gel (spherical neutral, particle size 40–100 μm) obtained from Kanto Chemical. Analytical thin-layer chromatography (TLC) was performed on 0.2 mm silica gel-coated glass sheets with F254 indicator. All yields given referred to isolated yields. Nuclear Magnetic Resonance (NMR) spectra were recorded on 400 MHz JEOL JNM-ECZ400S spectrometers. Chemical shifts were reported in ppm. Coupling constants (*J* values) were reported in Hertz. ^{1}H NMR chemical shifts were referenced to TMS (0 ppm), $C_2D_2Cl_4$ (5.98 ppm). ^{13}C NMR chemical shifts were referenced to $CDCl_3$ (77.00 ppm), $C_2D_2Cl_4$ (74.20 ppm). HRMS (ESI) analyses were performed on a Bruker micro-TOF Focus II instrument. HRMS (FD) analyses were performed on a JEOL JMS-T2000GC instrument.

UV–vis spectra were recorded on a JASCO V-670 spectrometer. In the case of thin-film measurements, samples were spin-coated onto precleaned quartz substrates from their chlorobenzene solutions. The films were then annealed on a hot plate at 100 °C for 5 minutes to drive off the residual solvent. Photoluminescence spectra were recorded on a JASCO FP8500 spectrometer. Absolute photoluminescent quantum yield was determined using the HAMAMATSU compact fluorescence lifetime spectrometer C11367. Photoluminescent decays were measured on HAMAMATSU absolute PL quantum yield spectrometer C11347. Cyclic voltammetry (CV) of **5a** and **5b** was carried out in 0.1 M *n*-Bu_4NPF_6 solution in dichloromethane (DCM) using a three-electrode configuration on a BAS electrochemical analyzer model 612C (glassy carbon as working electrode, Pt as counter electrode, and Ag/AgCl as reference). The ferrocene/ferrocenium (Fc/Fc^+) couple was served as external reference. After the acquirement of E_{Fc}, the value of HOMO/LUMO energy level (relative to E_{VAC}) can be calculated applying empirical formulas $E_{HOMO} = -[4.8 - E_{Fc} + E_{ox}^{onset}]$ and $E_{LUMO} = -[4.8 - E_{Fc} + E_{re}^{onset}]$ (eV). CV of polymer films was carried out in 0.1 M *n*-Bu_4NPF_6 solution in acetonitrile using the same setup. The optical bandgap was calculated from the intercept of the normalized absorption spectra using $E_g^{opt} = 1240\ nm/\lambda_{intercept}$. HOMO energy levels of polymers were determined by photoemission spectroscopy on an AC2S spectrometer (RIKEN KEIKI) as thin films. The LUMO energy levels of polymers were calculated using $E_{LUMO} = E_{HOMO} + E_g^{opt}$ (eV). Thermogravimetric analysis (TGA) was carried out on a Rigaku Thermoplus TG8120 under N_2 (10 °C min^{-1}).

The AFM samples at various conditions were analyzed by a Seiko Instruments SPA-400 with a Seiko Instruments DF-20 stiff cantilever. Two-dimensional (2D) grazing incidence wide-angle X-ray scattering (GIWAXS) patterns were measured at SPring-8 on beamline BL40B2 (Hyogo, Japan). The samples were irradiated at a fixed incident angle on the order of 0.10° and the GIWAXS patterns were recorded with a photon-counting detector (Eiger2 S 500K, Dectris, Ltd.). The wavelength of the X-ray beam was 1.0 Å (energy of 12.39 KeV)

and the camera length was 108.6016 mm. The components of the scattering vector, q, parallel and perpendicular to the sample surface were defined as q_{xy} and q_z, respectively.

The magnitude of scattering vector q is defined as:

$$q = \frac{4\pi \sin\theta}{\lambda}$$

where q is the scattering vector magnitude; θ is the half of the scattering angle; λ is the X-ray wavelength (1.0 Å). In GIWAXS profiles, diffraction peaks appear at specific q-values corresponding to periodic molecular spacings in the film. The relation between reciprocal space (q) and real-space distance (d) is expressed as:

$$d = \frac{2\pi}{q}$$

Scherrer's equation was used to determine the crystalline coherence length (CCL) as follows:

$$\text{CCL} = \frac{K\lambda}{\beta \cos\theta}$$

where K is a dimensionless shape factor (0.94), θ is the Bragg angle, and β is the full width at half-maximum (FWHM) of the scattering peak.

2. Synthetic Details

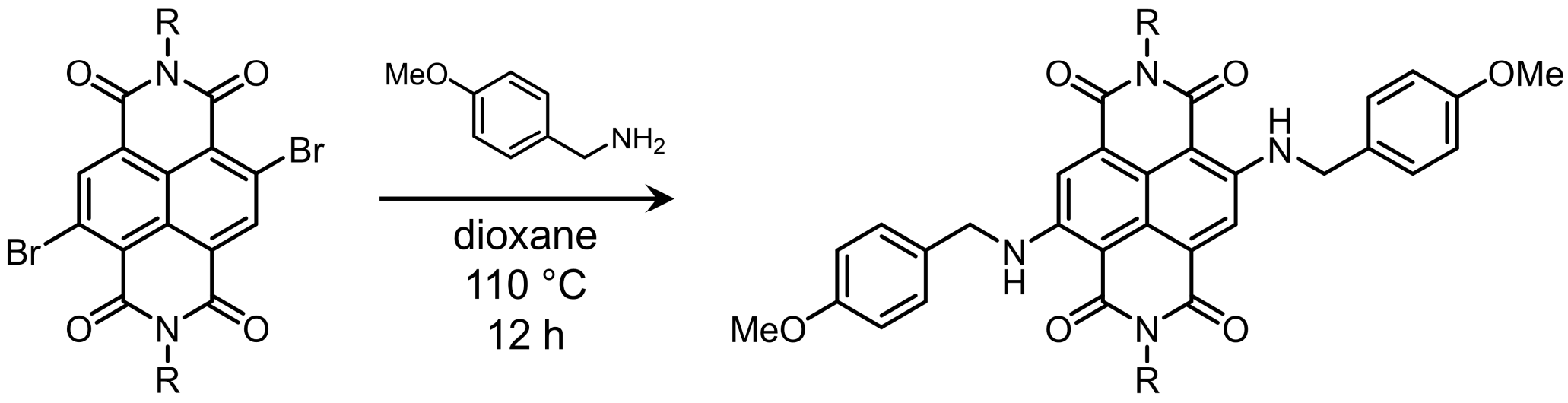

NDI-2Br

General Procedure I (GP I). The 2,6-dibromo NDI (1 mmol) was added to a Schlenk flask. Then the flask was evacuated and refilled with N_2 for three times followed by the addition of dry dioxane (30 mL) and *p*-methoxybenzyl (PMB) amine (10 eq, 10 mmol) under N_2. The flask was placed in an oil bath and heated to 110 °C for 12 h with stirring. After the reaction mixture was cooled to room temperature, the solvent was removed in vacuo. The residue was washed by methanol, water, and methanol again. The solid was dried and further purified by column chromatography to yield the pure product.

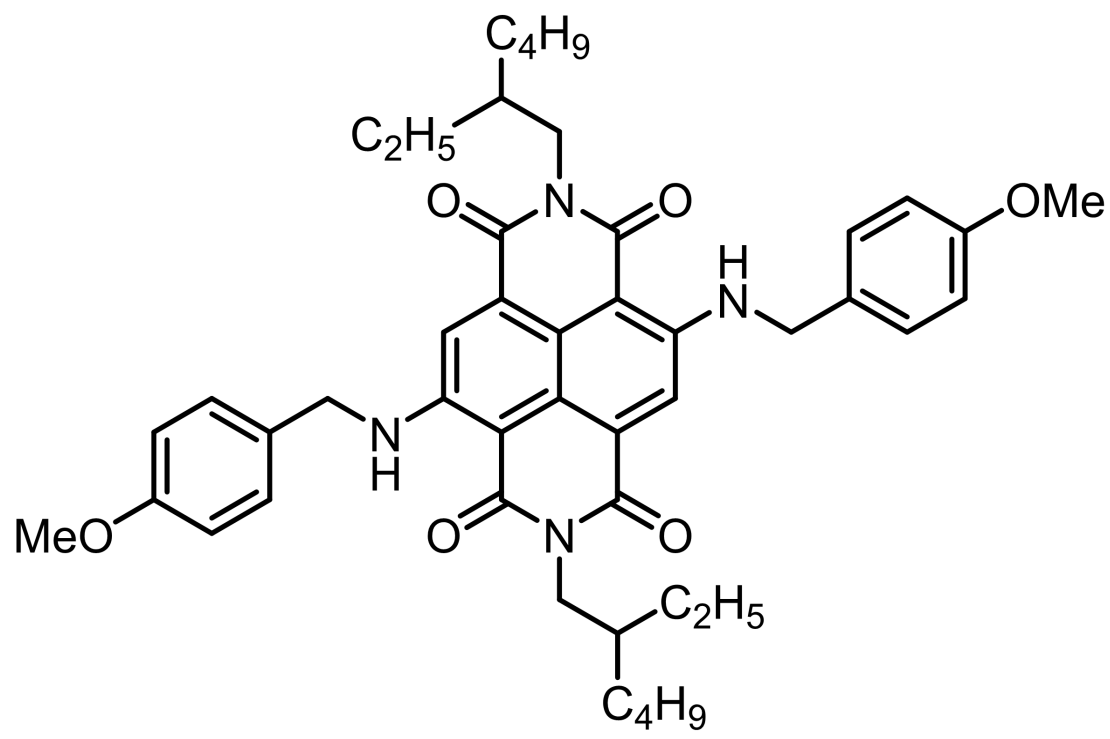

2,7-bis(2-ethylhexyl)-4,9-bis((4-methoxybenzyl)amino) benzo[lmn][3,8] phenanthrol- -ine -1,3,6,8(2H,7H)-tetraone. GP I was applied to NDI-2Br-EH (1 mmol, 648.4 mg) and PMB amine (10 eq, 10 mmol, 1.3 mL). Column chromatography over silica gel (DCM/Hex = 1:1 to 1:0) yielded the product as dark blue solids (733 mg, 95%). ^{1}H NMR (400 MHz, $CDCl_3$) δ 9.71 (t, J = 5.7 Hz, 2H), 8.18 (s, 2H), 7.35 (d, J = 8.7 Hz, 4H), 6.91 (d, J = 8.7 Hz, 4H), 4.65 (d, J = 5.6 Hz, 4H), 4.20 – 3.96 (m, 4H), 3.80 (s, 6H), 1.92 (p, J = 6.5 Hz, 2H), 1.42 – 1.21 (m, 16H), 0.98 – 0.80 (m, 12H). ^{13}C NMR (100 MHz, $CDCl_3$) δ 166.56, 163.37, 159.09, 148.99, 129.58, 128.64, 125.76, 121.34, 118.70, 114.31, 102.29, 55.29, 46.56, 44.11, 37.77, 30.64, 28.57, 23.99, 23.12, 14.11, 10.70. HRMS (FD-MS) *m/z*: $[M]^+$ calcd for $C_{46}H_{56}N_4O_6$, 760.4194; found, 760.4145.

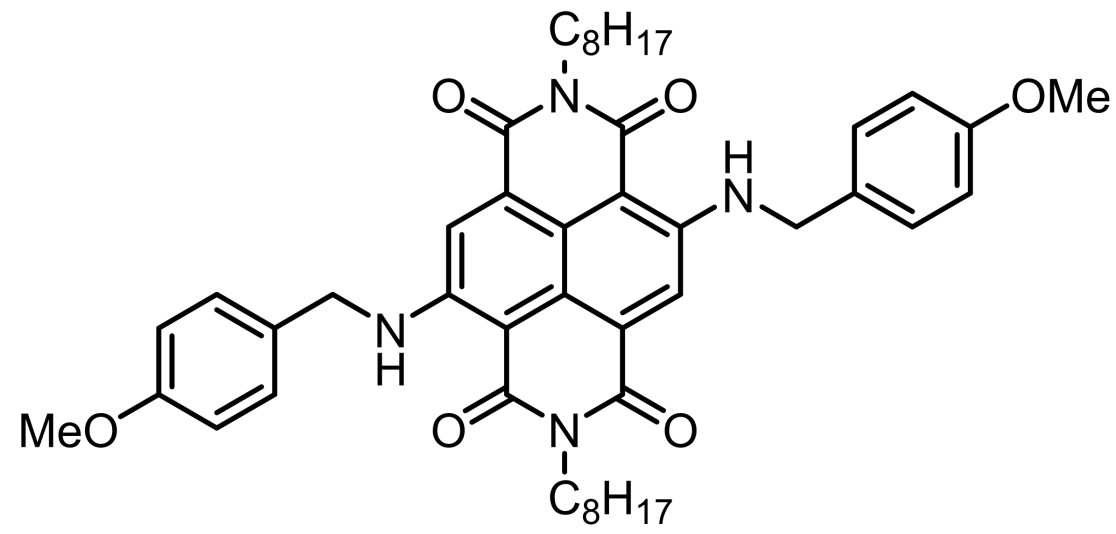

4,9-bis((4-methoxybenzyl)amino)-2,7-dioctylbenzo[lmn][3,8]phenanthroline-1,3,6,8 (2H,7H)-tetraone. GP I was applied to NDI-2Br-Oct (1 mmol, 648.4 mg) and PMB amine (10 eq, 10 mmol, 1.3 mL). Column chromatography over silica gel (DCM/Hex = 1:1 to 1:0) yielded the product as dark blue solids (760 mg, 99%). ^{1}H NMR (400 MHz, $CDCl_3$) δ 9.57 (t, *J* = 5.4 Hz, 2H), 8.03 (s, 2H), 7.38 (d, *J* = 8.6 Hz, 4H), 6.92 (d, *J* = 8.6 Hz, 4H), 4.57 (d, *J* = 5.4 Hz, 4H), 4.18 – 4.00 (m, 4H), 3.80 (s, 6H), 1.72 – 1.62 (m, 4H), 1.44 – 1.23 (m, 20H), 0.92 – 0.82 (m, 6H). ^{13}C NMR (100 MHz, $CDCl_3$) δ 165.96, 162.68, 159.14, 148.66, 129.45, 128.91, 125.49, 120.93, 118.24, 114.33, 101.95, 55.27, 46.67, 40.54, 31.82, 29.70, 29.36, 29.26, 28.07, 27.24, 22.65, 14.12. HRMS (ESI-MS) *m/z*: $[M+Na]^+$ calcd for $C_{46}H_{56}N_4O_6Na$, 783.4092; found, 783.4091.

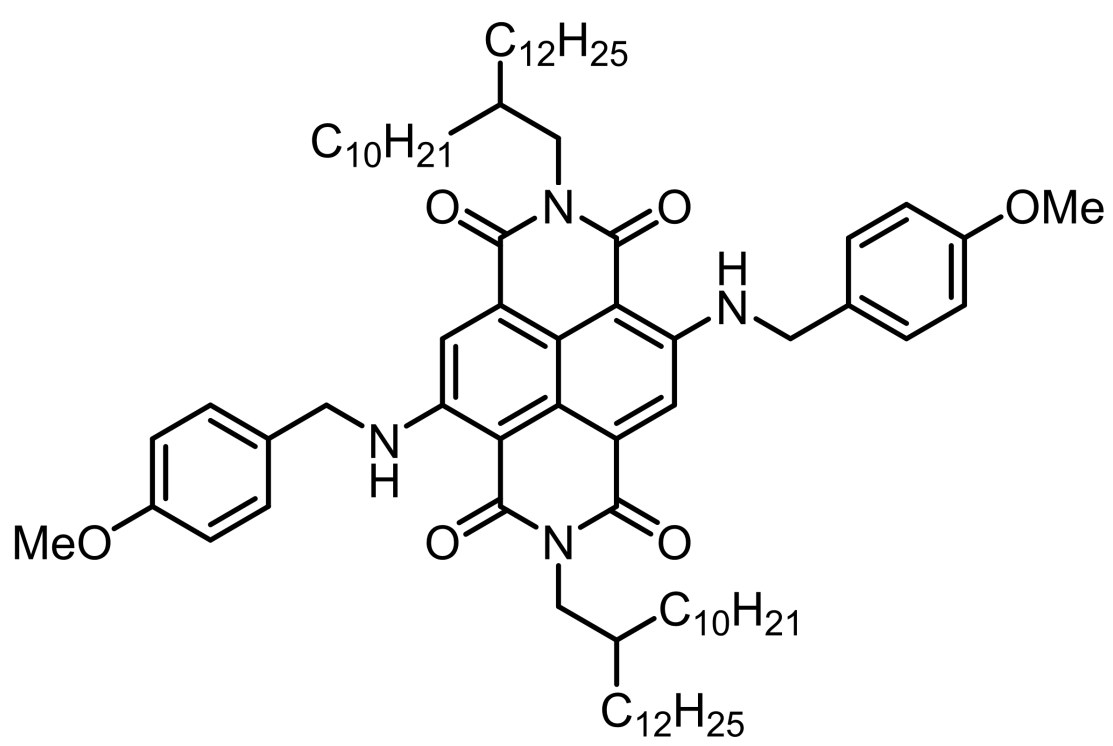

2,7-bis(2-decyltetradecyl)-4,9-bis((4-methoxybenzyl)amino) benzo[lmn][3,8] phenan--throline-1,3,6,8(2H,7H)-tetraone. GP I was applied to NDI-2Br-DT (1 mmol, 1.10 g) and PMB amine (10 eq, 10 mmol, 1.3 mL). Column chromatography over silica gel (DCM/Hex = 1:2 to 1:1) yielded the product as dark blue solids (1.20 g, 99%). ^{1}H NMR (400 MHz, $CDCl_3$) δ 9.68 (t, *J* = 5.7 Hz, 2H), 8.13 (s, 2H), 7.35 (d, *J* = 8.1 Hz, 4H), 6.91 (d, *J* = 8.0 Hz, 4H), 4.63 (d, *J* = 5.4 Hz, 4H), 4.06 (d, *J* = 7.3 Hz, 4H), 3.80 (s, 6H), 1.95 (p, *J* = 6.2 Hz, 2H), 1.46 – 1.10 (m, 80H), 0.95 – 0.77 (m, 12H). ^{13}C NMR (100 MHz, $CDCl_3$) δ 166.47, 163.25, 159.07, 148.88, 129.54, 128.68, 125.65, 121.23, 118.58, 114.28, 102.16, 55.25, 46.57, 44.40, 36.46, 31.92, 31.57, 30.10, 29.71, 29.67, 29.65, 29.64, 29.38, 29.36, 26.42, 22.70, 14.15. HRMS (FD-MS) *m/z*: $[M]^+$ calcd for $C_{78}H_{120}N_4O_6$, 1208.9202; found, 1208.9214.

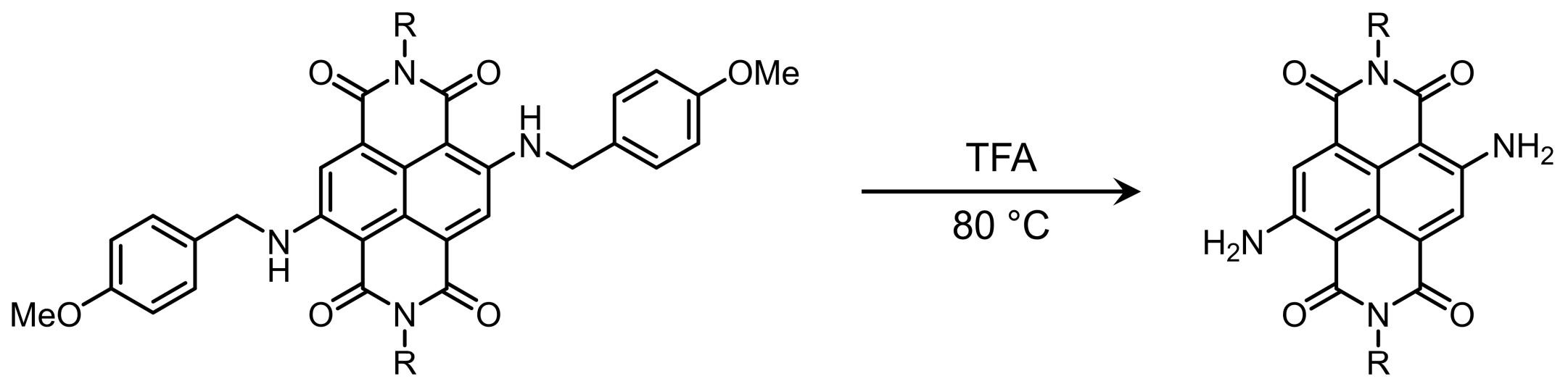

NDI-2NHPMB **1**

General Procedure II (GP II): NDI-2NHPMB (0.5 mmol) was added to a pressure tube followed by the addition of trifluoroacetic acid (TFA). The tube was then sealed and heated to 80 °C in an oil bath with stirring. When cooled to room temperature, TFA was removed in vacuo. For **1a** and **1b**, the residue was boiled in ethanol overnight and filtered hot. The solid was washed with more ethanol and dried under vacuum to yield the product. For **1c**, the residue was subjected to column chromatography over silica gel to furnish the product.

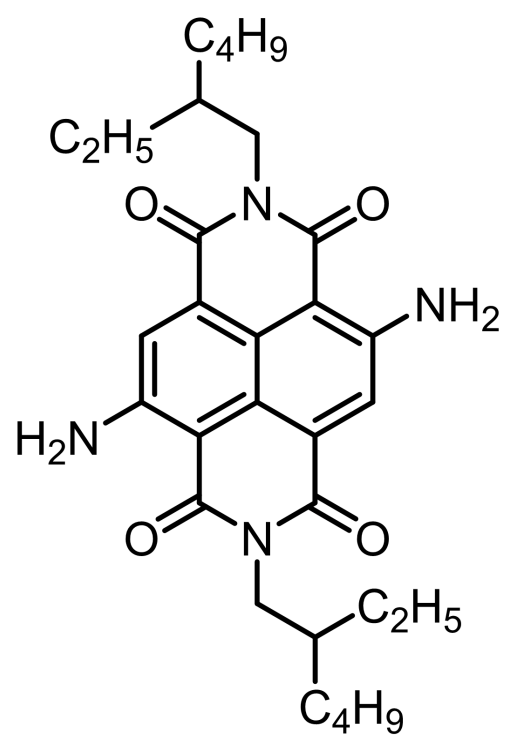

4,9-diamino-2,7-bis(2-ethylhexyl)benzo[lmn][3,8]phenanthroline-1,3,6,8(2H,7H)-tetraone (1a). GP II was applied to the NDI-2NHPMB-EH (0.5 mmol, 380 mg) in 3.8 mL TFA for 4 hours to yield violet solids (234 mg, 90%). Satisfactory NMR spectra were not obtained due to the poor solubility of **1a** in common solvents. HRMS (FD-MS) *m/z*: $[M]^+$ calcd for $C_{30}H_{40}N_4O_4$, 520.30441; found, 520.30754.

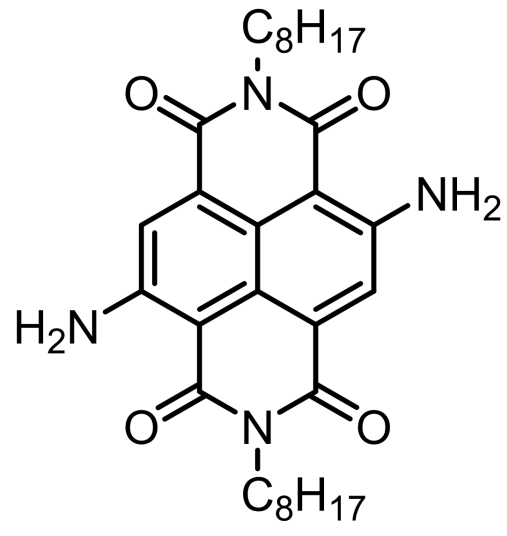

4,9-diamino-2,7-dioctylbenzo[lmn][3,8]phenanthroline-1,3,6,8(2H,7H)-tetraone (1b). GP II was applied to the NDI-2NHPMB-Oct (0.5 mmol, 380 mg) in 3.8 mL TFA for 4 hours to yield violet solids (242 mg, 93%). Satisfactory NMR spectra were not obtained due to the poor solubility of **1a** in common solvents. HRMS (FD-MS) *m/z*: $[M]^+$ calcd for $C_{30}H_{40}N_4O_4$, 520.30441; found, 520.30752.

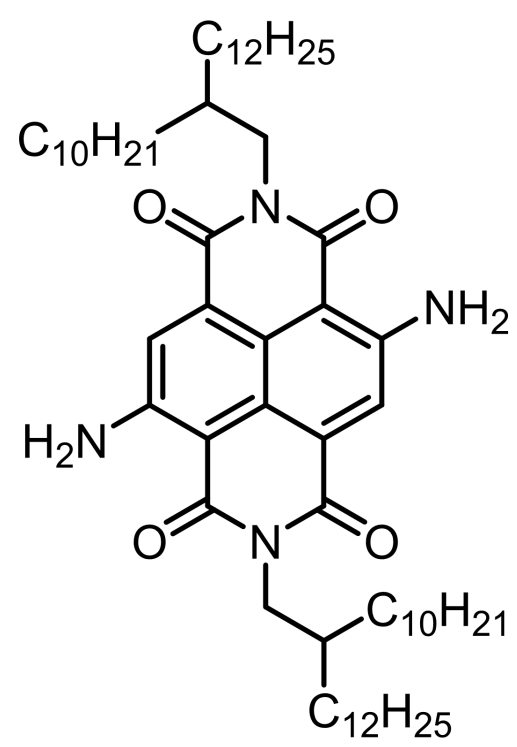

4,9-diamino-2,7-bis(2-decyltetradecyl)benzo[lmn][3,8]phenanthroline-1,3,6,8 (2H,7H)-tetraone (1c). GP II was applied to the NDI-2NHPMB-DT (0.5 mmol, 605 mg) in 3.8 mL TFA for 12 hours. Column chromatography over silica gel (DCM/Hex = 2:1 to 1:0) yielded violet solids (369 mg, 76%). ^1H NMR (400 MHz, $CDCl_3$) δ 7.98 (s, 2H), 6.87 (s, 4H), 4.09 (d, *J* = 7.3 Hz, 4H), 1.97 (p, *J* = 6.1 Hz, 2H), 1.45 – 1.16 (m, 81H), 0.97 – 0.77 (m, 12H). ^{13}C NMR (100 MHz, $CDCl_3$) δ 166.30, 163.27, 148.87, 125.96, 123.28, 121.84, 102.99, 44.52, 36.67, 31.96, 31.86, 30.09, 29.71, 29.68, 29.66, 29.65, 29.38, 29.36, 26.56, 22.70, 14.07. HRMS (FD-MS) *m/z*: $[M]^+$ calcd for $C_{62}H_{104}N_4O_4$, 968.80521; found, 968.80196.

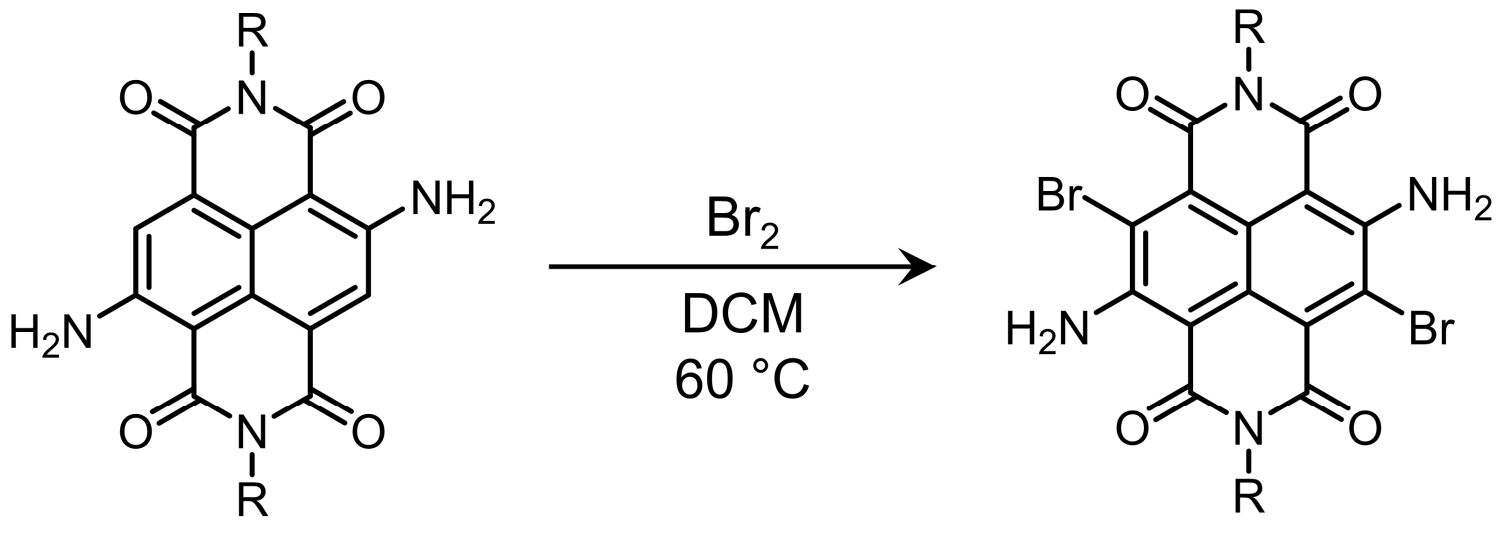

1 **2**

General Procedure III (GP III): Compound **1** was added to a pressure tube followed by the addition of dichloromethane (DCM) and elemental bromine. The tube was then sealed and heated to 60 °C in an oil bath with stirring for 12 hours. When cooled to room temperature, the reaction mixture was carefully added into cold saturated aqueous solution of Na_2SO_3 with stirring. Chloroform was added to dilute the mixture, and the organic phase was separated. The aqueous phase was further extracted with chloroform twice. The organic phase was combined and washed with saturated aqueous solution of $NaHCO_3$ then brine. The combined organic phase was dried over Na_2SO_4 before the solvent was removed in vacuo. The residue was subjected to column chromatography to furnish the compound **2**.

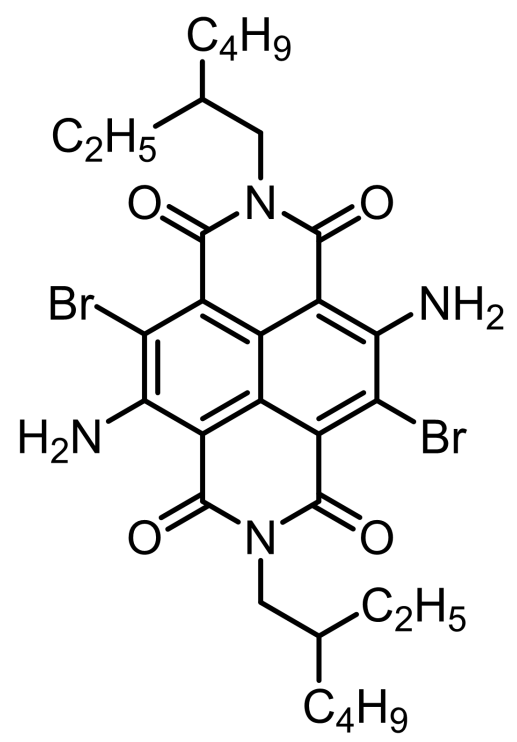

4,9-diamino-5,10-dibromo-2,7-bis(2-ethylhexyl)benzo[lmn][3,8]phenanthroline-1,3,6,8(2H,7H)-tetraone (2a). GP III was applied to compound **1a** (0.3 mmol, 156 mg) and bromine (2 mL) in 6 mL DCM. Column chromatography over silica gel (DCM/Hex = 1:1 to 2:1) yielded compound **2a** as magenta solids (179 mg, 87%). ^{1}H NMR (400 MHz, $CDCl_3$) δ 9.97 (s, 2H), 6.43 (s, 2H), 4.16 (dt, J = 9.5, 5.0 Hz, 4H), 1.94 (p, J = 6.8 Hz, 2H), 1.47 – 1.19 (m, 16H), 1.02 – 0.78 (m, 12H). ^{13}C NMR (100 MHz, $CDCl_3$) δ 161.25, 147.81, 122.92, 121.74, 121.22, 101.73, 44.85, 37.63, 30.60, 28.51, 23.96, 23.13, 14.11, 10.66. HRMS (FD-MS) m/z: $[M]^+$ calcd for $C_{30}H_{38}N_4O_4Br_2$, 676.12543; found, 676.11987.

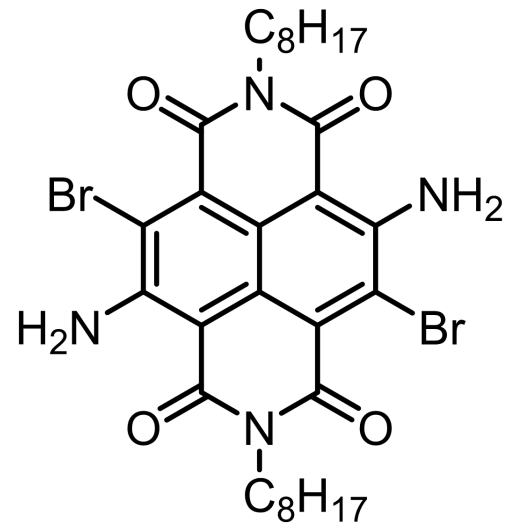

4,9-diamino-5,10-dibromo-2,7-dioctylbenzo[lmn][3,8]phenanthroline-1,3,6,8(2H, 7H)-tetraone (2b). GP III was applied to compound **1b** (0.3 mmol, 156 mg) and bromine (2 mL) in 6 mL DCM. Column chromatography over silica gel (DCM/Hex = 1:1 to 2:1) yielded compound **2b** as magenta solids (177 mg, 86%). ^{1}H NMR (400 MHz, $CDCl_3$, 328 K) δ 4.24 – 4.10 (m, 4H), 1.72 (p, *J* = 7.4 Hz, 4H), 1.45 – 1.20 (m, 20H), 0.97 – 0.77 (m, 6H). The amino protons were not observed due to the peak broadening at elevated temperature. ^{13}C NMR (100 MHz, $CDCl_3$, 328 K) δ 164.63, 160.89, 147.97, 123.07, 121.70, 121.35, 101.94, 41.56, 31.87, 29.37, 29.27, 27.99, 27.29, 22.66, 14.03. HRMS (FD-MS) *m/z*: $[M]^+$ calcd for $C_{30}H_{38}N_4O_4Br_2$, 676.12543; found, 676.12487.

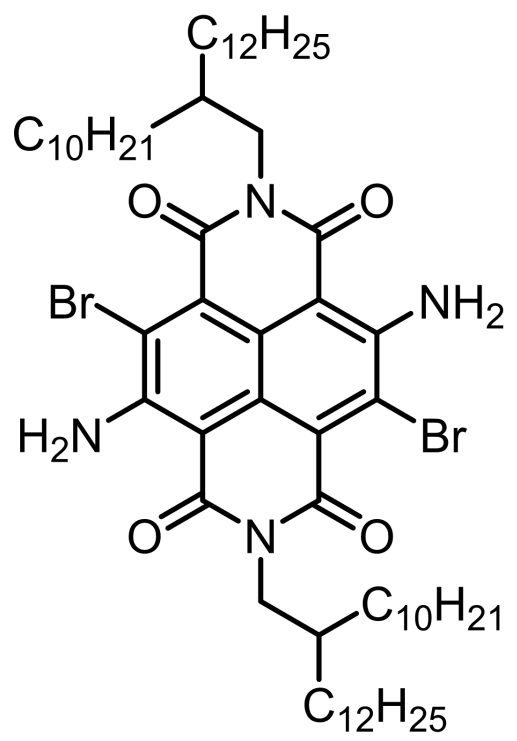

4,9-diamino-5,10-dibromo-2,7-dioctylbenzo[lmn][3,8]phenanthroline-1,3,6,8(2H, 7H)-tetraone (2c). GP III was applied to compound **1c** (0.3 mmol, 291 mg) and bromine (2 mL) in 6 mL DCM. Column chromatography over silica gel (DCM/Hex = 1:1 to 2:1) yielded compound **2c** as magenta solids (301 mg, 89%). ^{1}H NMR (400 MHz, $CDCl_3$) δ 9.94 (s, 2H), 6.41 (s, 2H), 4.15 (d, *J* = 7.4 Hz, 4H), 1.97 (p, *J* = 6.1 Hz, 2H), 1.45 – 1.13 (m, 80H), 0.97 – 0.77 (m, 12H). ^{13}C NMR (100 MHz, $CDCl_3$) δ 164.85, 161.17, 147.76, 122.85, 121.70, 121.13, 101.65, 45.11, 36.30, 31.93, 31.52, 30.07, 29.70, 29.69, 29.67, 29.64, 29.61, 29.38, 29.36, 26.33, 22.70, 14.15. HRMS (FD-MS) *m/z*: $[M]^+$ calcd for $C_{62}H_{102}N_4O_4Br_2$, 1124.62623; found, 1124.62847.

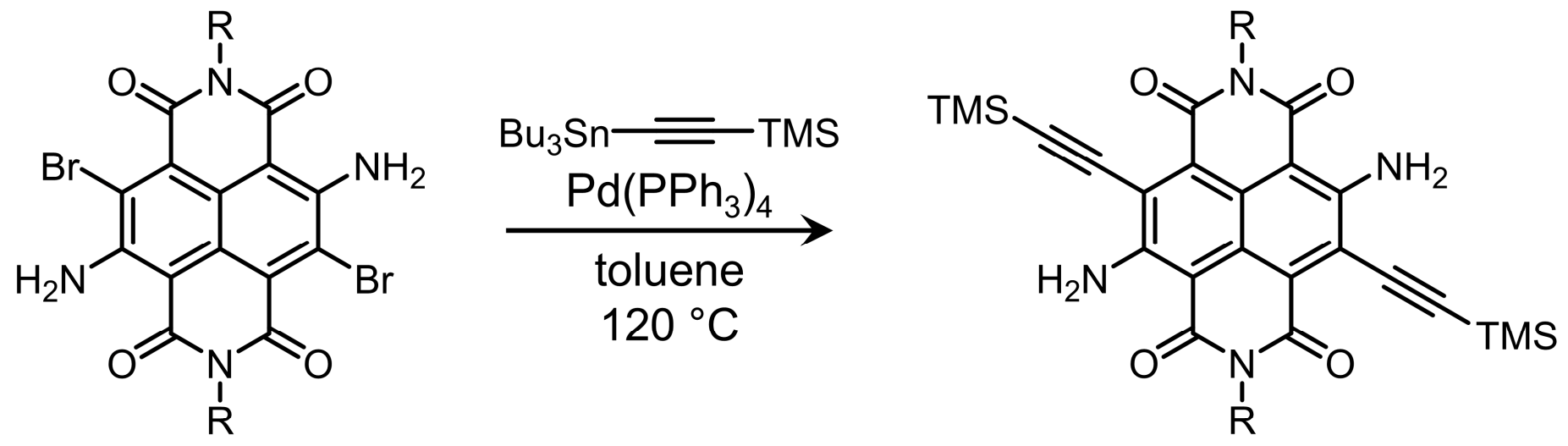

2 **3**

General Procedure IV (GP IV): Compound **2** (0.2 mmol, 1 eq) and $Pd(PPh_3)_4$ (10 mol%, 0.02 mmol, 23.1 mg) were placed in a Schlenk flask under N_2 followed by the addition of dry toluene (10 mL) and trimethylsilylacetylene tin reagent (2.5 eq, 0.75 mmol, 290 mg). The Schlenk flask was heated to 120 ℃ in an oil bath with stirring for 4 hours. When cooled to room temperature, the solvent was removed in vacuo. The residue was subjected to column chromatography to furnish the compound **3**.

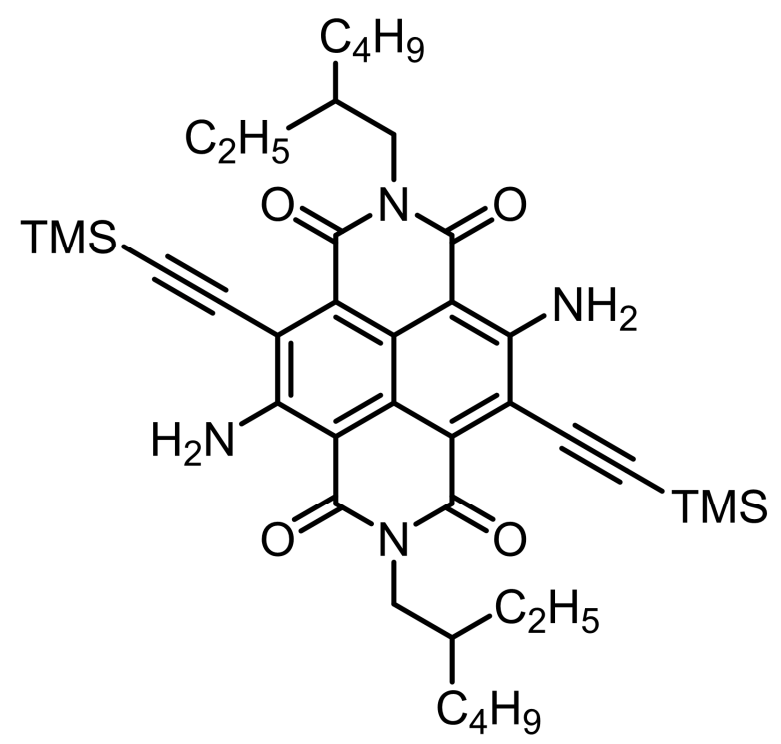

4,9-diamino-2,7-bis(2-ethylhexyl)-5,10- bis((trimethylsilyl)ethynyl) benzo[lmn] [3,8] phenanthroline-1,3,6,8(2H,7H)-tetraone (3a). GP IV was applied to compound **2a** (0.2 mmol, 136 mg). Column chromatography over silica gel (DCM/Hex = 1:3 to 1:2) yielded compound **3a** as blue solids (112 mg, 79%). ^{1}H NMR (400 MHz, $CDCl_3$) δ 9.51 (s, 2H), 6.45 (s, 2H), 4.29 – 4.00 (m, 4H), 1.95 (p, J = 6.5 Hz, 2H), 1.47 – 1.22 (m, 16H), 1.01 – 0.79 (m, 12H), 0.39 (s, 17H).^{13}C NMR (100 MHz, $CDCl_3$) δ 165.91, 161.80, 150.04, 125.67, 120.66, 116.78, 115.50, 101.16, 100.35, 44.06, 37.68, 30.65, 28.52, 24.08, 23.17, 14.24, 10.71, -0.02. HRMS (ESI-MS) *m/z*: $[M+Na]^+$ calcd for $C_{40}H_{56}N_4O_4Si_2Na$, 735.3732; found, 735.3753.

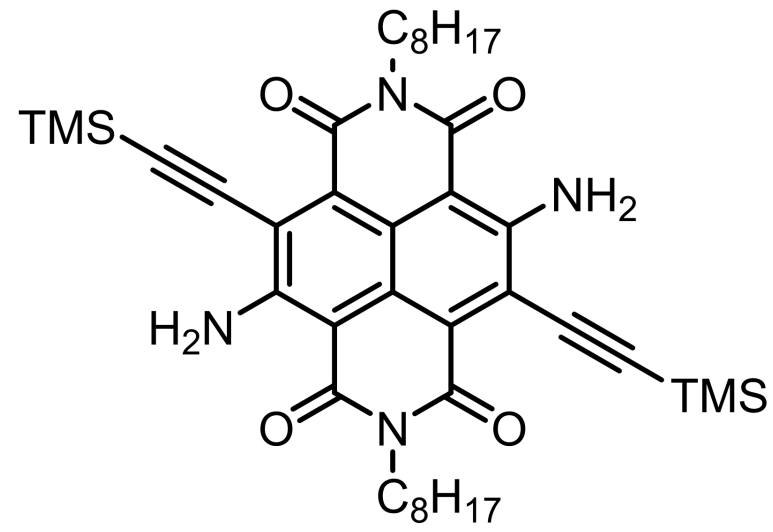

4,9-diamino-2,7-dioctyl-5,10- bis((trimethylsilyl)ethynyl)benzo[lmn][3,8] phenanthr- -oline-1,3,6,8(2H,7H)-tetraone (3b). GP IV was applied to compound **2b** (0.2 mmol, 136 mg). Column chromatography over silica gel (DCM/Hex = 1:3 to 1:2) yielded compound **3b** as blue solids (114 mg, 80%). ^{1}H NMR (400 MHz, $CDCl_3$) δ 9.51 (s, 2H), 6.44 (s, 2H), 4.27 – 4.04 (m, 4H), 1.80 – 1.65 (m, 4H), 1.49 – 1.20 (m, 20H), 0.96 – 0.80 (m, 6H), 0.41 (s, 17H). ^{13}C NMR (100 MHz, $CDCl_3$) δ 165.47, 161.41, 150.03, 125.52, 120.55, 116.69, 115.62, 101.14, 100.30, 40.73, 31.93, 29.39, 29.32, 27.96, 27.26, 22.74, 14.18, 0.03. HRMS (ESI-MS) *m/z*: $[M+Na]^+$ calcd for $C_{40}H_{56}N_4O_4Si_2Na$, 735.3732; found, 735.3750.

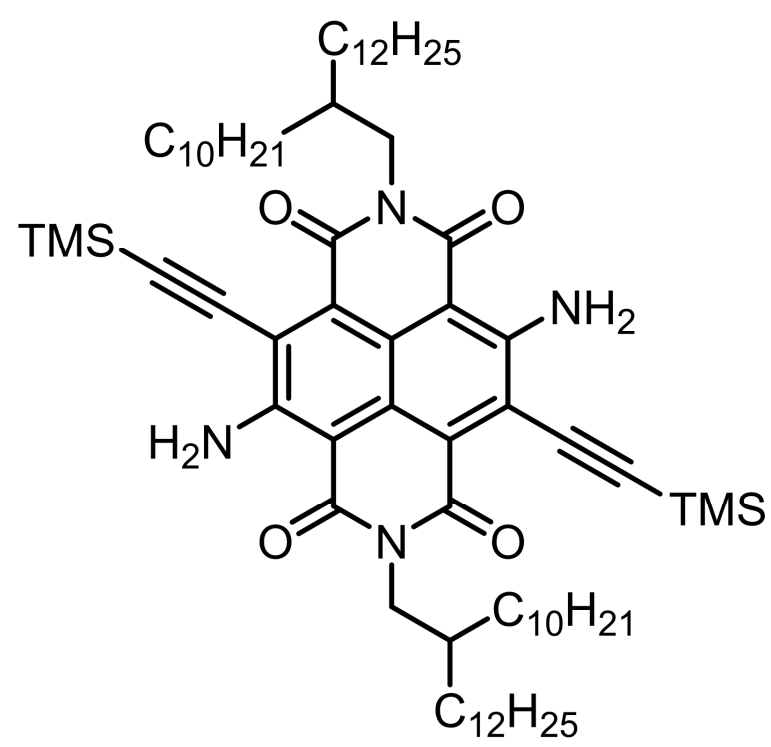

4,9-diamino-2,7-bis(2-decyltetradecyl)-5,10- bis((trimethylsilyl)ethynyl)benzo[lmn] [3,8]phenanthroline-1,3,6,8(2H,7H)-tetraone (3c). GP IV was applied to compound **2c** (0.2 mmol, 225 mg). Column chromatography over silica gel (DCM/Hex = 1:3 to 1:2) yielded compound **3c** as blue solids (209 mg, 90%). ^{1}H NMR (400 MHz, $CDCl_3$) δ 9.50 (s, 2H), 6.43 (s, 2H), 4.12 (d, *J* = 7.2 Hz, 4H), 1.99 (p, *J* = 6.2 Hz, 2H), 1.51 – 1.11 (m, 81H), 0.97 – 0.76 (m, 12H), 0.40 (s, 18H). ^{13}C NMR (100 MHz, $CDCl_3$) δ 165.95, 161.78, 150.09, 125.70, 120.69, 116.81, 115.52, 101.19, 100.49, 44.50, 36.44, 32.14, 31.77, 30.28, 29.92, 29.91, 29.88, 29.59, 26.53, 22.91, 14.36, 0.12. HRMS (ESI-MS) *m/z*: $[M+Na]^+$ calcd for $C_{72}H_{120}N_4O_4Si_2Na$, 1183.8740; found, 1183.8756.

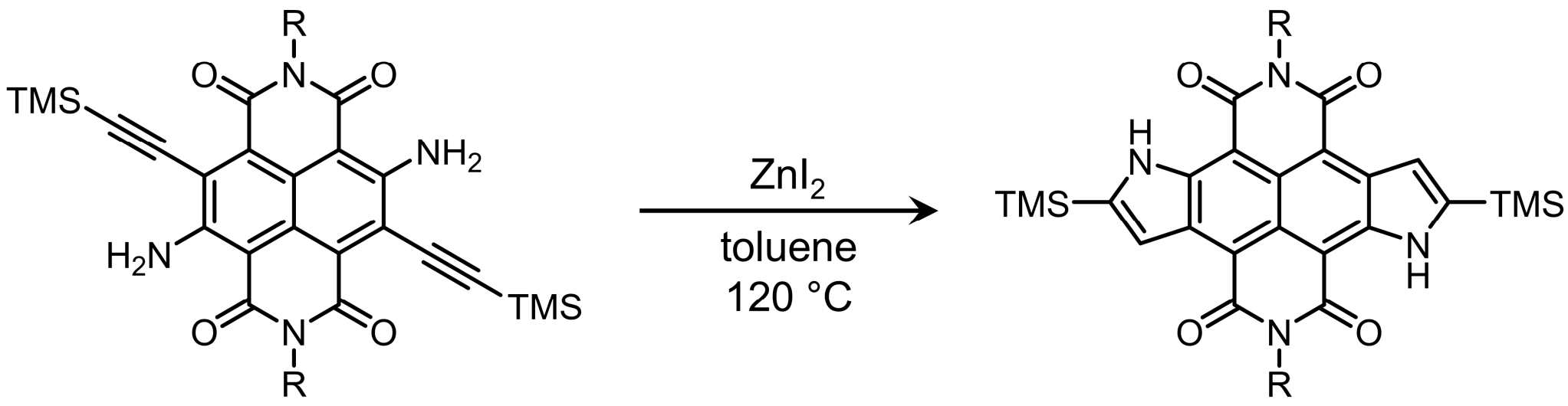

3 **4**

General Procedure V (GP V): Compound **3** (0.1 mmol, 1 eq) and ZnI_2 (2.5 eq, 0.25 mmol, 80 mg) were placed in a Schlenk flask under N_2 followed by the addition of dry toluene (10 mL). The Schlenk flask was heated to 120 °C in an oil bath with stirring for 12 hours. When cooled to room temperature, water was added to dissolve the remaining ZnI_2. The organic phase was separated and the aqueous phase was extracted with DCM. The combined organic phase was dried over Na_2SO_4 before the solvent was removed in vacuo. The residue was subjected to column chromatography to furnish the compound **4**.

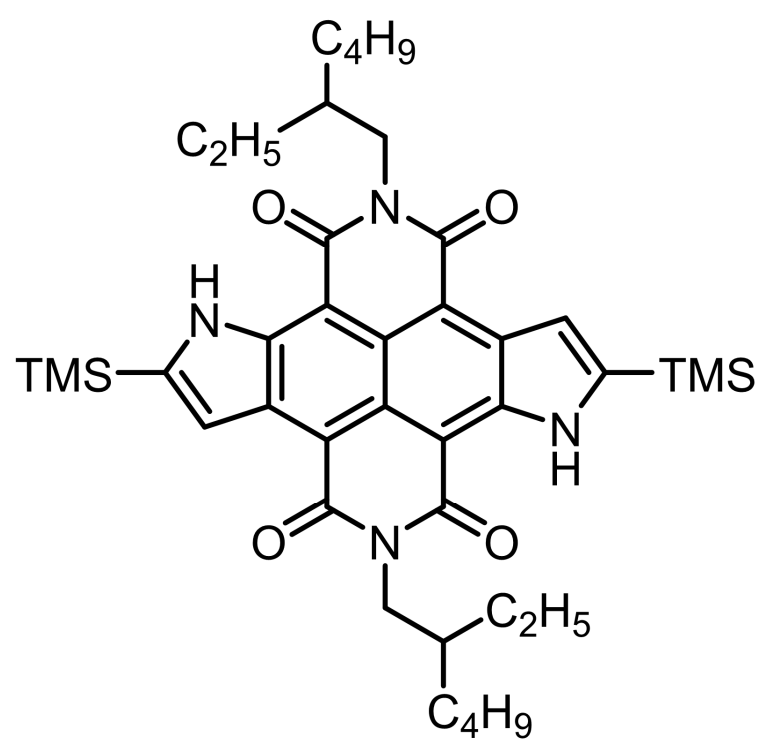

2,8-bis(2-ethylhexyl)-5,11-bis(trimethylsilyl)-4,10-dihydroindolo[4,5,6,7- lmn] pyrro--lo[3,2-f][3,8]phenanthroline-1,3,7,9(2H,8H)-tetraone (4a). GP V was applied to compound **3a** (0.1 mmol, 71 mg). Column chromatography over silica gel (DCM/Hex = 1:2 to 1:1) yielded compound **4a** as dark green solids (60 mg, 84%). ^{1}H NMR (400 MHz, $CDCl_3$) δ 10.80 (s, 2H), 7.96 (d, J = 2.3 Hz, 2H), 4.37 – 4.18 (m, 4H), 2.11 (p, J = 6.8 Hz, 2H), 1.52 – 1.27 (m, 16H), 0.97 (t, J = 7.4 Hz, 6H), 0.88 (t, J = 7.0 Hz, 6H), 0.51 (s, 18H). ^{13}C NMR (100 MHz, $CDCl_3$) δ 165.89, 164.72, 153.04, 140.89, 133.51, 119.69, 114.96, 112.43, 105.85, 44.18, 37.84, 30.72, 28.65, 24.05, 23.19, 14.12, 10.78, -1.33. HRMS (FD-MS) *m/z*: $[M+Na]^+$ calcd for $C_{40}H_{56}N_4O_4Si_2Na$, 735.3732; found, 735.3750.

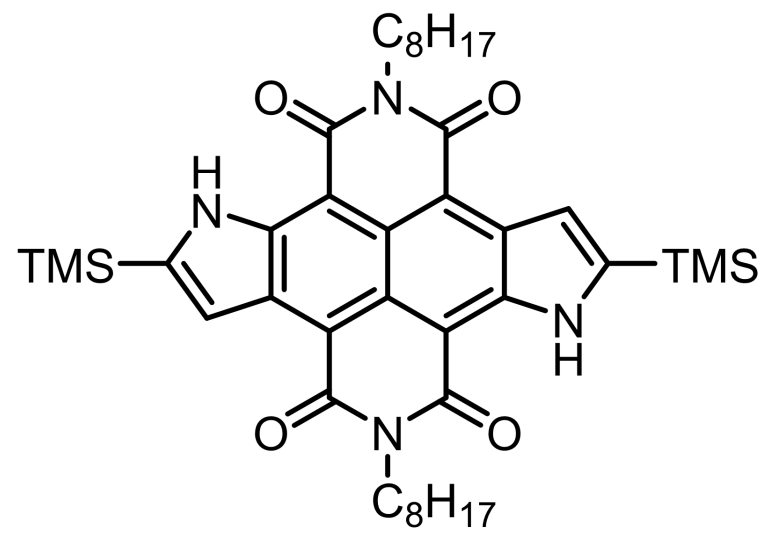

2,8-dioctyl-5,11-bis(trimethylsilyl)-4,10-dihydroindolo[4,5,6,7-lmn]pyrrolo[3,2-f][3,8]phenanthroline-1,3,7,9(2H,8H)-tetraone (4b). GP V was applied to compound **3b** (0.1 mmol, 71 mg). Column chromatography over silica gel (DCM/Hex = 1:2 to 1:1) yielded compound **4b** as dark green solids (56 mg, 78%). ^{1}H NMR (400 MHz, $CDCl_3$) δ 10.74 (s, 2H), 7.92 (t, J = 2.1 Hz, 2H), 4.30 (dd, J = 9.1, 6.5 Hz, 4H), 1.83 (p, J = 8.2 Hz, 4H), 1.56 – 1.26 (m, 20H), 0.96 – 0.79 (m, 6H), 0.52 (s, 18H). ^{13}C NMR (100 MHz, $CDCl_3$) δ 165.38, 164.23, 153.15, 140.74, 133.36, 119.42, 114.78, 112.29, 105.72, 40.46, 31.84, 29.44, 29.30, 28.34, 27.38, 22.65, 14.12, -1.36. HRMS (ESI-MS) *m/z*: $[M+Na]^+$ calcd for $C_{40}H_{56}N_4O_4Si_2Na$, 735.3732; found, 735.3737.

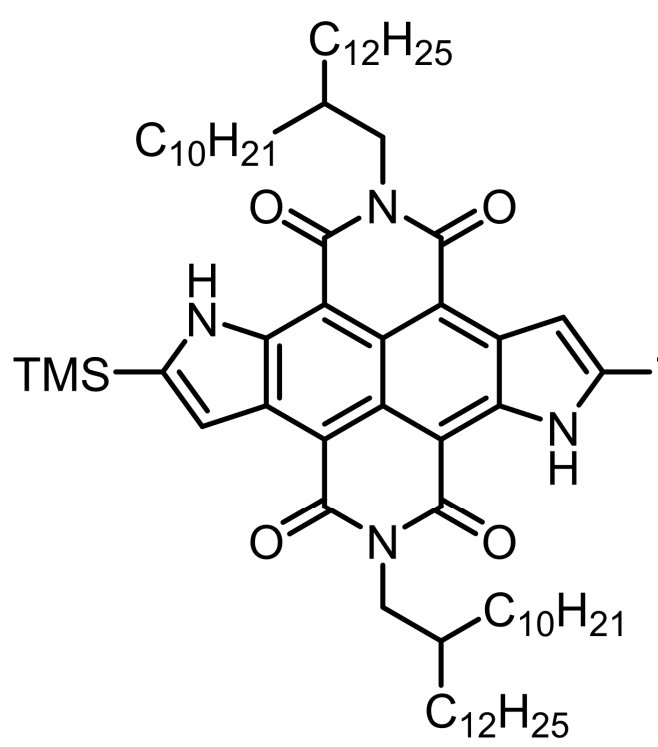

2,8-bis(2-decyltetradecyl)-5,11-bis(trimethylsilyl)-4,10-dihydroindolo[4,5,6,7-lmn]pyrrolo[3,2-f][3,8]phenanthroline-1,3,7,9(2H,8H)-tetraone (4c). GP V was applied to compound **3c** (0.1 mmol, 116 mg). Column chromatography over silica gel (DCM/Hex = 1:3 to 1:2) yielded compound **4c** as dark green solids (100 mg, 86%). ^{1}H NMR (400 MHz, $CDCl_3$) δ 10.75 (s, 2H), 7.93 (d, J = 2.3 Hz, 2H), 4.25 (d, J = 7.5 Hz, 4H), 2.20 – 2.06 (m, 2H), 1.52 – 1.10 (m, 80H), 0.94 – 0.76 (m, 12H), 0.53 (s, 18H). ^{13}C NMR (100 MHz, $CDCl_3$) δ 165.71, 164.49, 152.88, 140.76, 133.38, 119.50, 114.79, 112.44, 105.66, 44.42, 36.52, 31.91, 31.89, 31.62, 31.59, 30.14, 30.13, 29.66, 29.64, 29.61, 29.35, 29.32, 26.49, 22.69, 22.67, 14.14, 14.12, -1.29. HRMS (ESI-MS) *m/z*: $[M]^+$ calcd for $C_{72}H_{120}N_4O_4Si_2$, 1160.88426; found, 1160.88960.

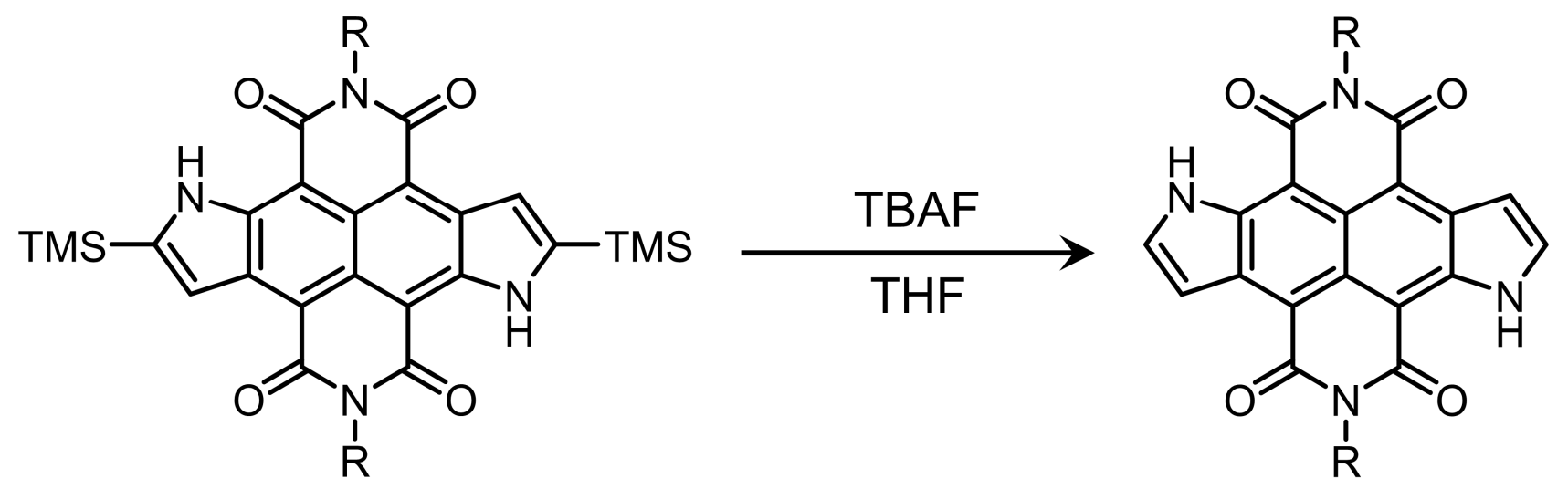

4 **5**

General Procedure VI (GP VI): Compound **4** (0.05 mmol, 1 eq) was dissolved in THF (1 mL) followed by the addition of 1 M tetrabutylammonium fluoride (TBAF) solution in THF (2.2 eq, 110 μL). After 30 min, methanol was added to precipitate the product. The solid was collected by filtration and further purified by column chromatography to furnish the compound **5**.

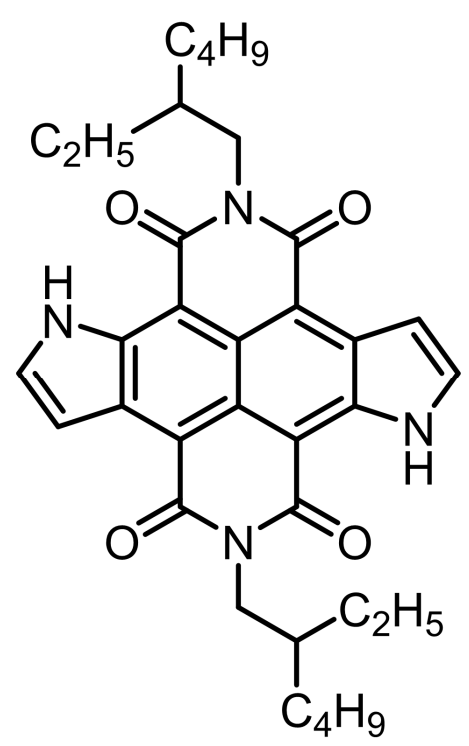

2,8-bis(2-ethylhexyl)-4,10-dihydroindolo[4,5,6,7-lmn]pyrrolo[3,2- f][3,8] phenanthr--oline-1,3,7,9(2H,8H)-tetraone (5a). GP VI was applied to compound **4a** (0.05 mmol, 36 mg). Column chromatography over silica gel (DCM/Hex = 2:1 to 1:0) yielded compound **5a** as dark burgundy solids (24 mg, 84%). ^{1}H NMR (400 MHz, $CDCl_3$) δ 10.83 (s, 2H), 7.82 (dt, *J* = 13.0, 3.0 Hz, 4H), 4.33 – 4.17 (m, 4H), 2.06 (p, *J* = 6.5 Hz, 2H), 1.47 – 1.25 (m, 16H), 0.96 (t, *J* = 7.4 Hz, 6H), 0.87 (t, *J* = 7.1 Hz, 6H). ^{13}C NMR (100 MHz, 1,1,2,2-$C_2D_2Cl_4$, 363 K) δ 165.83, 164.49, 138.55, 135.02, 133.03, 119.89, 116.07, 106.67, 104.86, 44.28, 38.30, 31.23, 28.91, 24.57, 23.17, 14.07, 10.96. HRMS (FD-MS) *m/z*: $[M]^+$ calcd for $C_{34}H_{40}N_4O_4$, 568.30441; found, 568.30793.

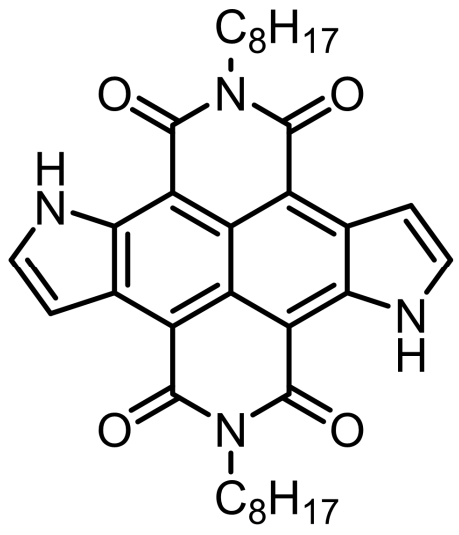

2,8-bis(2-ethylhexyl)-4,10-dihydroindolo[4,5,6,7-lmn]pyrrolo[3,2- f][3,8] phenanthr--oline-1,3,7,9(2H,8H)-tetraone (5b). GP VI was applied to compound **4b** (0.05 mmol, 36 mg). Column chromatography over silica gel (DCM/Hex = 2:1 to 1:0) yielded compound **5b** as dark burgundy solids (28 mg, 97%). ^{1}H NMR (400 MHz, 1,1,2,2-$C_2D_2Cl_4$, 363 K) δ 10.81 (s, 2H), 7.84 (dt, *J* = 15.4, 2.9 Hz, 4H), 4.38 – 4.24 (m, 4H), 1.86 (p, *J* = 7.3 Hz, 4H), 1.57 – 1.41 (m, 8H), 1.40 – 1.28 (m, 12H), 0.91 (t, 6H). ^{13}C NMR (100 MHz, 1,1,2,2-$C_2D_2Cl_4$, 363 K) δ 165.40, 164.10, 138.46, 135.10, 132.94, 119.75, 116.00, 106.61, 104.77, 40.44, 31.91, 29.47, 29.29, 28.43, 27.40, 22.68, 14.09. HRMS (FD-MS) *m/z*: $[M]^+$ calcd for $C_{34}H_{40}N_4O_4$, 568.30441; found, 568.30788.

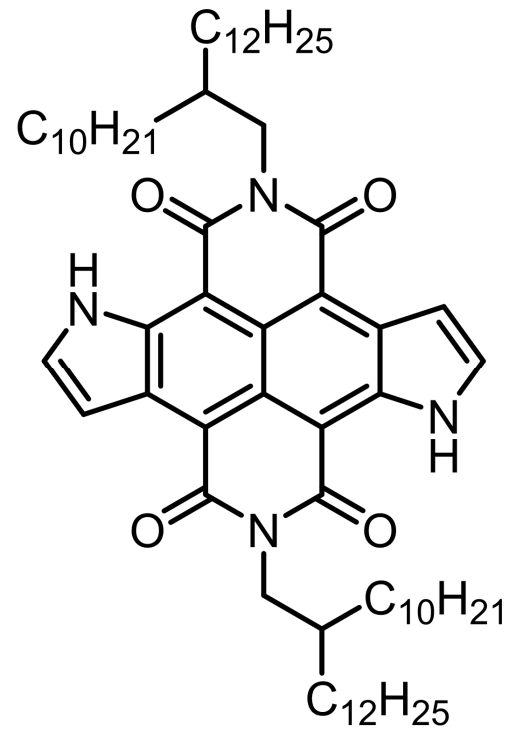

2,8-bis(2-decyltetradecyl)-4,10-dihydroindolo[4,5,6,7-lmn]pyrrolo[3,2- f][3,8] phena--nthroline-1,3,7,9(2H,8H)-tetraone (5c). GP VI was applied to compound **4c** (0.05 mmol, 58 mg). Column chromatography over silica gel (DCM/Hex = 1:1 to 2:1) yielded compound **5c** as dark burgundy solids (43 mg, 85%). ^{1}H NMR (400 MHz, $CDCl_3$) δ 10.70 (s, 2H), 7.83 – 7.74 (m, 2H), 7.72 – 7.64 (m, 2H), 4.17 (d, *J* = 7.3 Hz, 4H), 2.04 (p, *J* = 6.3 Hz, 2H), 1.51 – 1.08 (m, 80H), 0.94 – 0.77 (m, 12H). ^{13}C NMR (100 MHz, $CDCl_3$) δ 165.32, 164.08, 137.83, 134.76, 132.37, 119.02, 115.14, 105.83, 104.45, 44.23, 36.58, 31.92, 31.90, 31.64, 30.08, 29.68, 29.65, 29.63, 29.61, 29.36, 29.33, 26.47, 22.69, 22.67, 14.13. HRMS (FD-MS) *m/z*: $[M]^+$ calcd for $C_{66}H_{104}N_4O_4$, 1016.80521; found, 1016.80505.

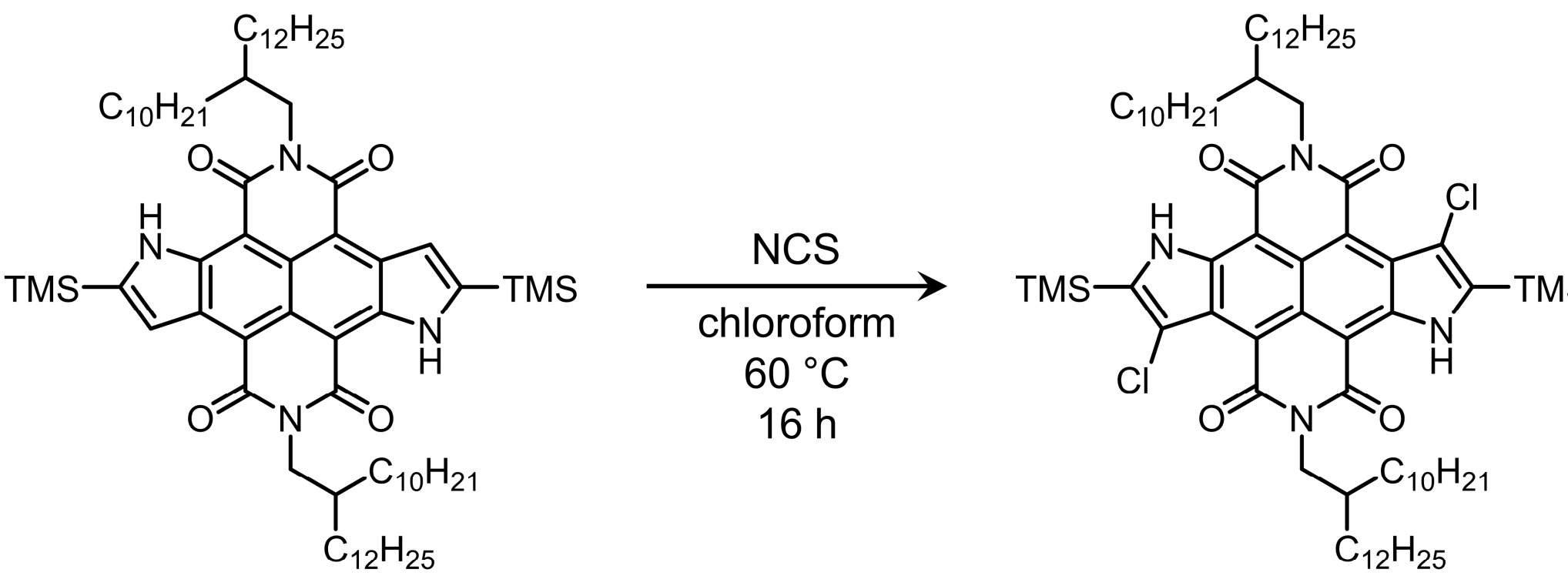

4c **6**

6,12-dichloro-2,8-bis(2-decyltetradecyl)-5,11-bis(trimethylsilyl)-4,10- dihydroindolo [4,5,6,7-lmn]pyrrolo[3,2-f][3,8]phenanthroline-1,3,7,9(2H,8H)-tetraone (6). Compound **4c** (0.1 mmol, 116 mg) was dissolved in 10 mL chloroform followed by the addition of N-chlorosuccinimide (NCS, 2.1 eq, 28 mg). The mixture was heated to 60 °C in an oil bath with stirring for 16 hours. After cooling to room temperature, Na_2SO_3 aqueous solution was added to the mixture. The organic phase was separated and the aqueous phase was extracted with DCM twice. The combined organic phase was dried over Na_2SO_4 before the solvent was removed in vacuo. The residue was subjected to column chromatography over silica gel (DCM/Hex = 1:2) to furnish the compound **6** as dark green semi-solids (116 mg, 94%). ^{1}H NMR (400 MHz, $CDCl_3$) δ 11.20 (s, 2H), 4.25 (d, J = 7.3 Hz, 4H), 2.11 (p, J = 6.4 Hz, 2H), 1.52 – 1.12 (m, 80H), 0.94 – 0.79 (m, 12H), 0.60 (s, 18H). ^{13}C NMR (100 MHz, $CDCl_3$) δ 165.54, 162.39, 149.64, 140.07, 129.51, 119.98, 116.49, 115.34, 105.85, 44.34, 36.40, 31.92, 31.90, 31.54, 30.08, 29.69, 29.65, 29.63, 29.37, 29.34, 26.39, 22.70, 14.14, -1.97. HRMS (ESI-MS) *m/z*: $[M+Na]^+$ calcd for $C_{72}H_{118}N_4O_4Si_2Na$, 1251.7961; found, 1251.7949.

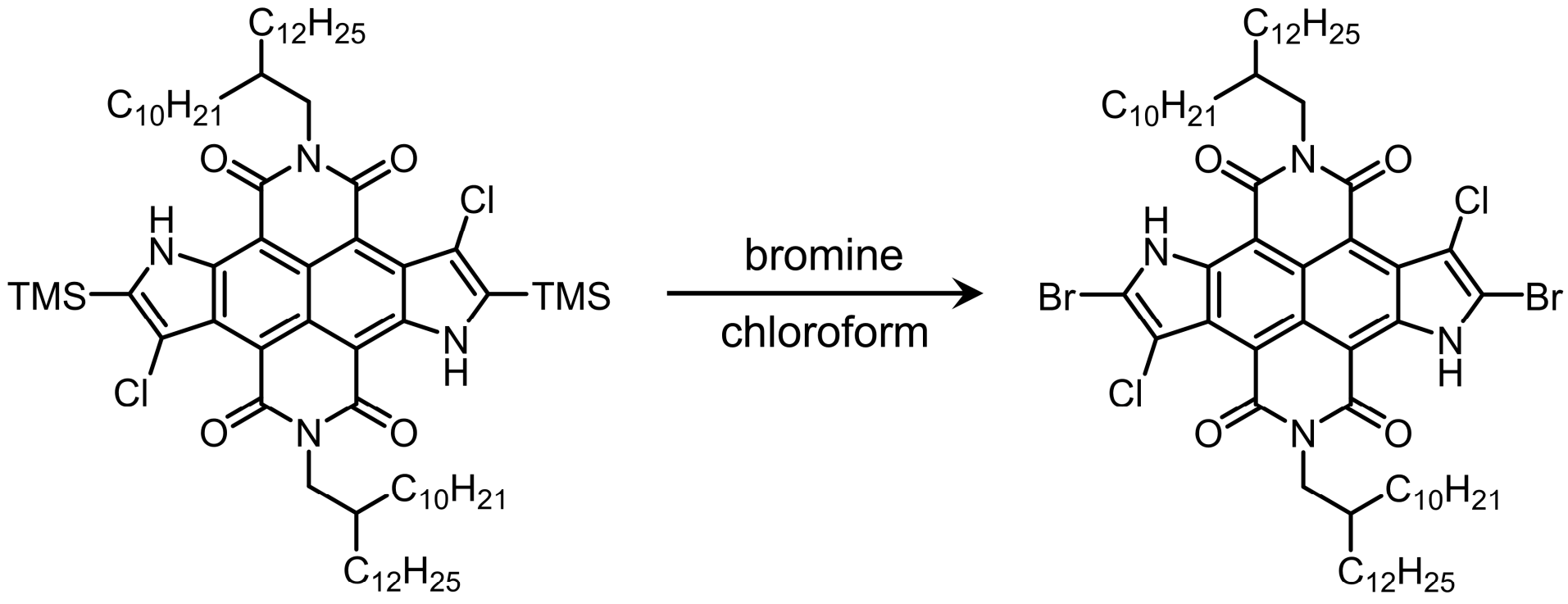

6 **7**

5,11-dibromo-6,12-dichloro-2,8-bis(2-decyltetradecyl)-4,10-dihydroindolo[4,5,6,7-lmn]pyrrolo[3,2-f][3,8]phenanthroline-1,3,7,9(2H,8H)-tetraone (7). Bromine (25 μL) in 1 mL chloroform was slowly added to an ice-cold solution of compound **7** (0.05 mmol, 62 mg) in 4 mL chloroform. After stirring for 8 hours, Na_2SO_3 aqueous solution was added to quench the excessive bromine. The organic phase was separated and the aqueous phase was extracted with chloroform twice. The combined organic phase was dried over Na_2SO_4 before the solvent was removed in vacuo. The residue was subjected to column chromatography over silica gel (DCM/Hex = 1:1) to furnish the compound **7** as dark green semi-solids (47 mg, 76%). ^{1}H NMR (400 MHz, $CDCl_3$) δ 11.38 (s, 2H), 4.17 (d, *J* = 7.4 Hz, 4H), 2.02 (p, *J* = 6.4 Hz, 2H), 1.48 – 1.11 (m, 80H), 0.94 – 0.78 (m, 12H). ^{13}C NMR (100 MHz, $CDCl_3$) δ 164.80, 161.72, 137.52, 128.10, 124.32, 119.35, 115.46, 108.88, 105.28, 44.50, 36.56, 31.93, 31.91, 31.60, 30.07, 29.69, 29.67, 29.63, 29.37, 29.34, 26.42, 22.70, 22.69, 14.14. HRMS (FD-MS) *m/z*: $[M]^+$ calcd for $C_{66}H_{100}N_4O_4Br_2Cl_2$, 1240.54829; found, 1240.55260.

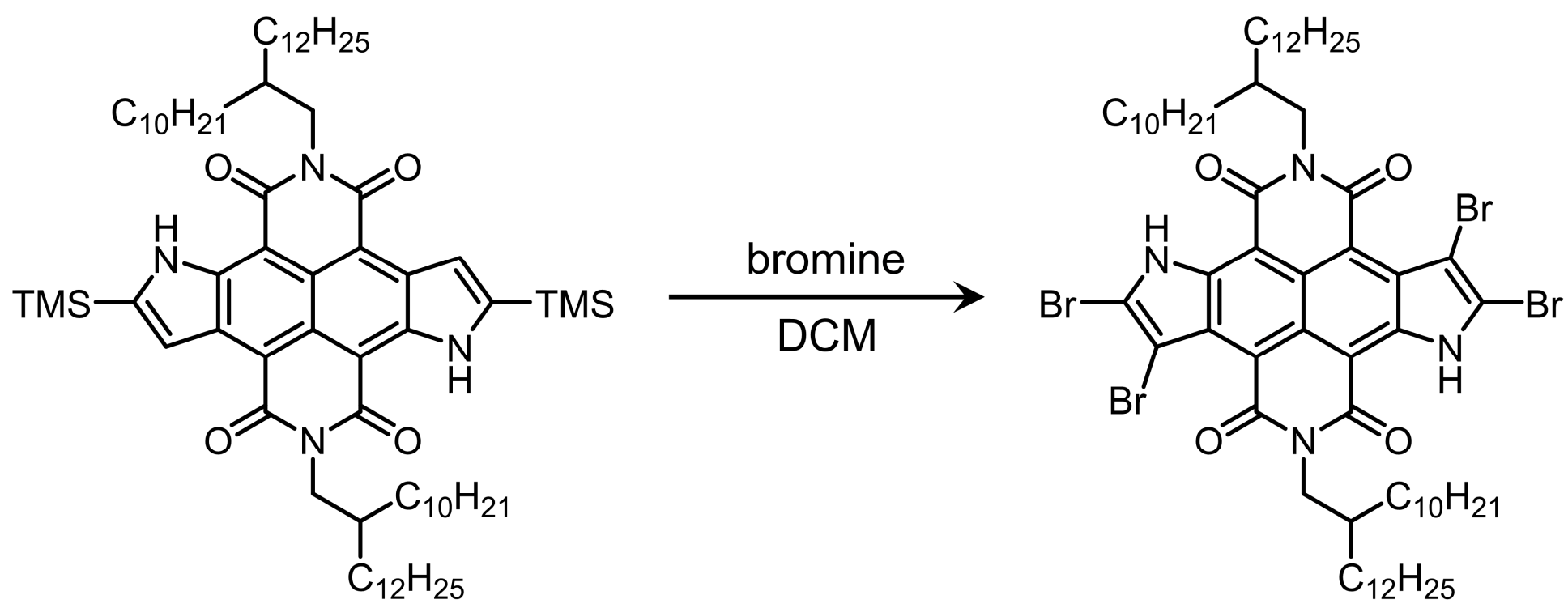

4c

5,6,11,12-tetrabromo-2,8-bis(2-decyltetradecyl)-4,10-dihydroindolo[4,5,6,7-lmn]pyr-- rolo[3,2-f][3,8]phenanthroline-1,3,7,9(2H,8H)-tetraone. Bromine (10 eq, 13 μL) was added to a solution of compound **4c** (0.025 mmol, 29 mg) in 2.5 mL DCM. After stirring for 10 min, Na_2SO_3 aqueous solution was added to quench the excessive bromine. The organic phase was separated and the aqueous phase was extracted with chloroform twice. The combined organic phase was dried over Na_2SO_4 before the solvent was removed in vacuo. The residue was subjected to column chromatography over silica gel (DCM/Hex = 1:1) to furnish the product as dark green solids (19 mg, 56%). ^{1}H NMR (400 MHz, $CDCl_3$) δ 11.46 (s, 2H), 4.19 (d, *J* = 7.3 Hz, 4H), 2.03 (p, *J* = 6.5 Hz, 2H), 1.46 – 1.14 (m, 80H), 0.93 – 0.80 (m, 12H). ^{13}C NMR (100 MHz, $CDCl_3$) δ 164.84, 161.78, 138.46, 130.07, 127.46, 119.40, 115.45, 105.32, 94.44, 44.50, 36.68, 31.93, 31.91, 31.73, 30.08, 29.69, 29.66, 29.64, 29.37, 29.34, 26.50, 22.70, 22.68, 14.13. HRMS (FD-MS) *m/z*: $[M]^+$ calcd for $C_{66}H_{100}N_4O_4Br_4$, 1328.44726; found, 1328.45688.

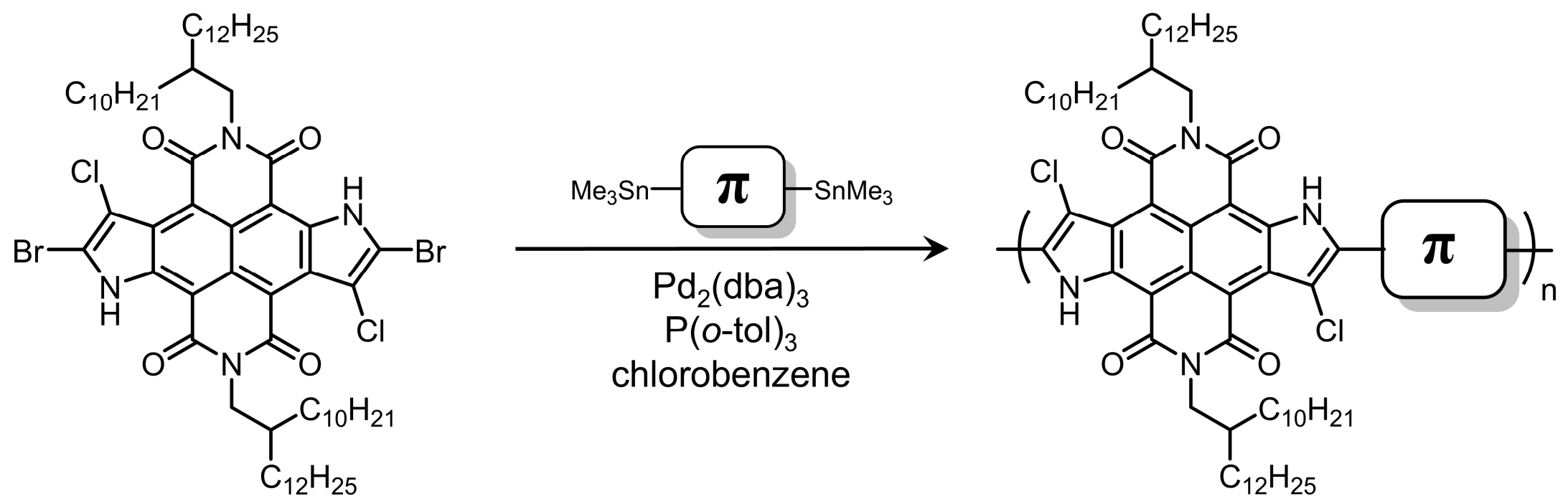

7

General Procedure VII (GP VII): Compound **7** (0.05 mmol, 62.2 mg), tin reagent (0.05 mmol), Pd_2dba_3 (2.5 mol%, 1.14 mg), and P(*o*-tol)$_3$ (10 mol%, 1.52 mg) were placed in a Schlenk tube under N_2 followed by the addition of 2.5 mL dry chlorobenzene. The mixture was degassed by three freeze-pump-thaw cycles. After 2 days, 2-(tributylstannyl)thiophene (50 μL) was added to the reaction mixture and stirred for 4 hours before the addition of 2-bromothiophene (100 μL). After another 4 hours, the reaction mixture was cooled to room temperature and added to 100 mL of methanol. The precipitate was collected and dried. The crude polymer was then purified by sequential Soxhlet extraction with acetone, hexane, dichloromethane, chloroform, and chlorobenzene. The final chloroform or chlorobenzene fraction was concentrated and precipitated into methanol. The resulting precipitate was filtrated, washed, and dried under vacuum to yield the product polymer.

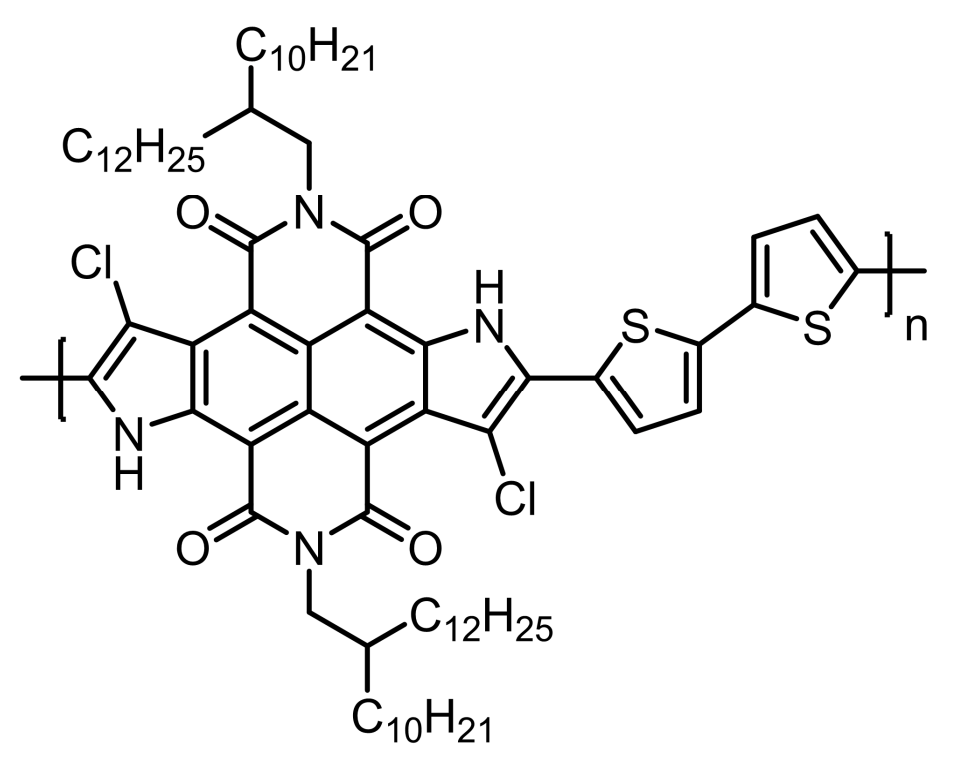

P-2T. GP VII was applied to compound **8** (1 eq) and 5,5'-bis(trimethylstannyl)-2,2'-bithiophene (1 eq, 0.05 mmol, 24.6 mg). Soxhlet extraction yielded **P-2T** as black solids (chloroform fraction, 52.5 mg, 84%). HT-GPC (150 °C, 1,2,4-trichlorobenzene): M_n = 14.8 kDa, M_w = 25.6 kDa, $Đ$ = 1.7.

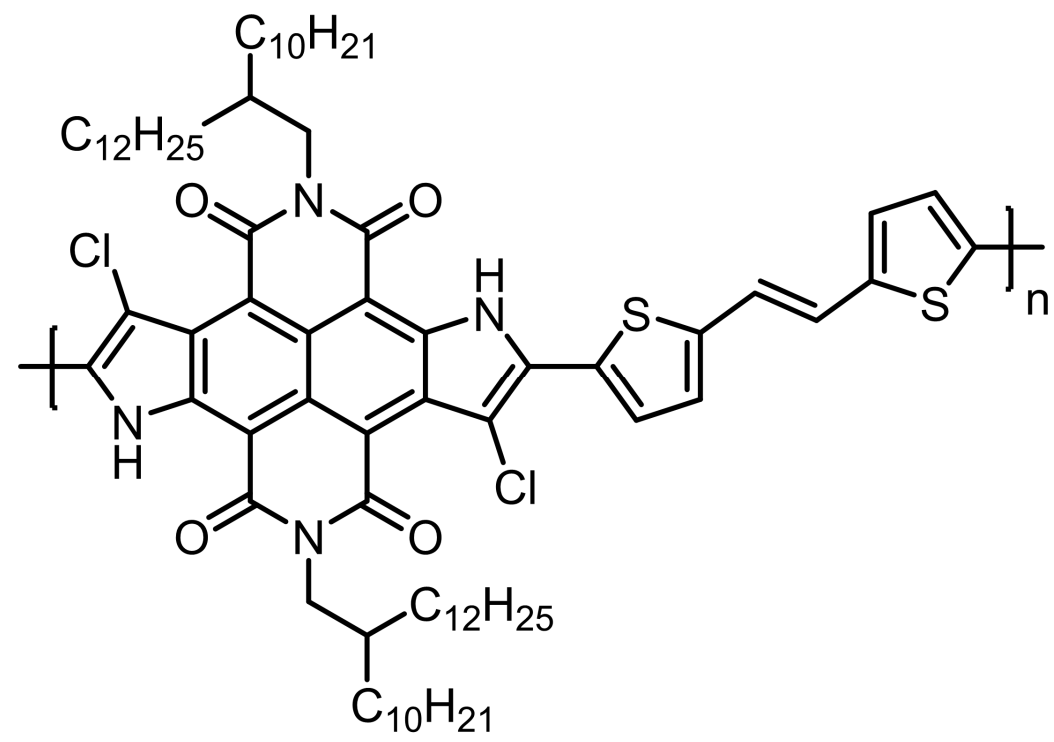

P-TVT. GP VII was applied to compound **8** (1 eq) and *trans*-1,2-bis[5-(trimethylstannyl)thiophen-2-yl]ethene (1 eq, 0.05 mmol, 25.9 mg). Soxhlet extraction yielded **P-2T** as black solids (chloroform fraction, 60.5 mg, 95%). HT-GPC (150 °C, 1,2,4-trichlorobenzene): M_n = 19.9 kDa, M_w = 42.8 kDa, $Đ$ = 2.1.

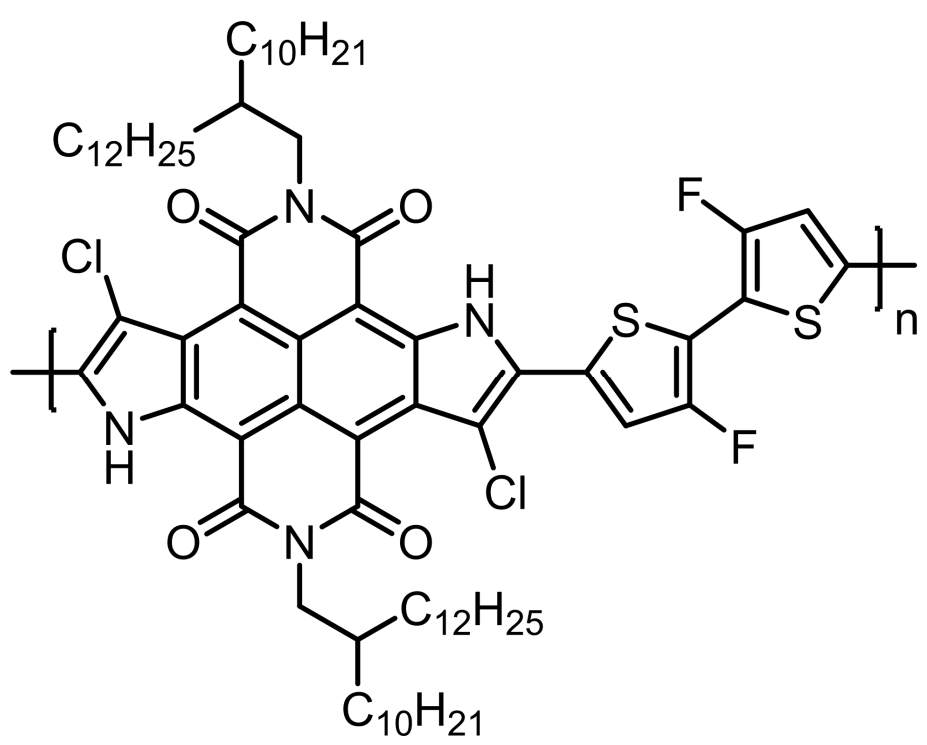

P-2F2T. GP VII was applied to compound **8** (1 eq) and (3,3'-difluoro-[2,2'-bithiophene]-5,5'-diyl)bis(trimethylstannane) (1 eq, 0.05 mmol, 26.4 mg). Soxhlet extraction yielded **P-2F2T** as black solids (chloroform fraction, 25.8 mg, 40%; chlorobenzene fraction, 35.8 mg, 56%). HT-GPC (150 °C, 1,2,4-trichlorobenzene): chloroform fraction, M_n = 19.7 kDa, M_w = 36.3 kDa, $Đ$ = 1.8; chlorobenzene fraction, M_n = 27.5 kDa, M_w = 65.0 kDa, $Đ$ = 2.4. Chlorobenzene fraction was used for characterization of optoelectronic properties and for device fabrication.

3. X-ray Crystallography Data

Crystal data and refinement of **5b**: Thermal ellipsoids were drawn at the 50% probability level. Crystals suitable for X-ray analysis were obtained via slow evaporation of methanol/dichloromethane solution of **5b**.

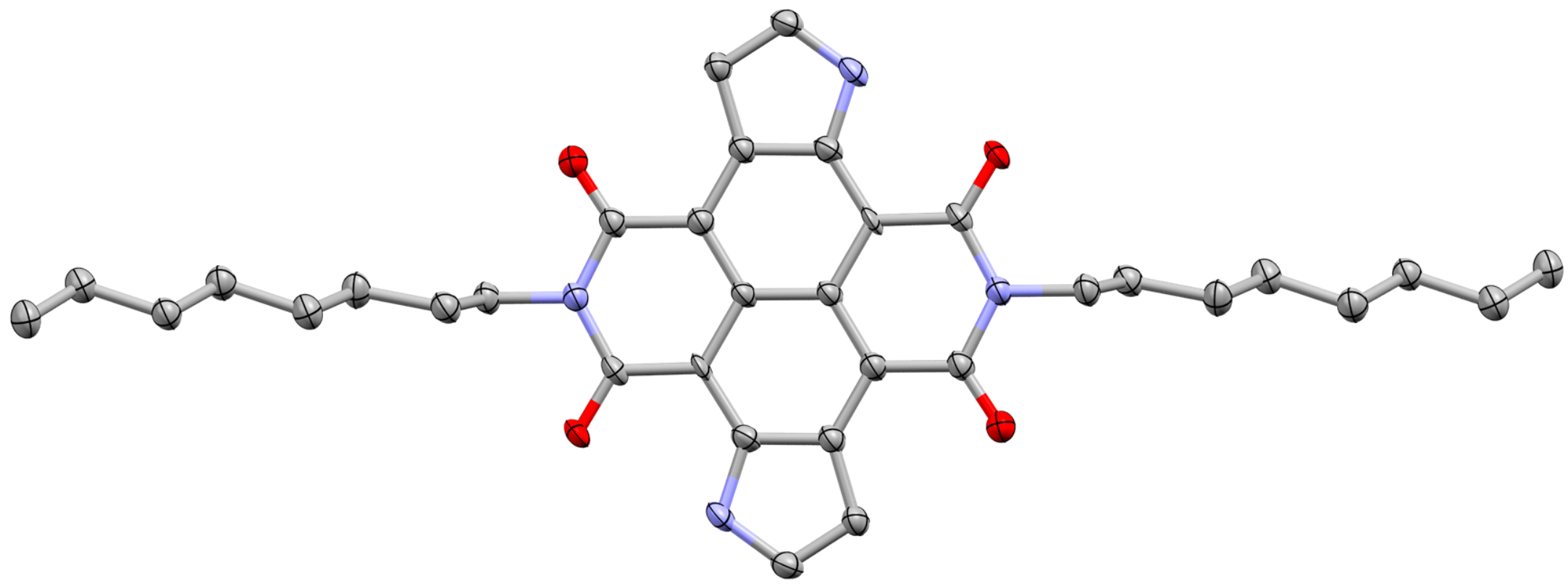

Empirical formula	$C_{34}H_{40}N_4O_4$	
Formula weight	568.70	
Temperature	100 K	
Wavelength	1.54184 Å	
Crystal system	Monoclinic	
Space group	*C2/c*	
Unit cell dimensions	a = 30.2202(13) Å	α = 90°
	b = 4.6449(2) Å	β = 92.306(4)°
	c = 20.0835(8) Å	γ = 90°
Volume	2816.8(2) $Å^3$	
Z	4	
Absorption coefficient	0.709 mm^{-1}	
F_{000}	1216	
Density(calculated)	1.341 g/cm^3	
Crystal size	0.23 × 0.05 × 0.04 mm	
Crystal shape	block	
Crystal color	purple	
θ range for data collection	2.91–72.32°	
Reflections collected	20054	
Independent reflections [R_{int}]	4271 [0.0797]	
Index ranges (h, k, l)	-36…36, -5…5, -24…24	
Absorption correction	Multi-scan	
Transmission factors: max, min	0.974, 0.855	
Refinement method	Full-matrix least-squares on F^2	
Data/ restraint/ parameters	2783/ 0/ 198	
Goodness-of-fit on F^2	1.413	

4. CV curves

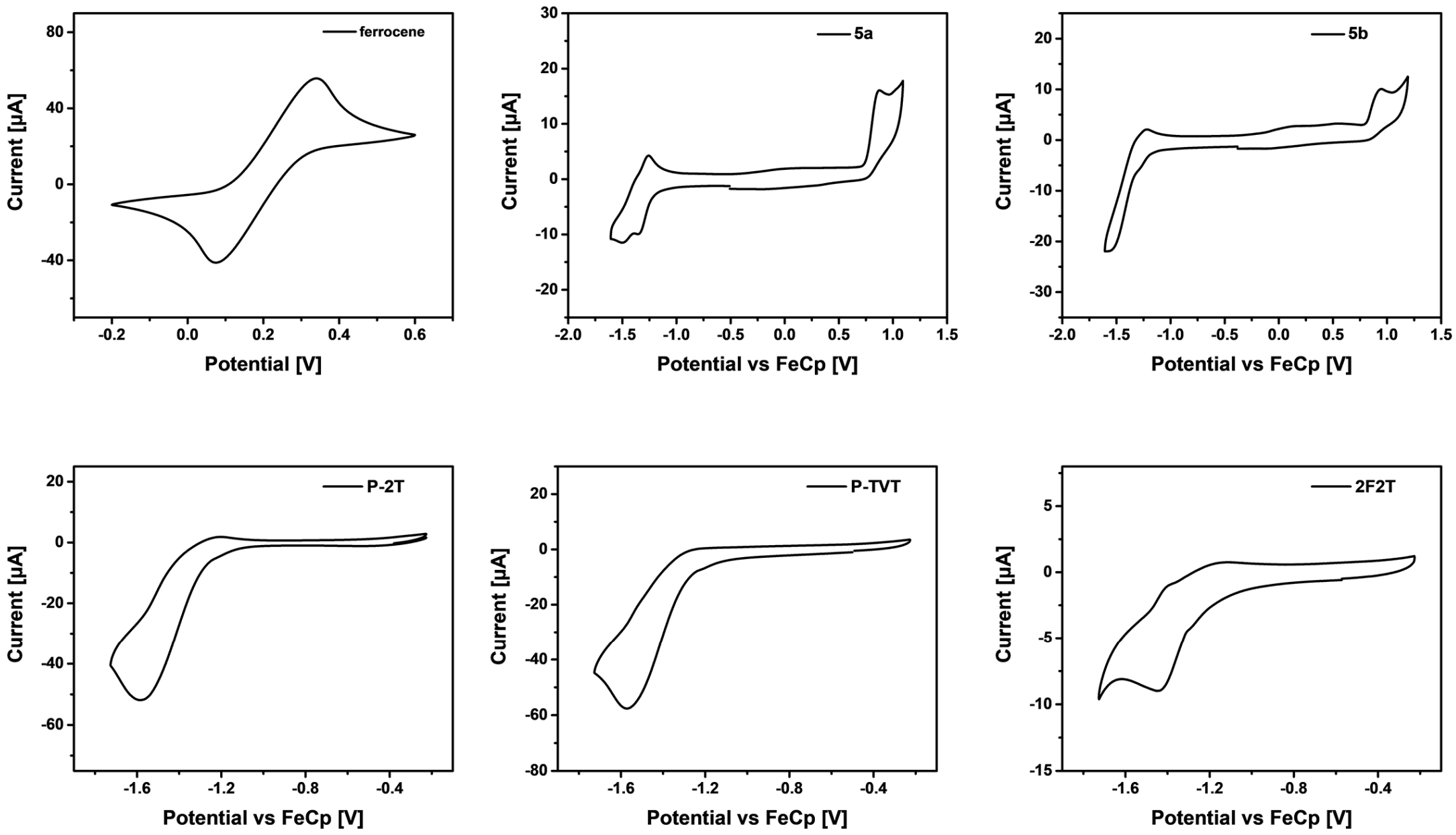

Figure S1. Cyclic voltammetry curves of **5a**, **5b** (in DCM solution) and **P-2T**, **P-TVT**, and **P-2F2T** (in acetonitrile). A three-electrode configuration was used (glassy carbon as working electrode, Pt as counter electrode, and Ag/AgCl as reference). The ferrocene/ferrocenium (Fc/Fc^+) couple was served as external reference. For the polymers, thin films were deposited onto the glassy carbon electrode.

5. Photoemission yield spectroscopy in air

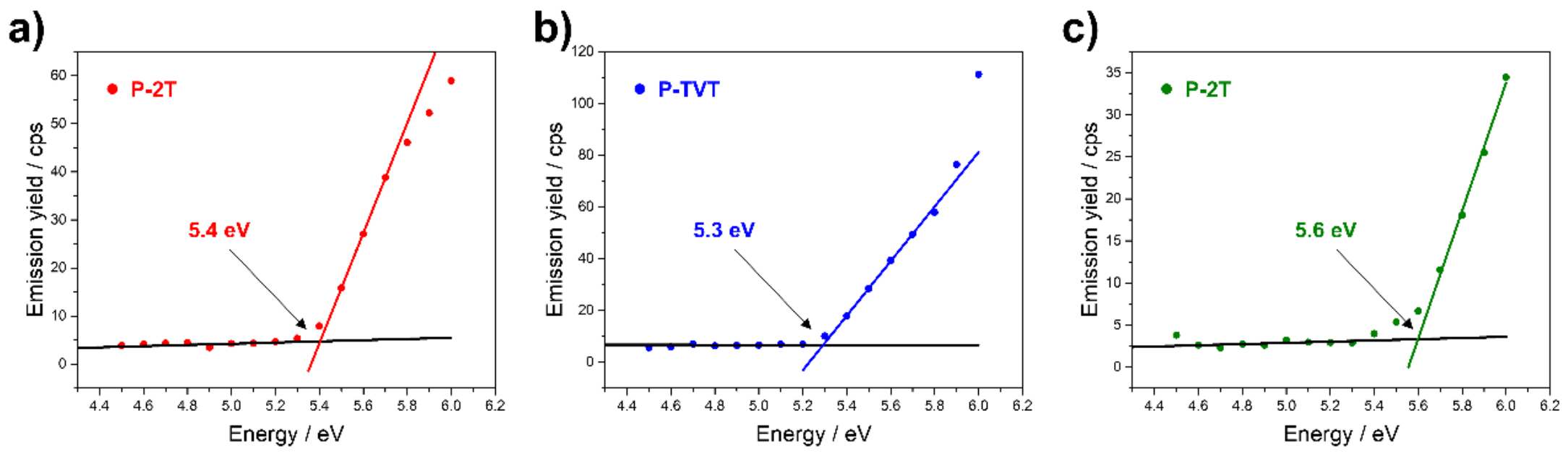

Figure S2. HOMO levels of **NDPI**-based polymers measured by photoemission yield spectroscopy in air. a) **P-2T**; b) **P-TVT**; c) **P-2F2T**.

6. Optical spectra

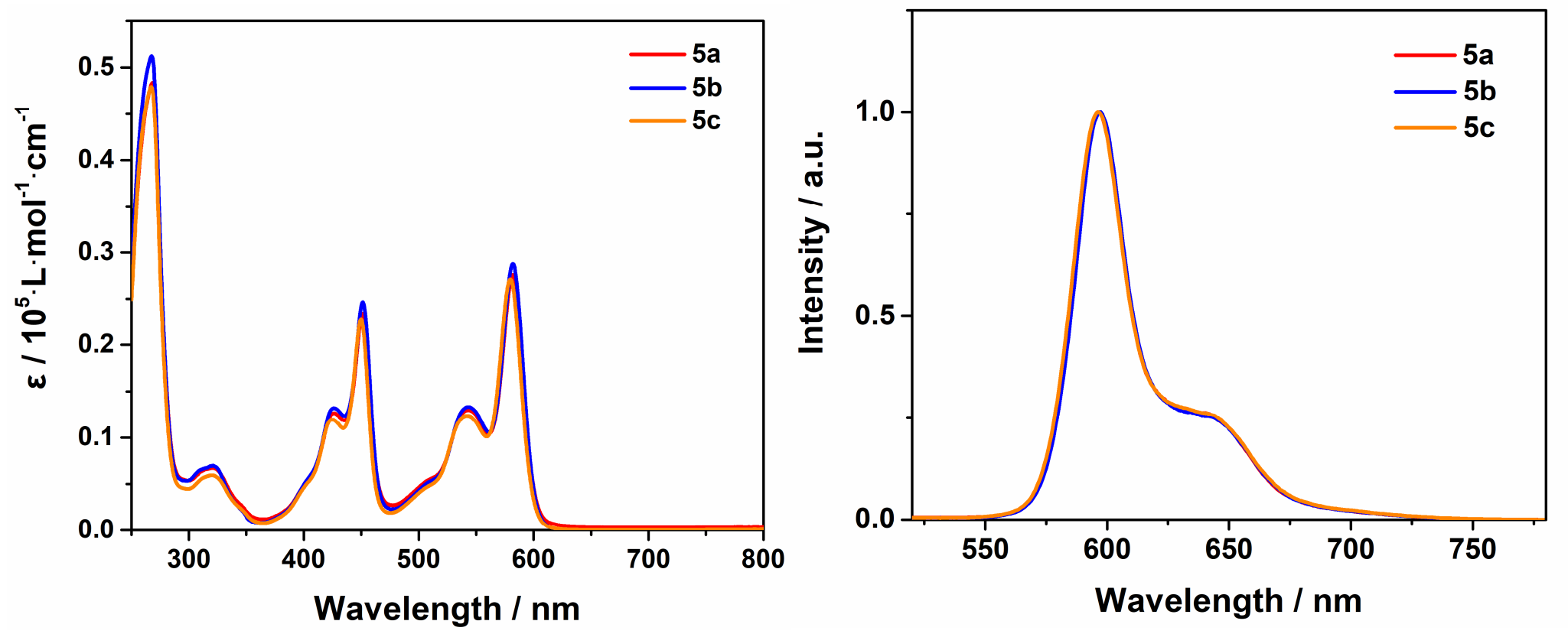

Figure S3. Absorption spectra (left) and photoluminescence (right) of **5a**, **5b**, and **5c** in DCM (10^{-5} M).

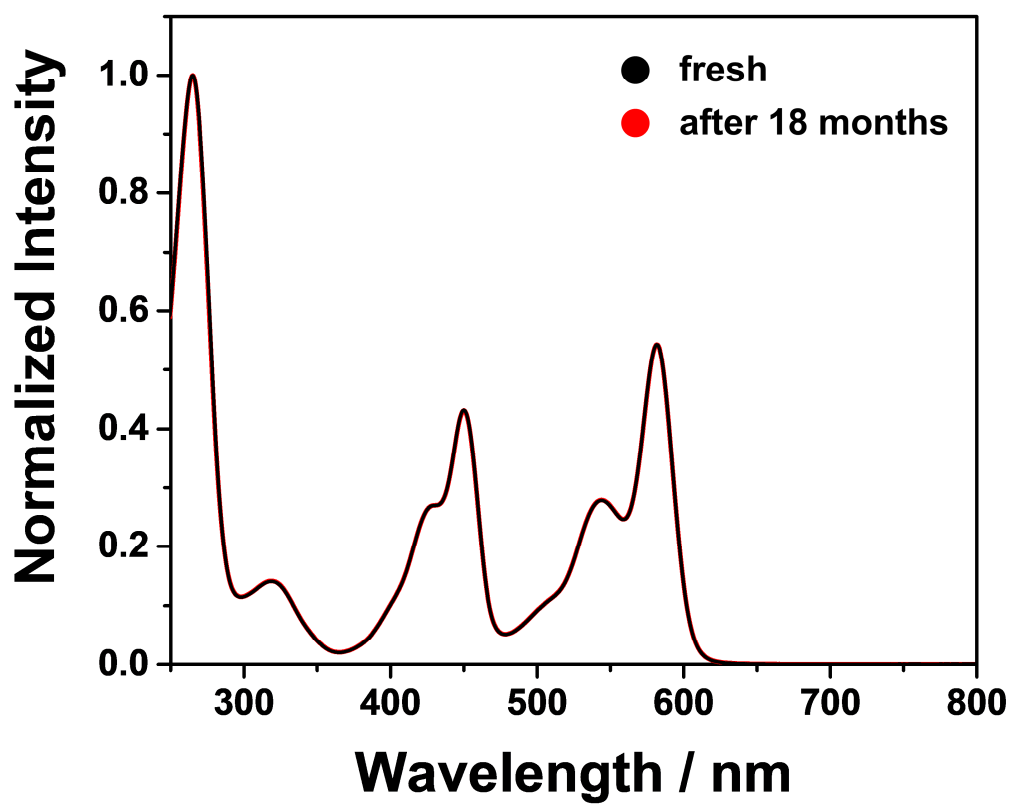

Figure S4. Absorption spectra of freshly prepared **5a** (black line) and **5a** stored under ambient conditions for 18 months (chloroform).

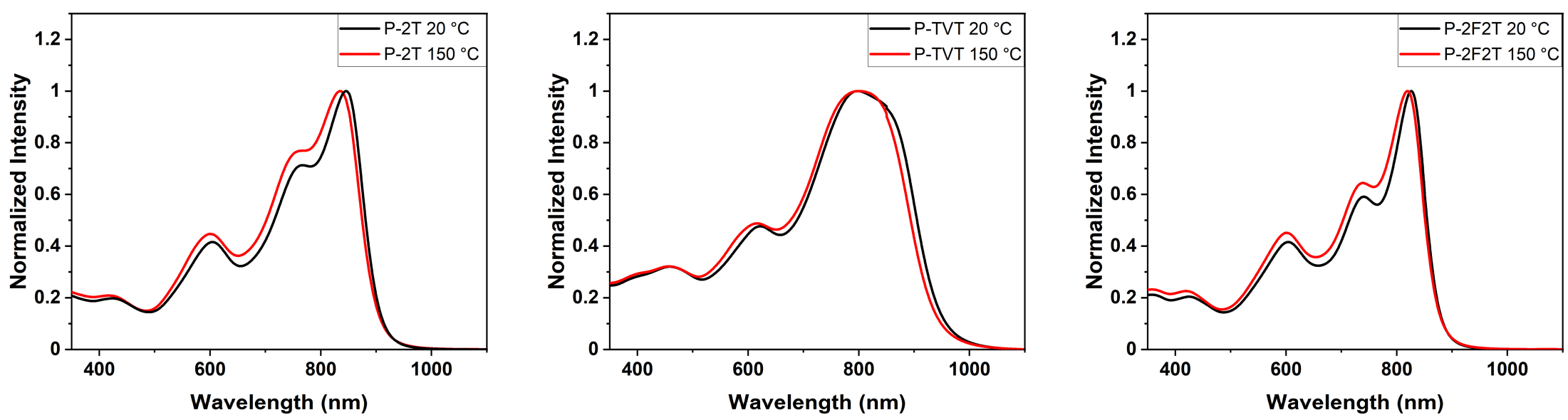

Figure S5. Variable temperature absorption spectra of a) **P-2T**, b) **P-TVT**, and c) **P-2F2T** in *o*-DCB (10^{-5} M).

7. Thermal Analysis

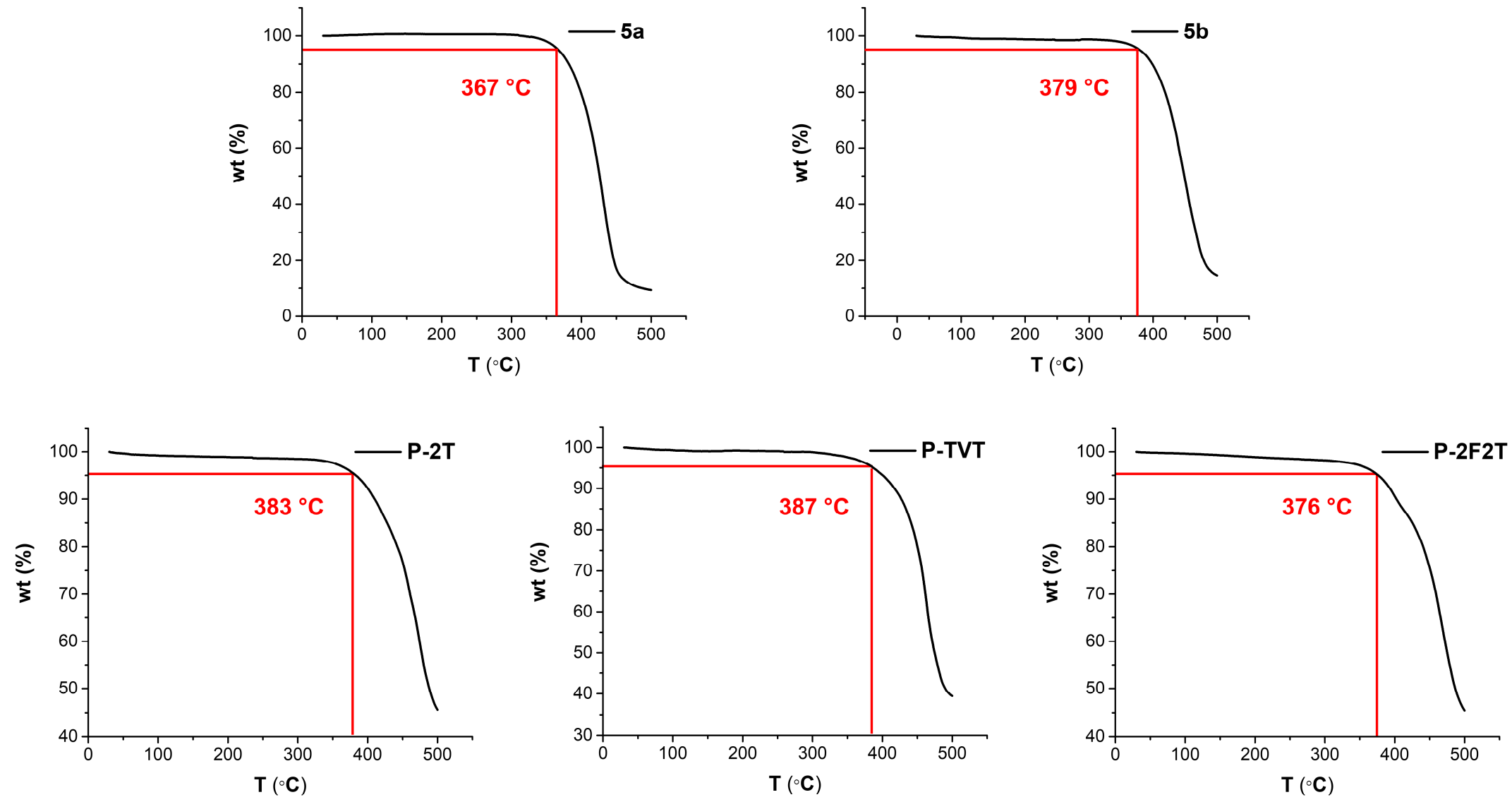

Figure S6. TGA curves of **5a**, **5b**, **P-2T**, **P-TVT**, and **P-2F2T**. T_d with 5% weight loss is marked with red lines.

8. High-Temperature GPC

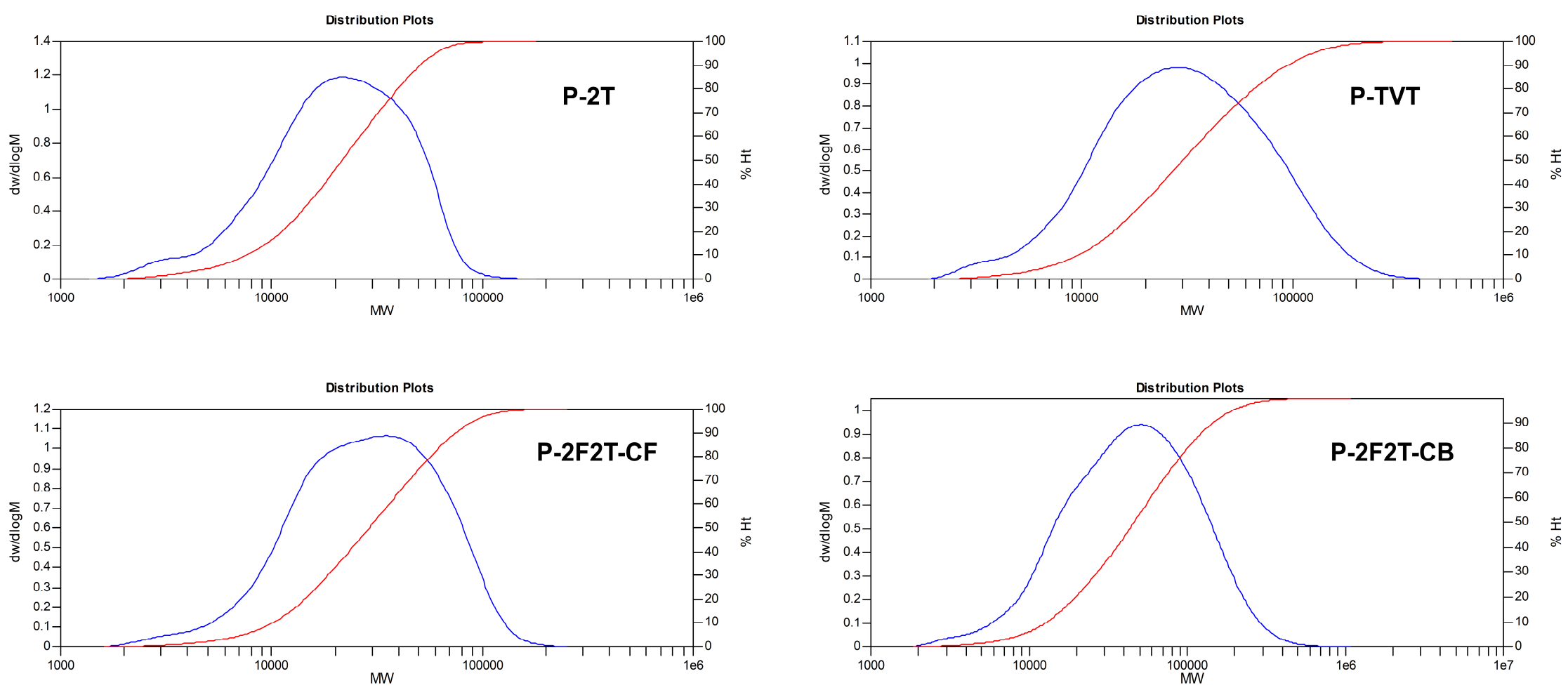

Figure S7. Polymer molecular weight distribution of **P-2T**, **P-TVT**, and **P-2F2T** (CF and CB fractions) determined by high-temperature gel permeation chromatography (HT-GPC) at 150 °C using 1,2,4-trichlorobenzene as the eluent.

9. Computational details

The optimization of molecular geometries and calculation of frontier molecular orbitals were carried out at the B3LYP-D3(BJ)/def2-TZVP level of theory. Calculations of ACID, NICS, and NMR were carried out at the B3LYP/pcSseg-2 level of theory. The DFT calculations were conducted using the Gaussian 16 software package.[2] The visualization of 2D-ICSS was conducted using py.Aroma.[3]

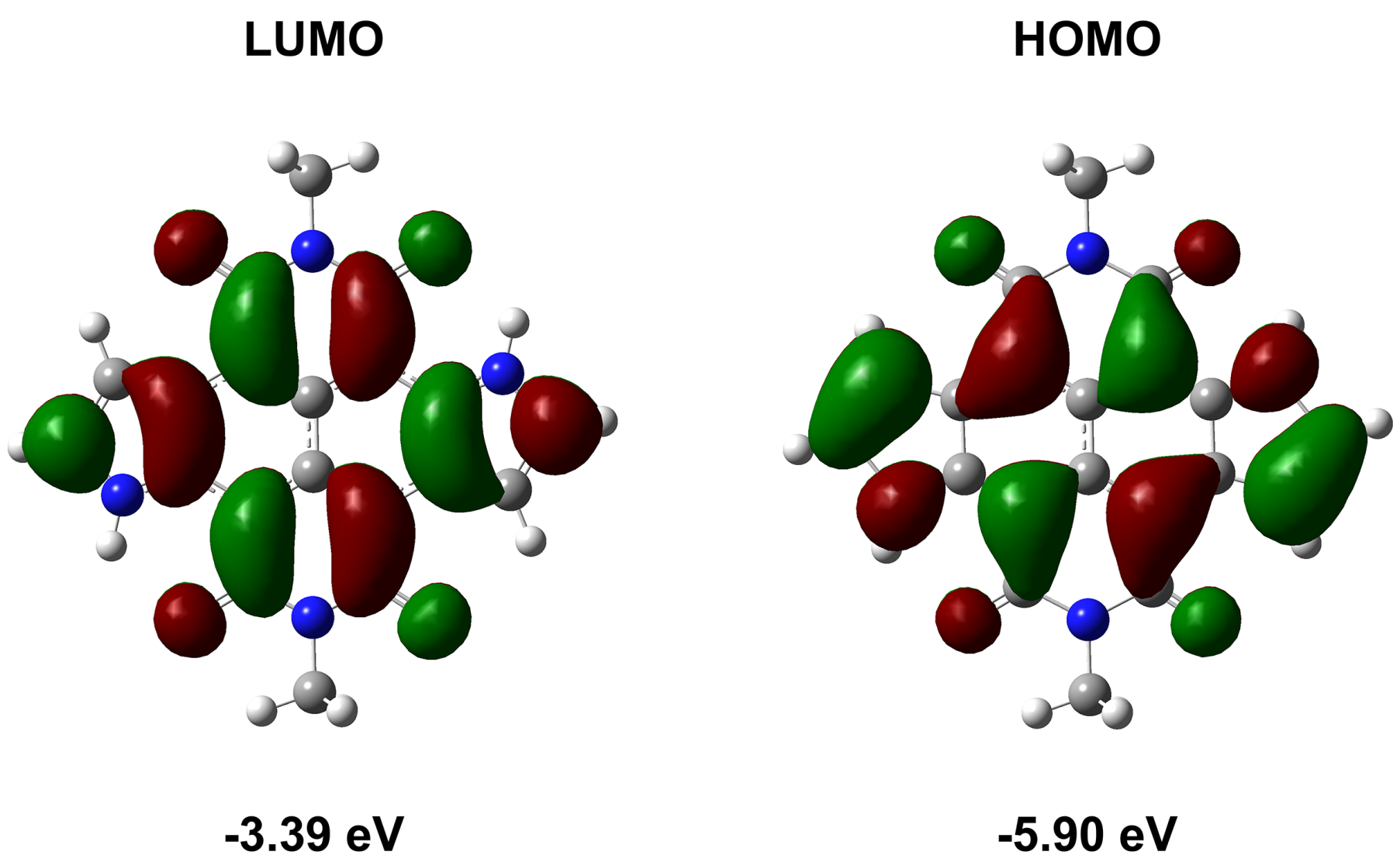

Figure S8. DFT-calculated frontier molecular orbitals of **NDPI-Me** (methyl group as the imide substituent), and their corresponding energy levels.

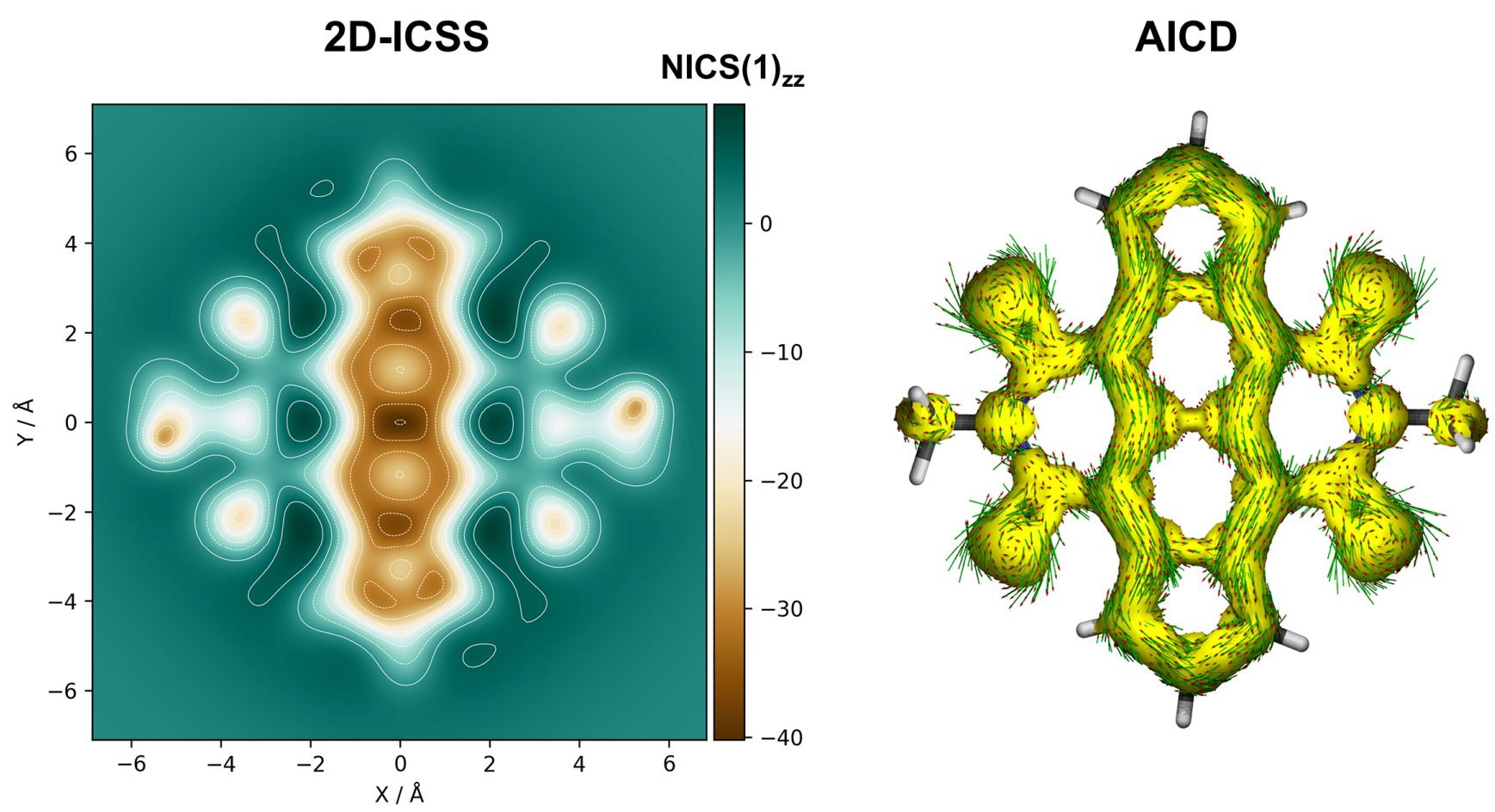

Figure S9. Left: 2D-ICSS of **NDPI-Me**; right: ACID of **NDPI-Me** with isovalue = 0.035.

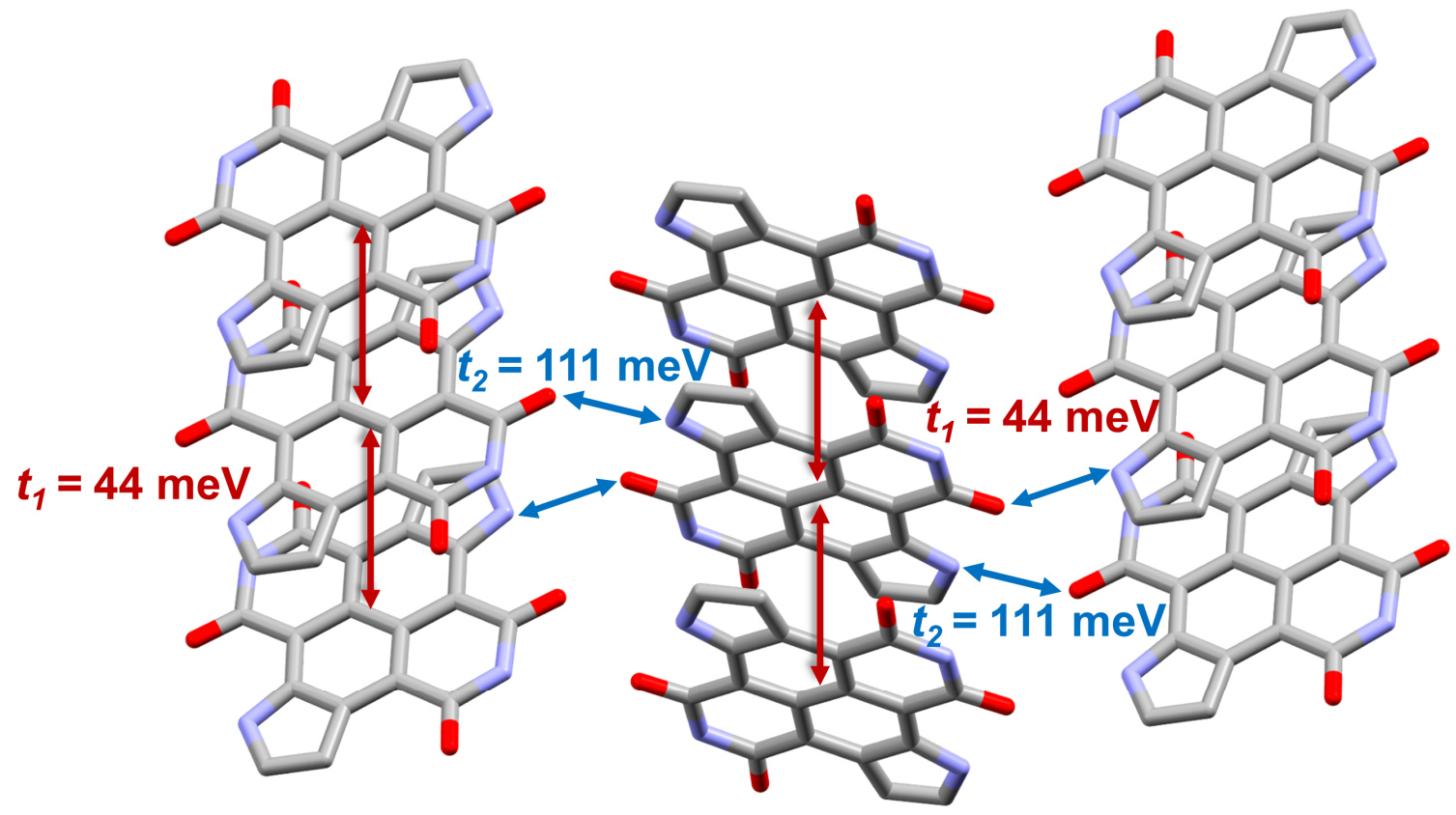

Figure S10. Electron transfer integrals of **5b** calculated by energy-splitting-in-dimer.[4] The dimer structures were taken from the crystal structure of **5b**. The calculations were carried out at PBEPBE/6-31G(d) level of theory. Side chains and hydrogen atoms are omitted for clarity.

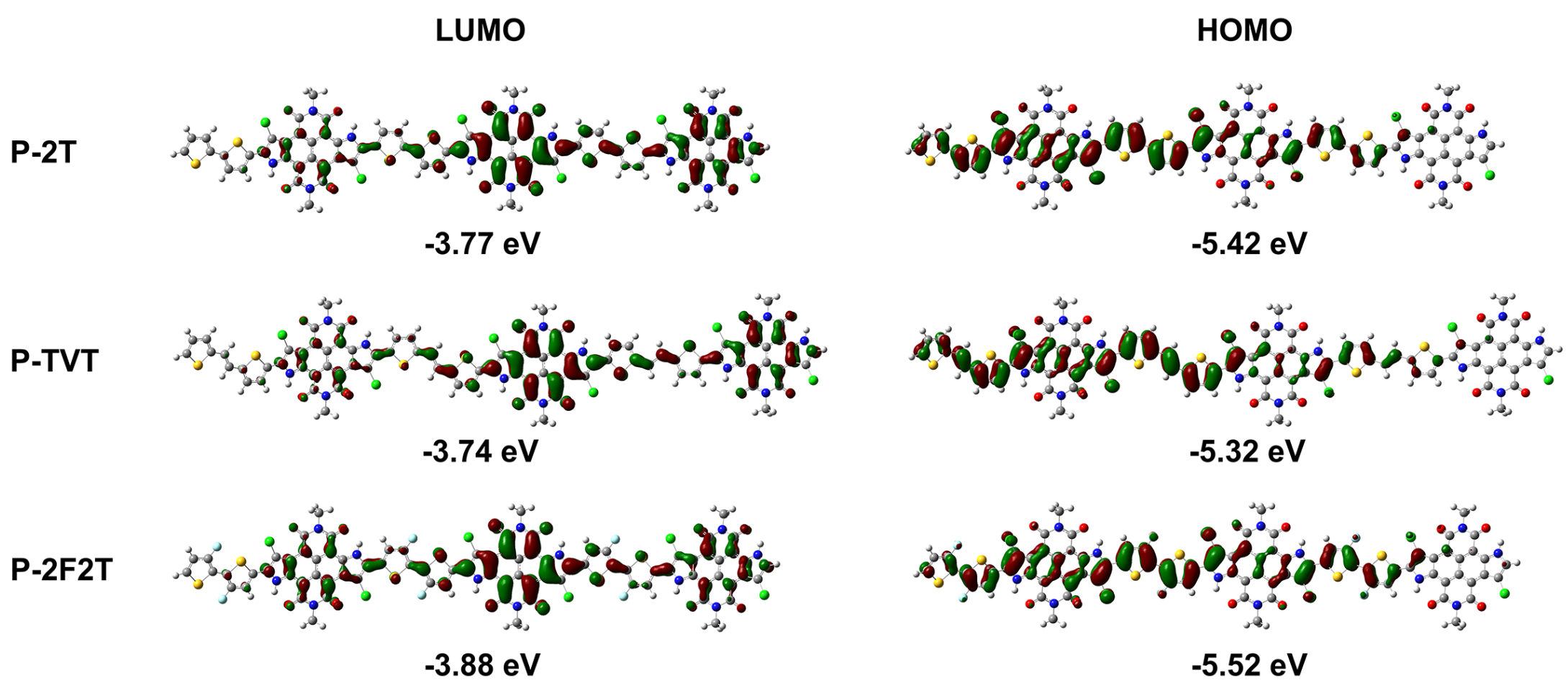

Figure S11. DFT-calculated frontier molecular orbitals of **NDPI**-based polymers (methyl group as the imide substituent), and their corresponding energy levels.

10. OFET Devices

Bottom-gate top-contact (BG/TC) organic thin-film transistors were fabricated on n^{++}-Si/SiO_2 substrates in which n^{++}-Si and SiO_2 were used as the gate electrode and gate dielectric, respectively. The substrates were cut into small squares of ca. 1.5 cm × 1.5 cm, which were cleaned with ultra-sonication in Milli-Q water, acetone and isopropanol for 5 minutes, and then dried with a stream of N_2 gas, and finally treated with oxygen plasma for 2 minutes. Octadecyltrimethoxysilane (ODTS)[5] and 12-cyclohexyldodecyl-phosphonic acid (CDPA)[6] modifications were conducted following the reported procedures.

For the CDPA modification, an $Al(NO_3)_3 \cdot 9H_2O$ solution (0.1 mol/L in ethanol) was spin-coated onto the Si/SiO_2 substrate at 5000 rpm for 40 seconds. The resulting film was baked at 300 °C for 30 minutes to achieve complete decomposition of nitrate and a high degree of dehydration. To form CDPA SAMs, the Si/SiO_2/AlO_x substrate was soaked in a 0.3 mM solution of CDPA in isopropanol at room temperature for 12 hours, and then rinsed with isopropanol and dried with a stream of nitrogen.

Thin films of the polymers were deposited on the treated substrate by spin-coating the polymer solutions (3 mg mL^{-1} in chlorobenzene, 3000 rpm for 45 seconds) inside a nitrogen-filled glovebox followed by thermal annealing. The optimal thermal annealing condition was determined to be 300 °C for 30 min inside the glovebox. After the thermal annealing, ca. 50 nm thick gold was deposited as the source and drain contacts using a shadow mask. The devices had a channel length (L) of 100 μm and a channel width (W) of 1 mm. The thin-film transistor performances were measured under vacuum using a Keithley 4200 parameter analyzer on a probe stage. The carrier mobilities, μ, were calculated from the data according to the following equation: $I_{DS} = (W/2L)C_i\mu(V_G - V_{th})^2$, where I_{DS} is the drain current in the saturated regime, W and L are the semiconductor channel width and length, respectively. C_i (13.7 nF cm^{-2} for ODTS, 11.5 nF cm^{-2} for CDPA) is the capacitance per unit area of the gate dielectric layer, and V_G and V_{th} are the gate voltage and threshold voltage, respectively. V_G-V_{th} of the devices was determined from the square root values of I_{DS}.

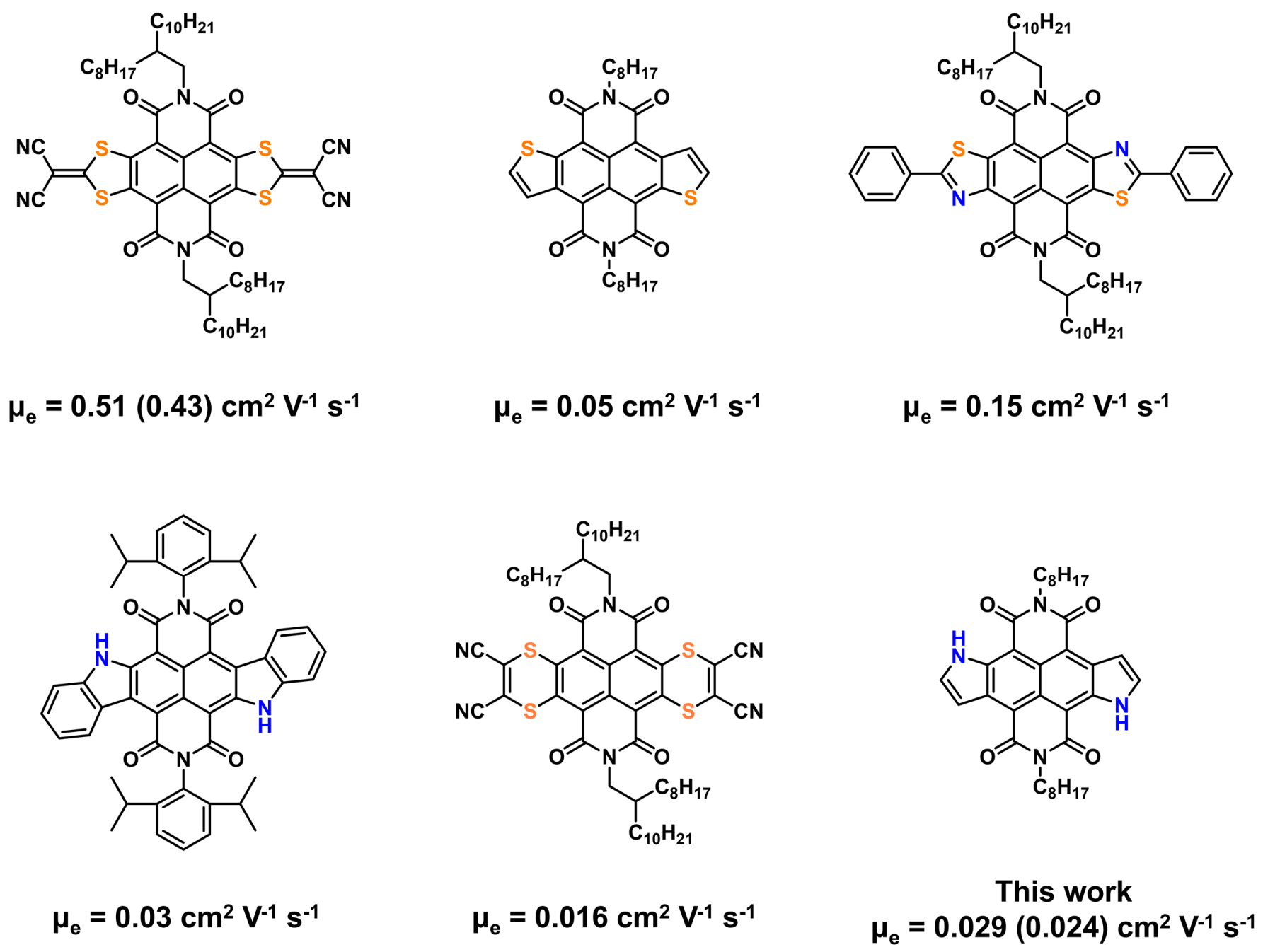

Figure S12. Mobilities of some heterocycle-fused NDIs.[1, 7] The value in the parenthesis is the average mobility.

11. GIWAXS Analysis

Table S1. Summary of lamellar (100, 200) and π–π stacking (010) parameters extracted from the 2D-GIWAXS patterns of NDPI-based polymer films. Values include *d*-spacings and their corresponding crystal coherence lengths (CCLs)

Polymer	$d_{(100)}$ (Å)	$CCL_{(100)}$ (Å)	$d_{(200)}$ (Å)	$CCL_{(200)}$ (Å)	$d_{(010)}$ (Å)	$CCL_{(010)}$ (Å)
**P-2T**	26.0	147.1	13.0	113.7	3.4	36.7
**P-TVT**	25.3	125.0	12.5	106.1	4.1	24.2
**P-2F2T**	28.7	73.1	13.5	67.2	Low intensity	/
**P-2F2T** (CDPA)	25.6	87.2	13.1	69.7	Low intensity	/

12. NMR spectra

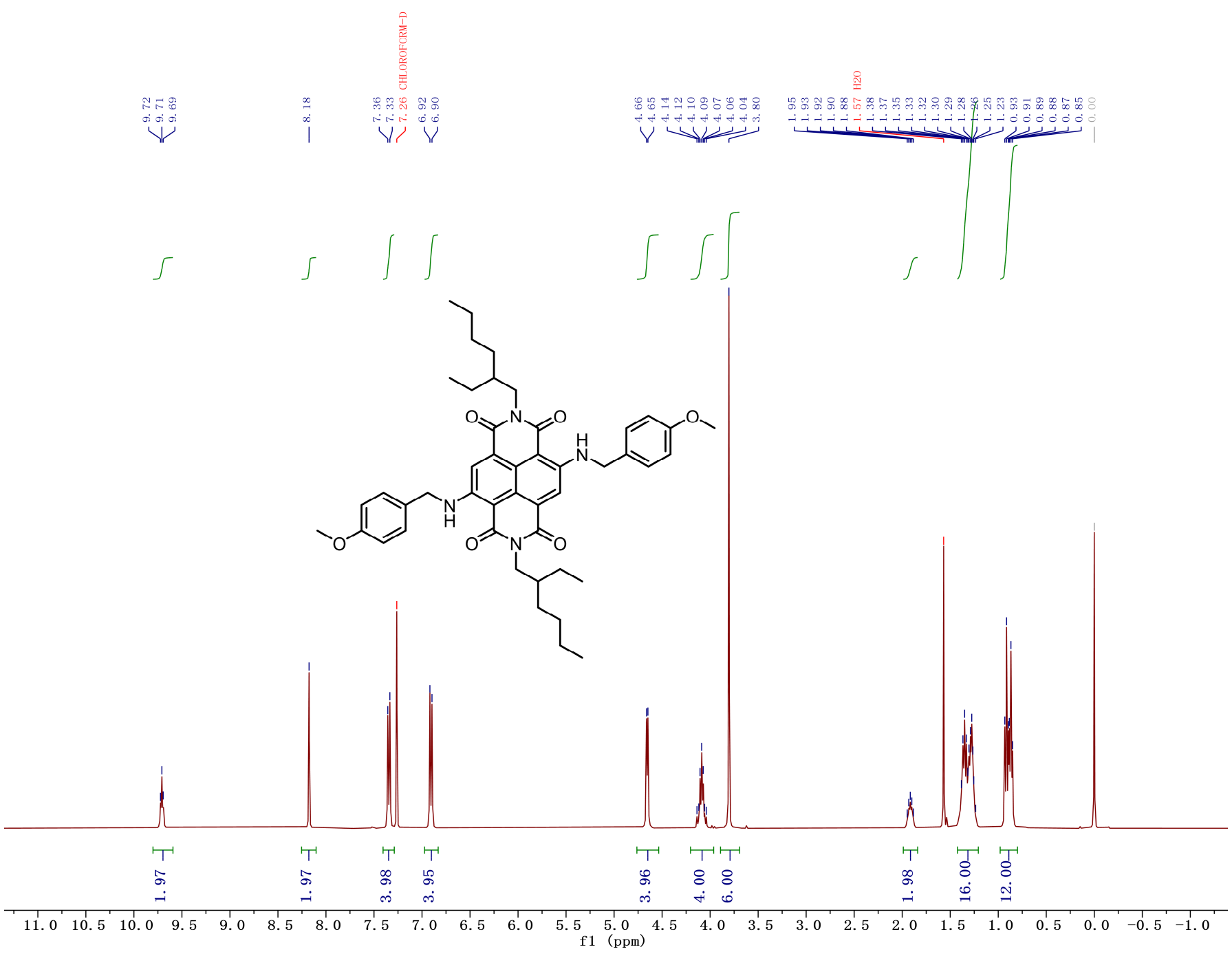

Figure S13. ^{1}H NMR spectrum (400 MHz) of **NDI-2NHPMB-EH** in $CDCl_3$.

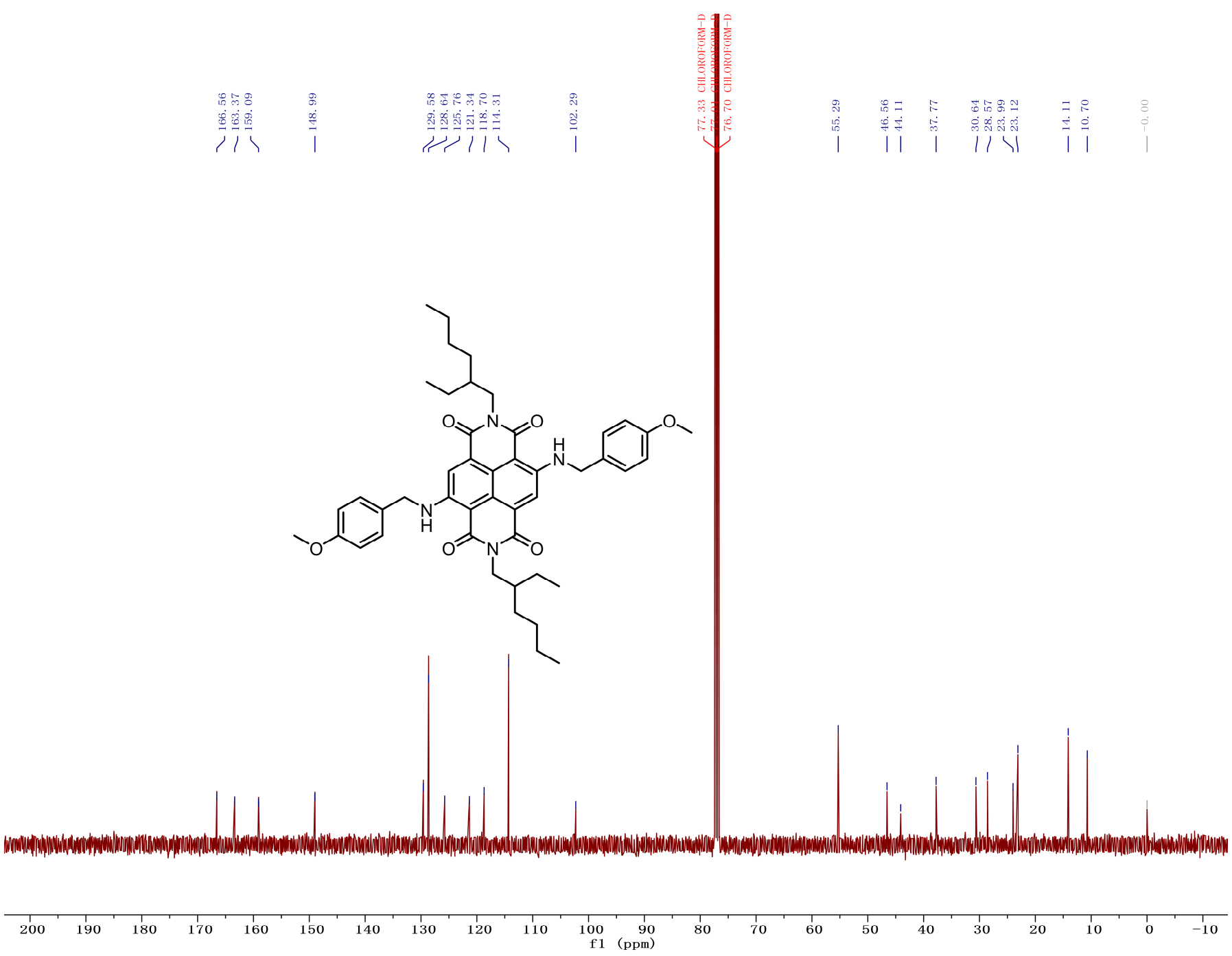

Figure S14. $^{13}C\{^{1}H\}$ NMR spectrum (100 MHz) of **NDI-2NHPMB-EH** in $CDCl_3$.

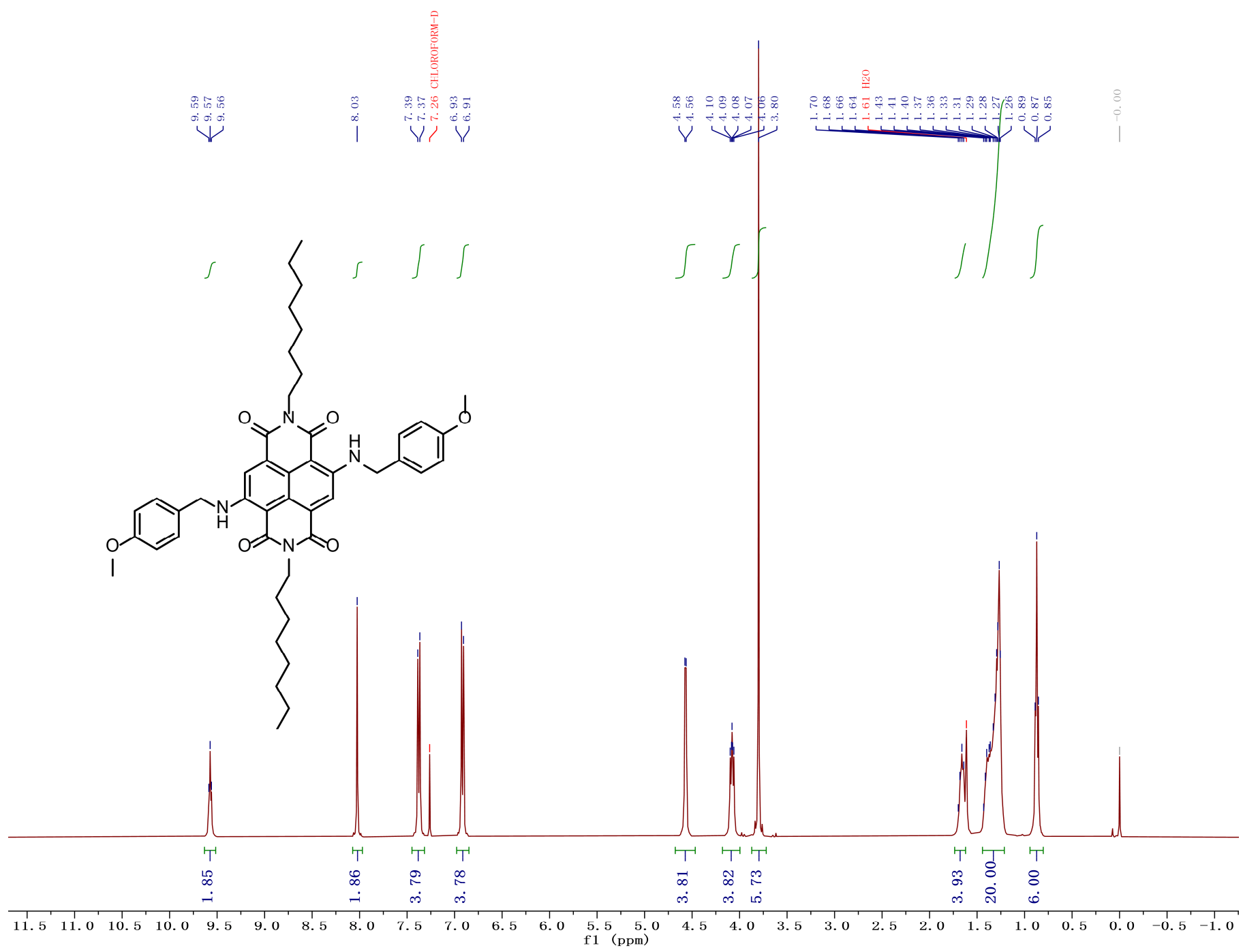

Figure S15. ^{1}H NMR spectrum (400 MHz) of **NDI-2NHPMB-Oct** in $CDCl_3$.

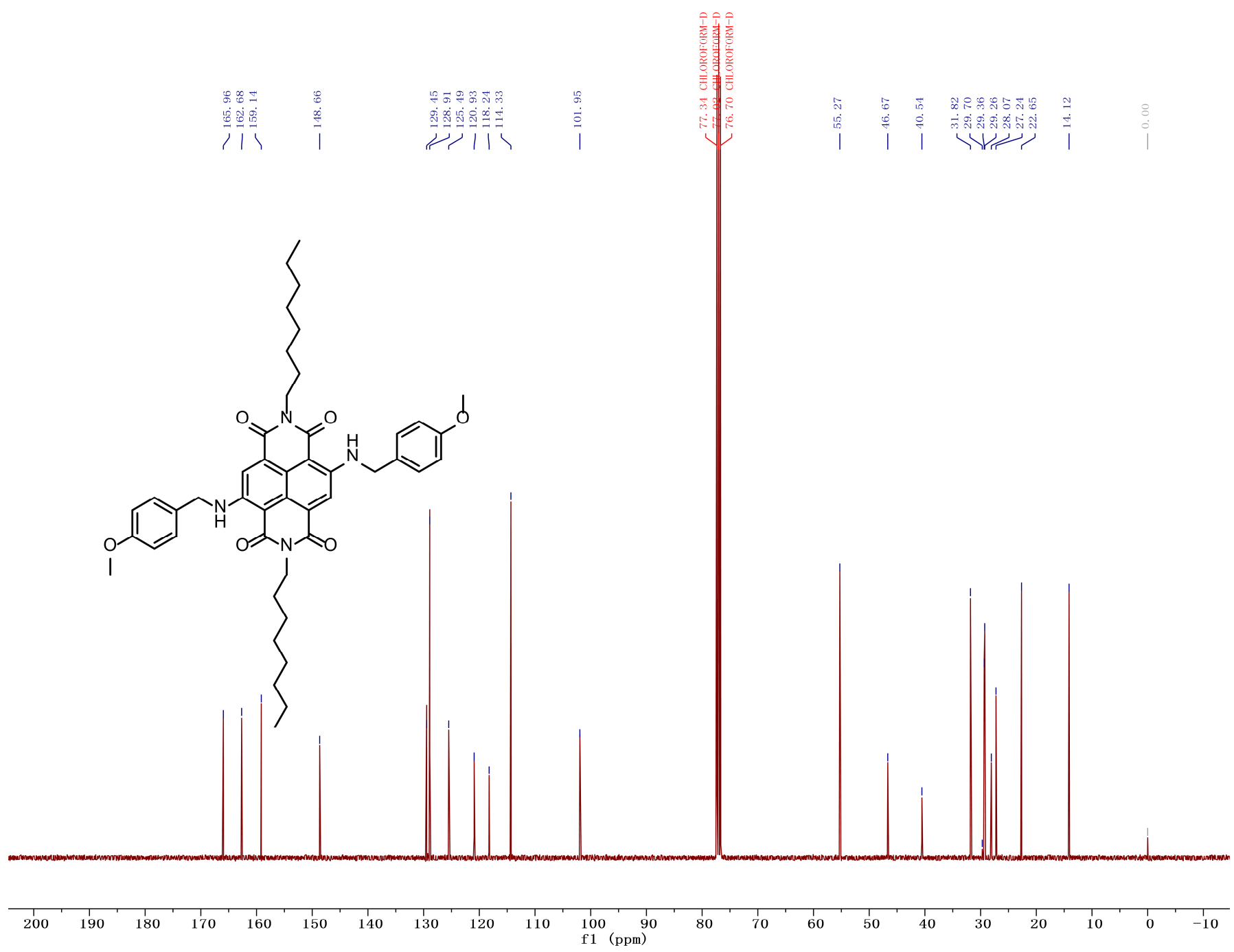

Figure S16. $^{13}C\{^{1}H\}$ NMR spectrum (100 MHz) of **NDI-2NHPMB-Oct** in $CDCl_3$.

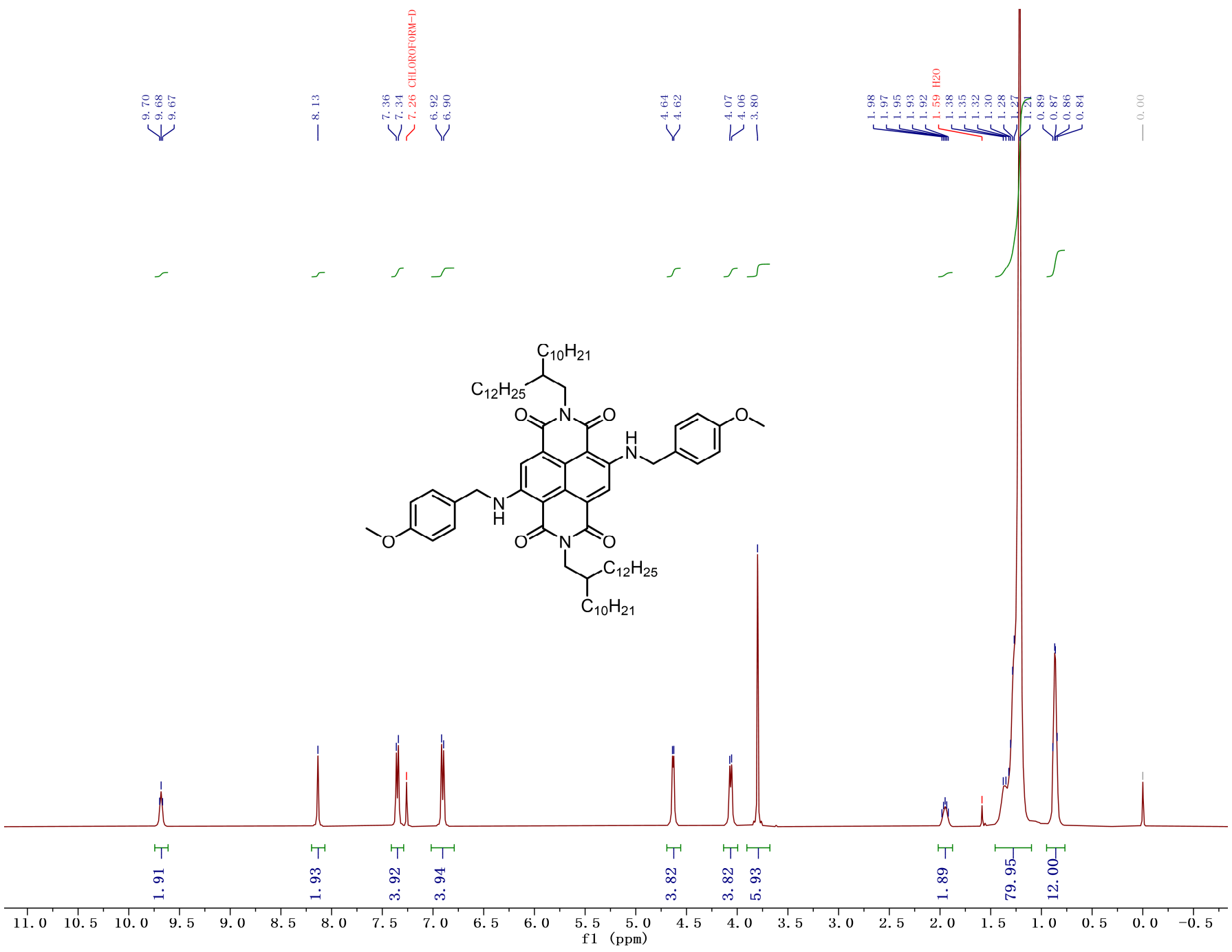

Figure S17. ^{1}H NMR spectrum (400 MHz) of **NDI-2NHPMB-DT** in $CDCl_3$.

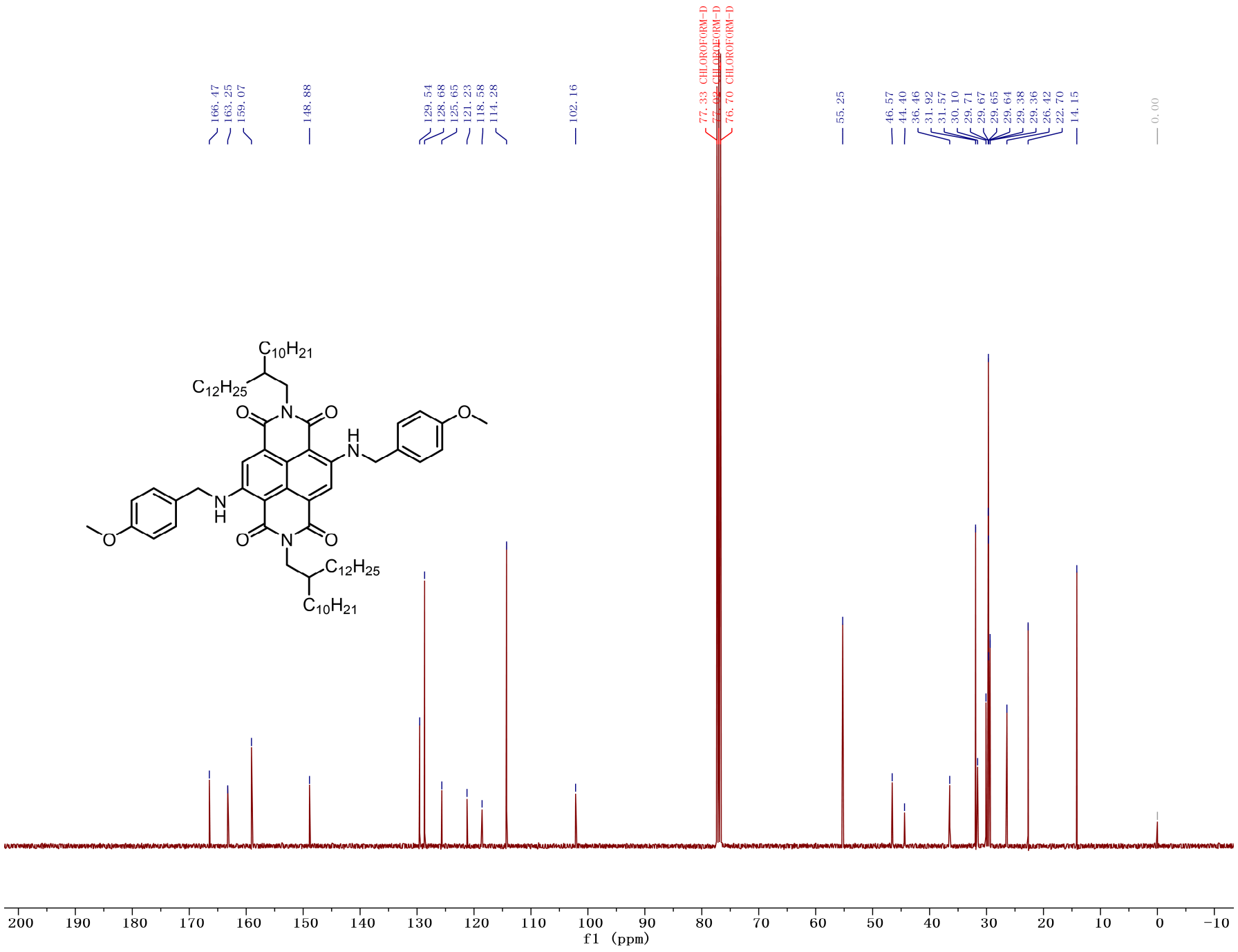

Figure S18. $^{13}C\{^{1}H\}$ NMR spectrum (100 MHz) of **NDI-2NHPMB-DT** in $CDCl_3$.

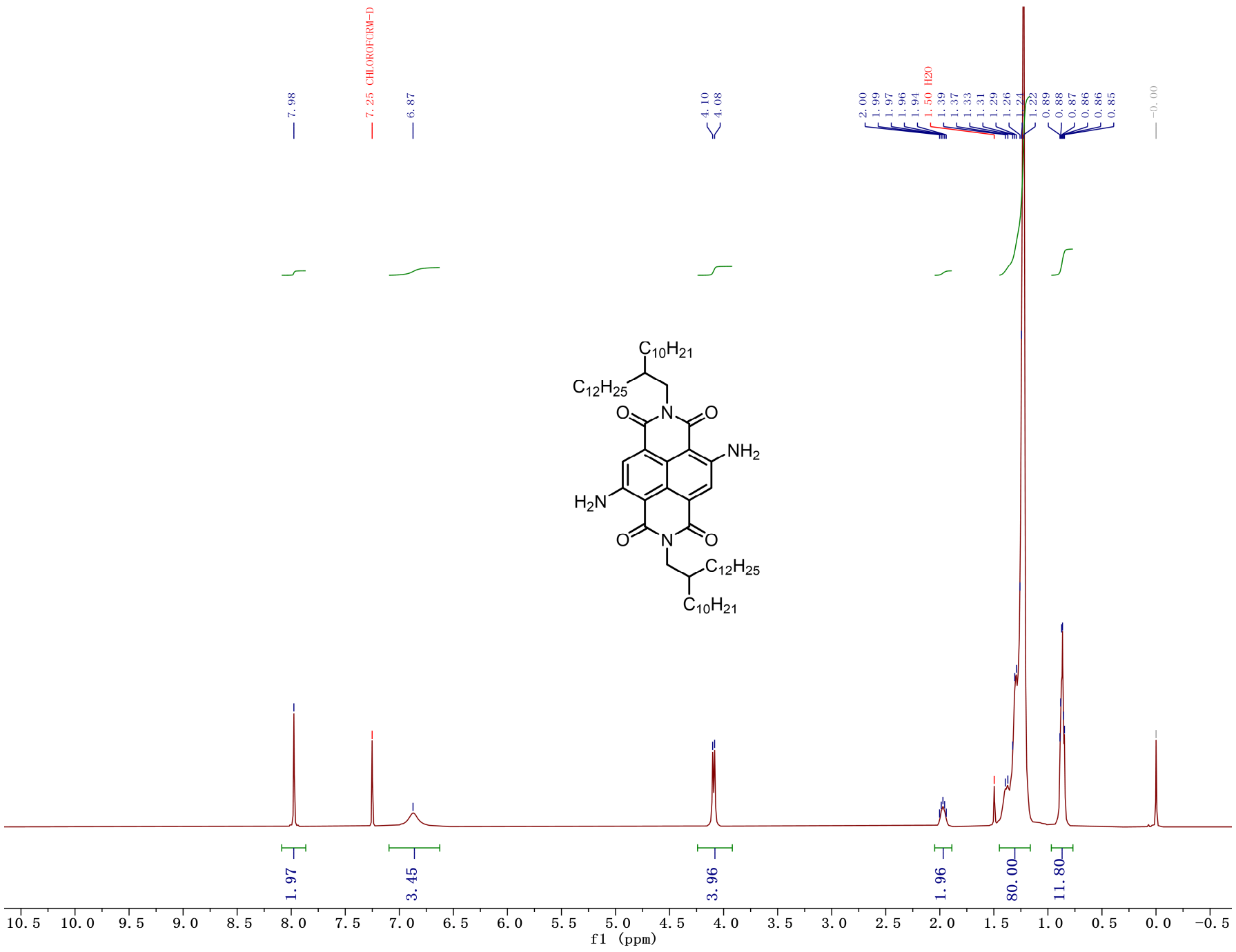

Figure S19. ^{1}H NMR spectrum (400 MHz) of **1c** in $CDCl_3$.

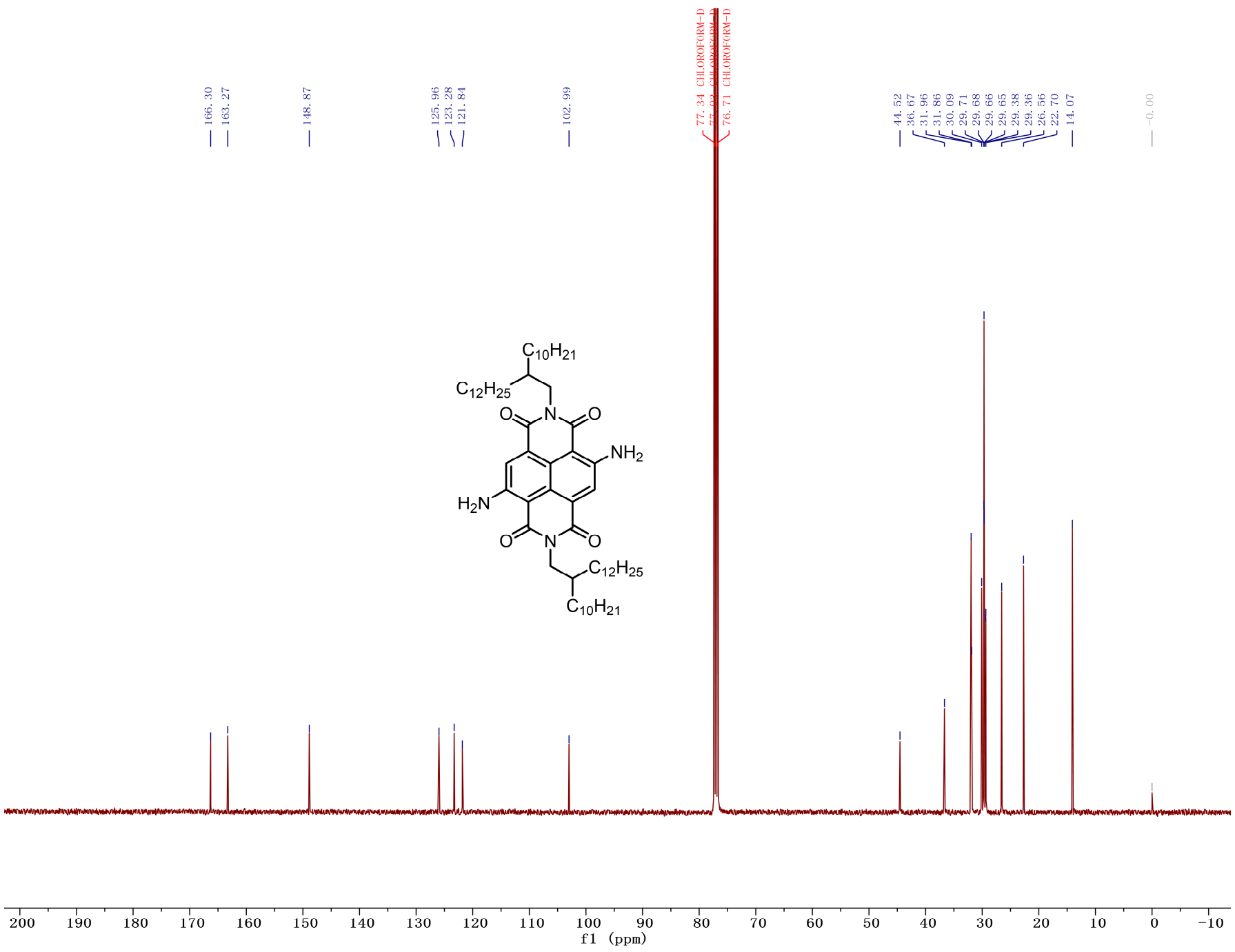

Figure S20. $^{13}C\{^{1}H\}$ NMR spectrum (100 MHz) of **1c** in $CDCl_3$.

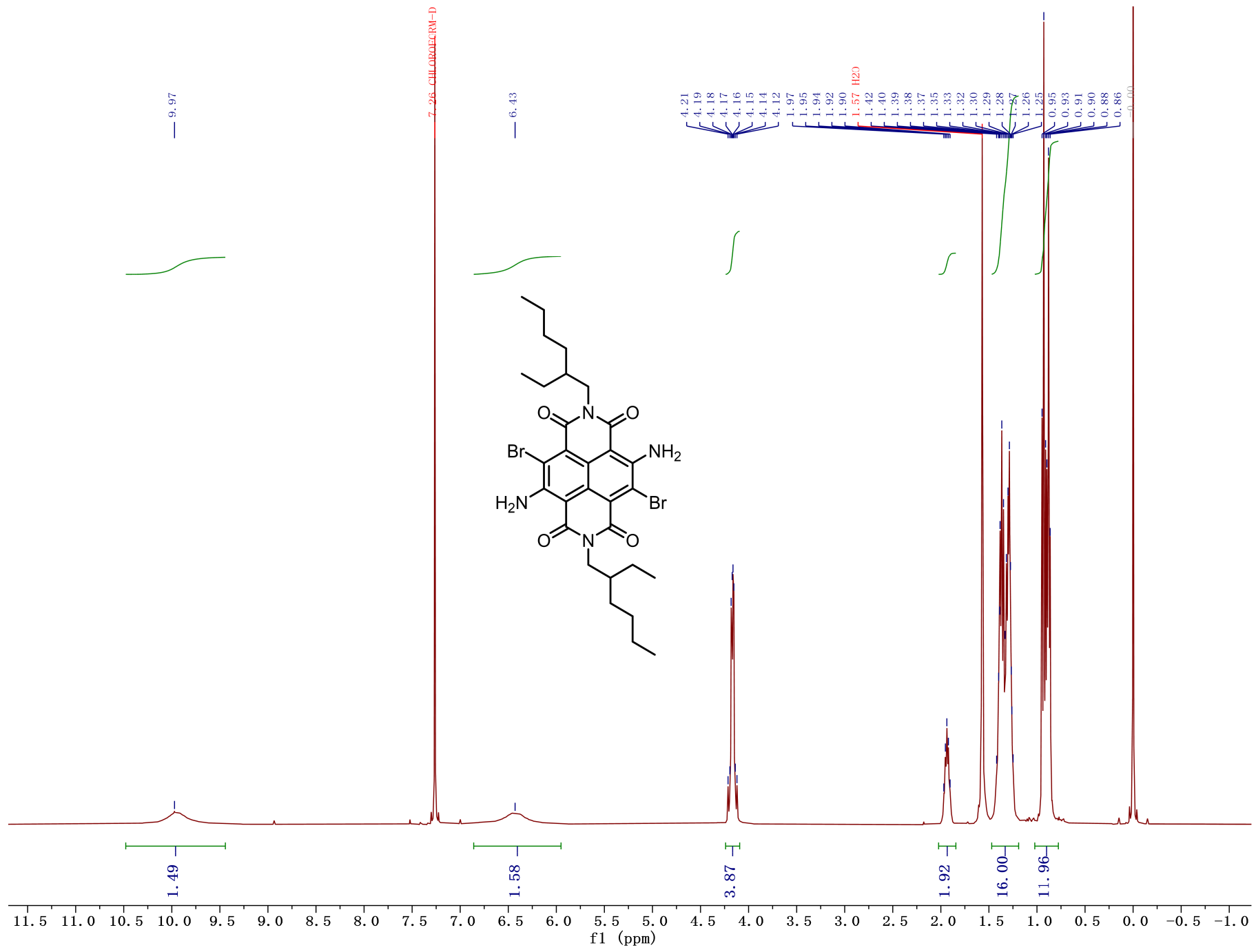

Figure S21. ^{1}H NMR spectrum (400 MHz) of **2a** in $CDCl_3$.

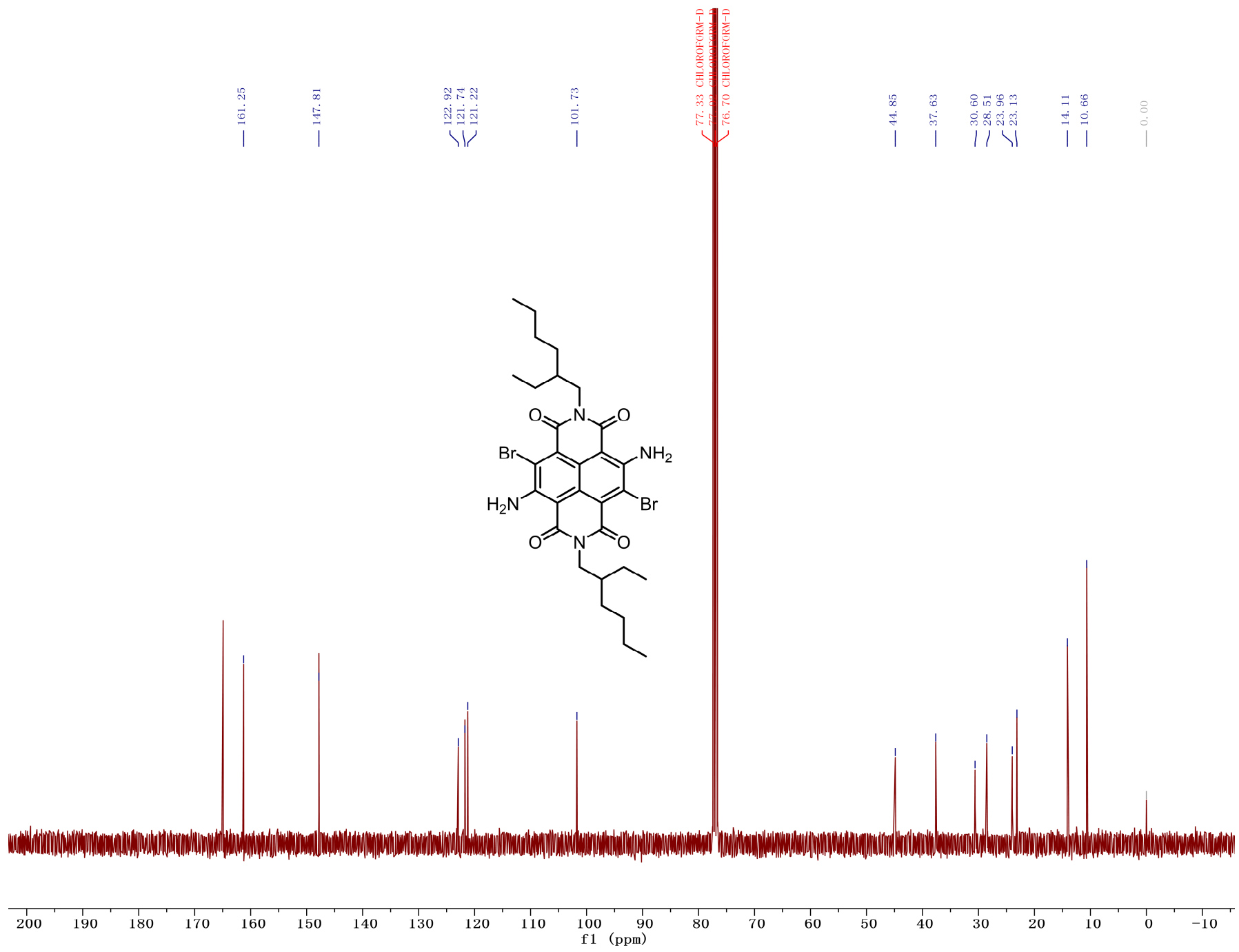

Figure S22. $^{13}C\{^{1}H\}$ NMR spectrum (100 MHz) of **2a** in $CDCl_3$.

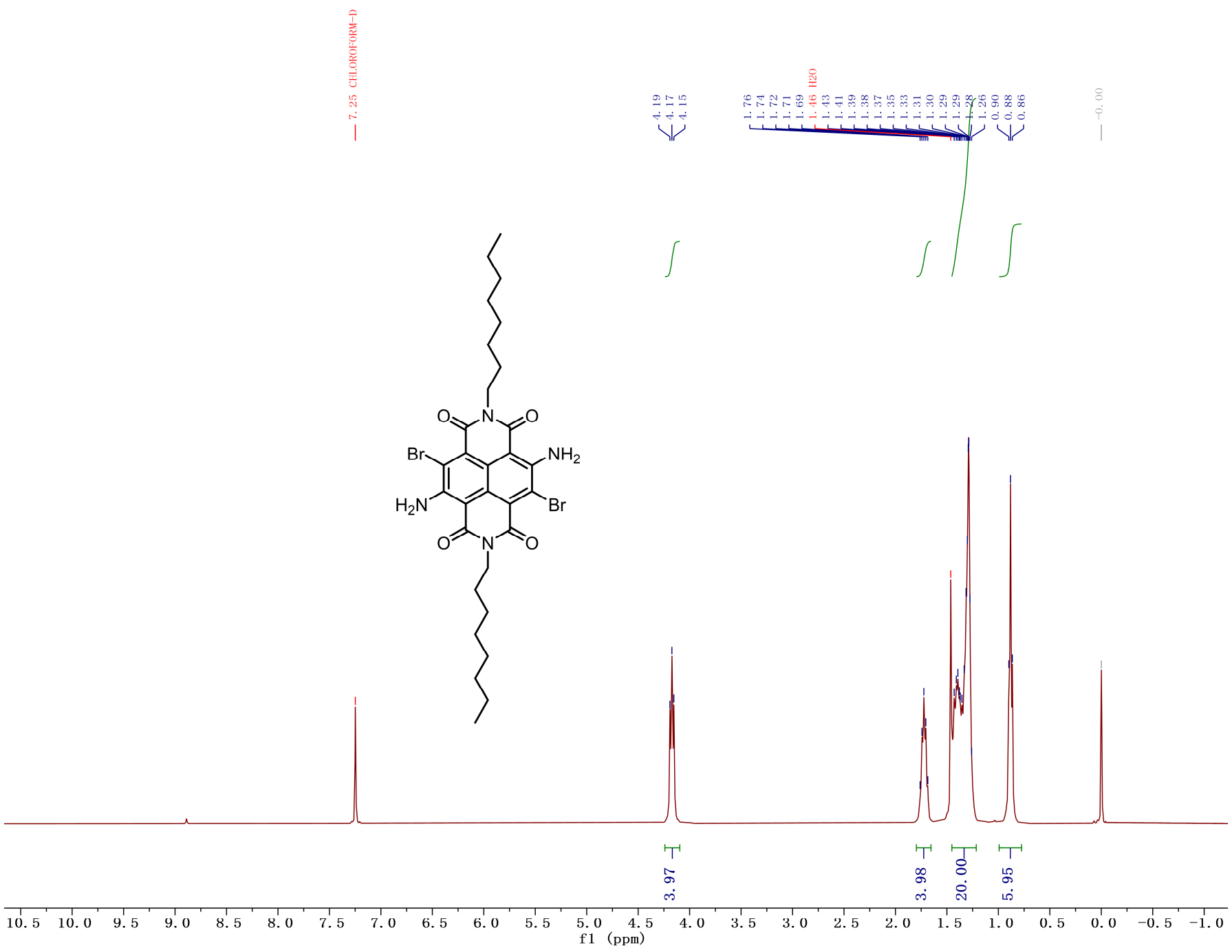

Figure S23. ^{1}H NMR spectrum (400 MHz) of **2b** in $CDCl_3$ at 328 K.

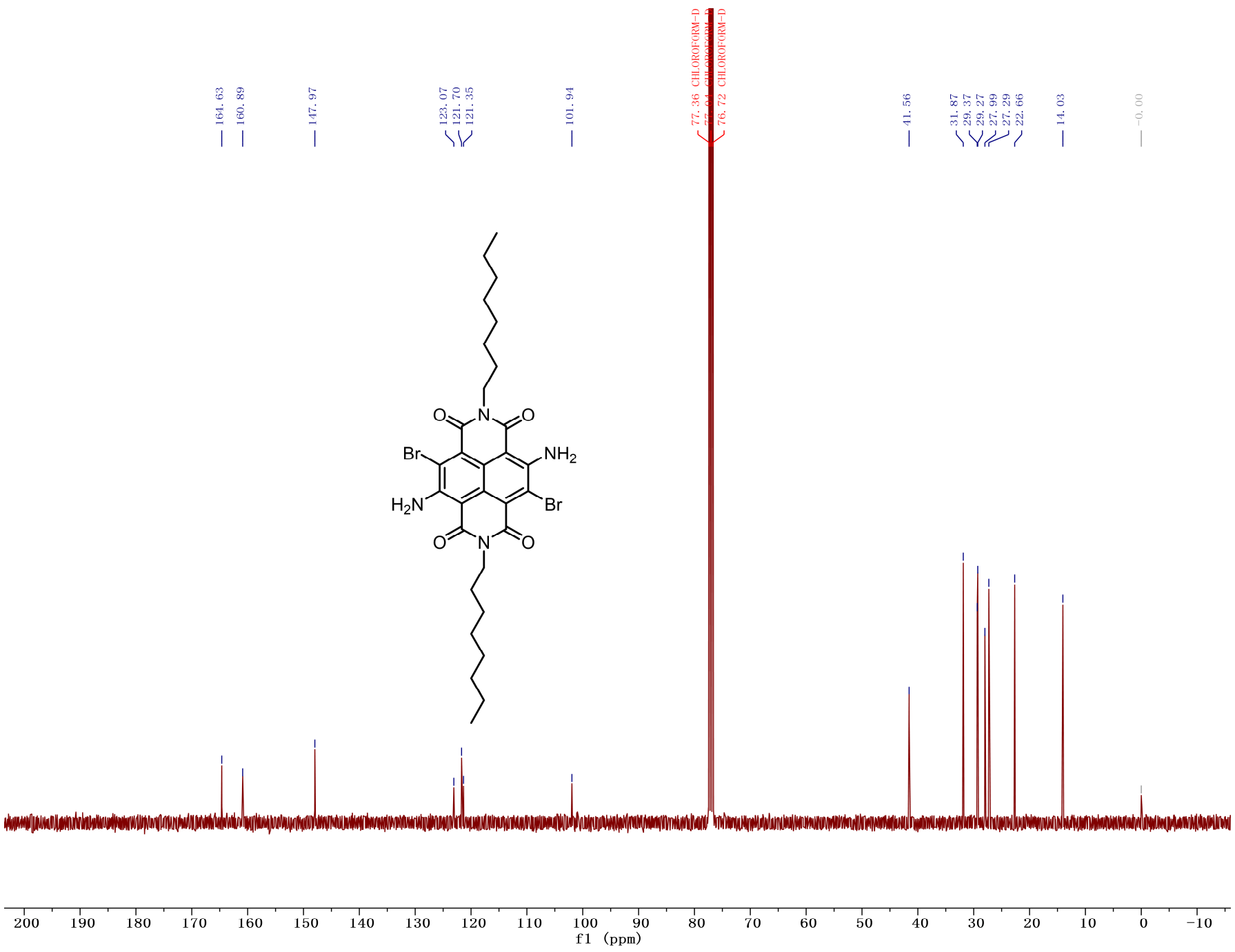

Figure S24. $^{13}C\{^{1}H\}$ NMR spectrum (100 MHz) of **2b** in $CDCl_3$ at 328 K.

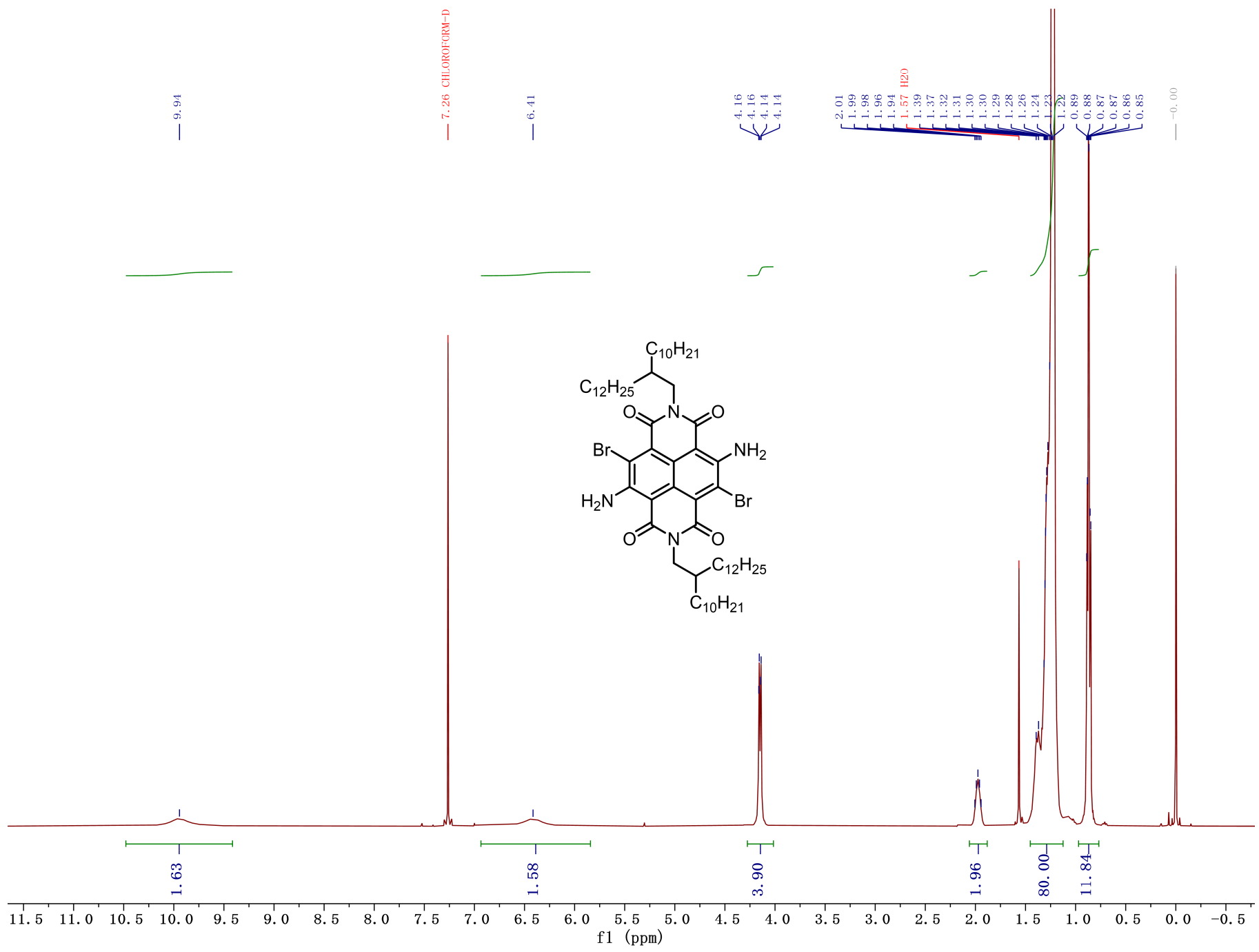

Figure S25. ^{1}H NMR spectrum (400 MHz) of **2c** in $CDCl_3$.

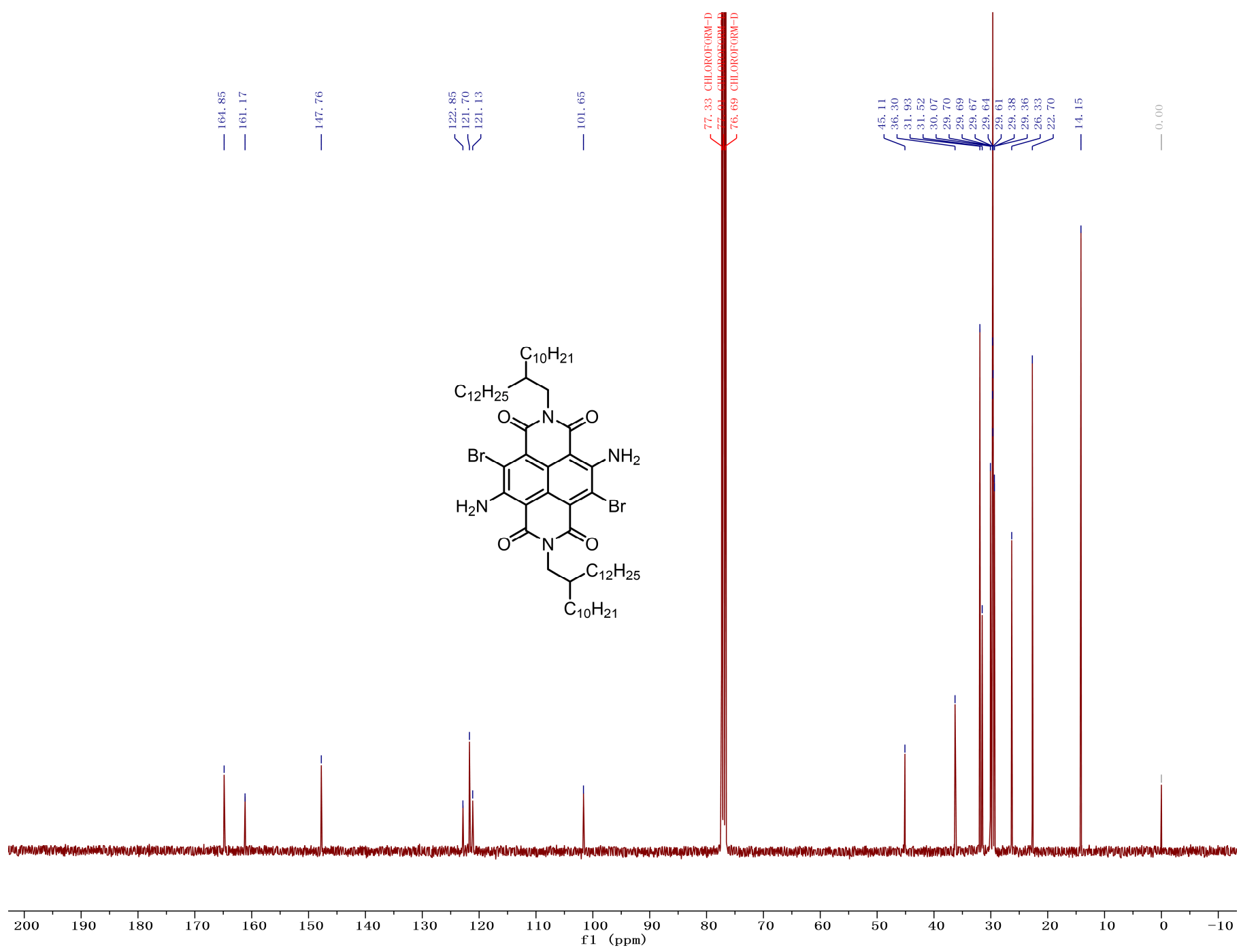

Figure S26. $^{13}C\{^{1}H\}$ NMR spectrum (100 MHz) of **2c** in $CDCl_3$.

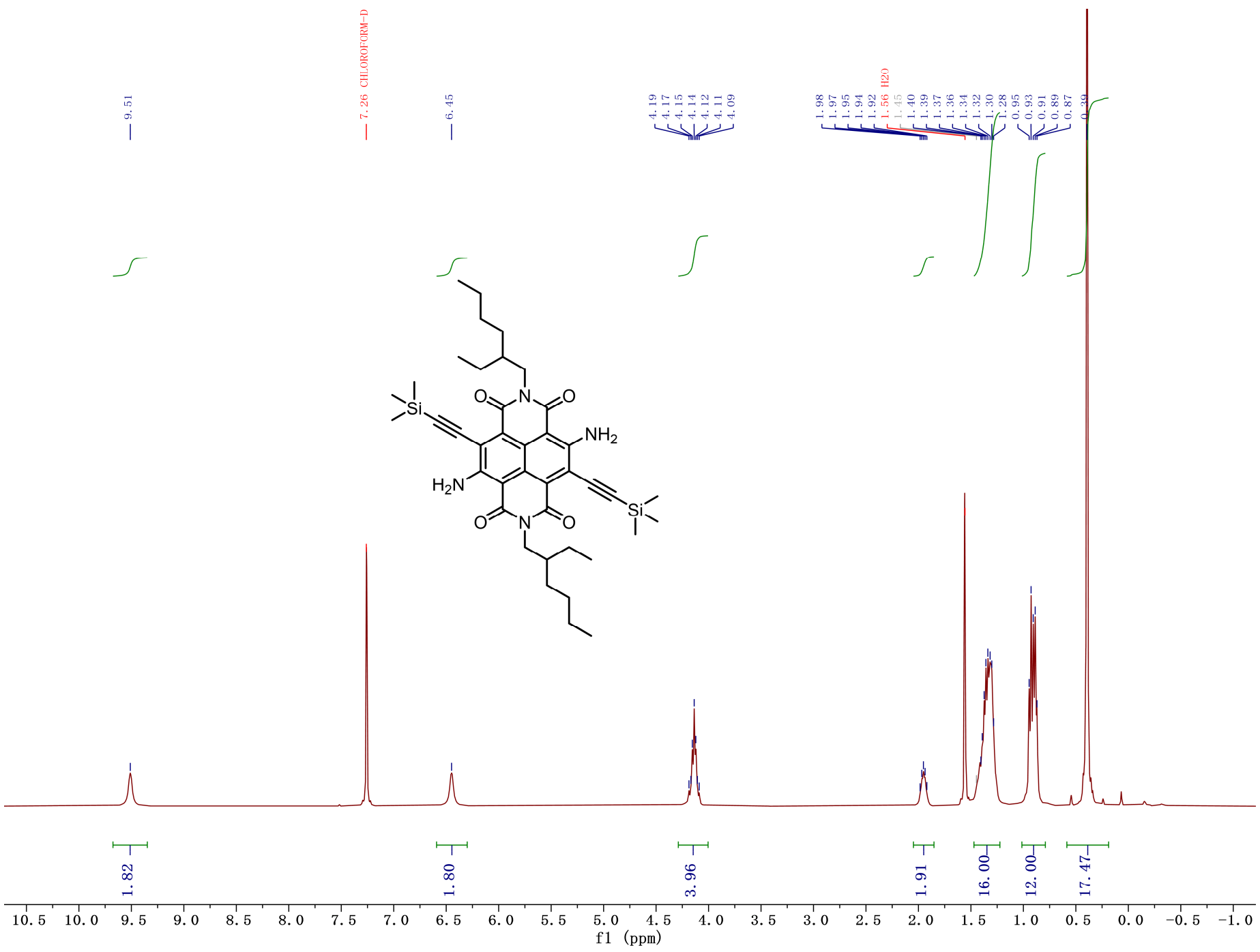

Figure S27. ^{1}H NMR spectrum (400 MHz) of **3a** in $CDCl_3$.

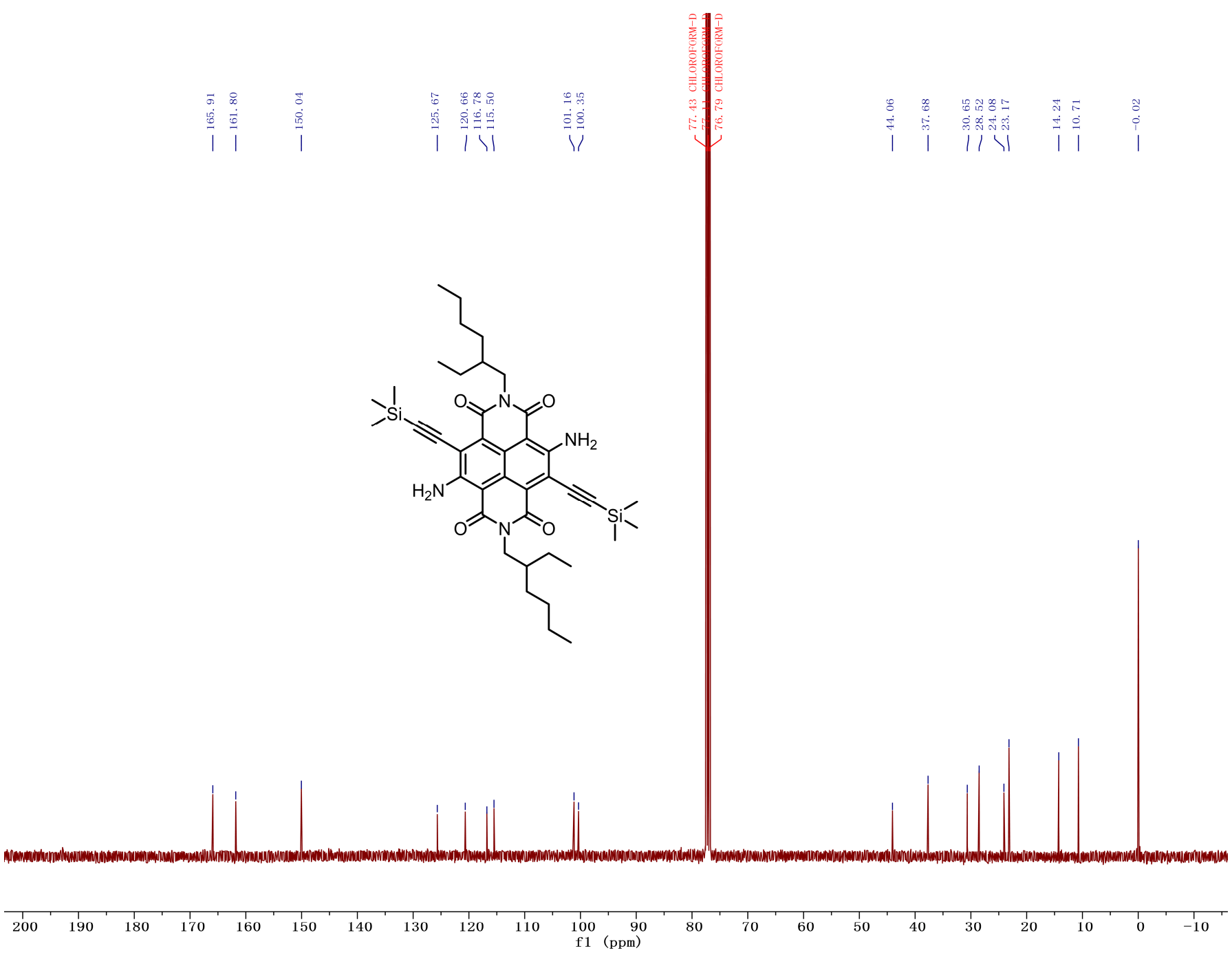

Figure S28. $^{13}C\{^{1}H\}$ NMR spectrum (100 MHz) of **3a** in $CDCl_3$.

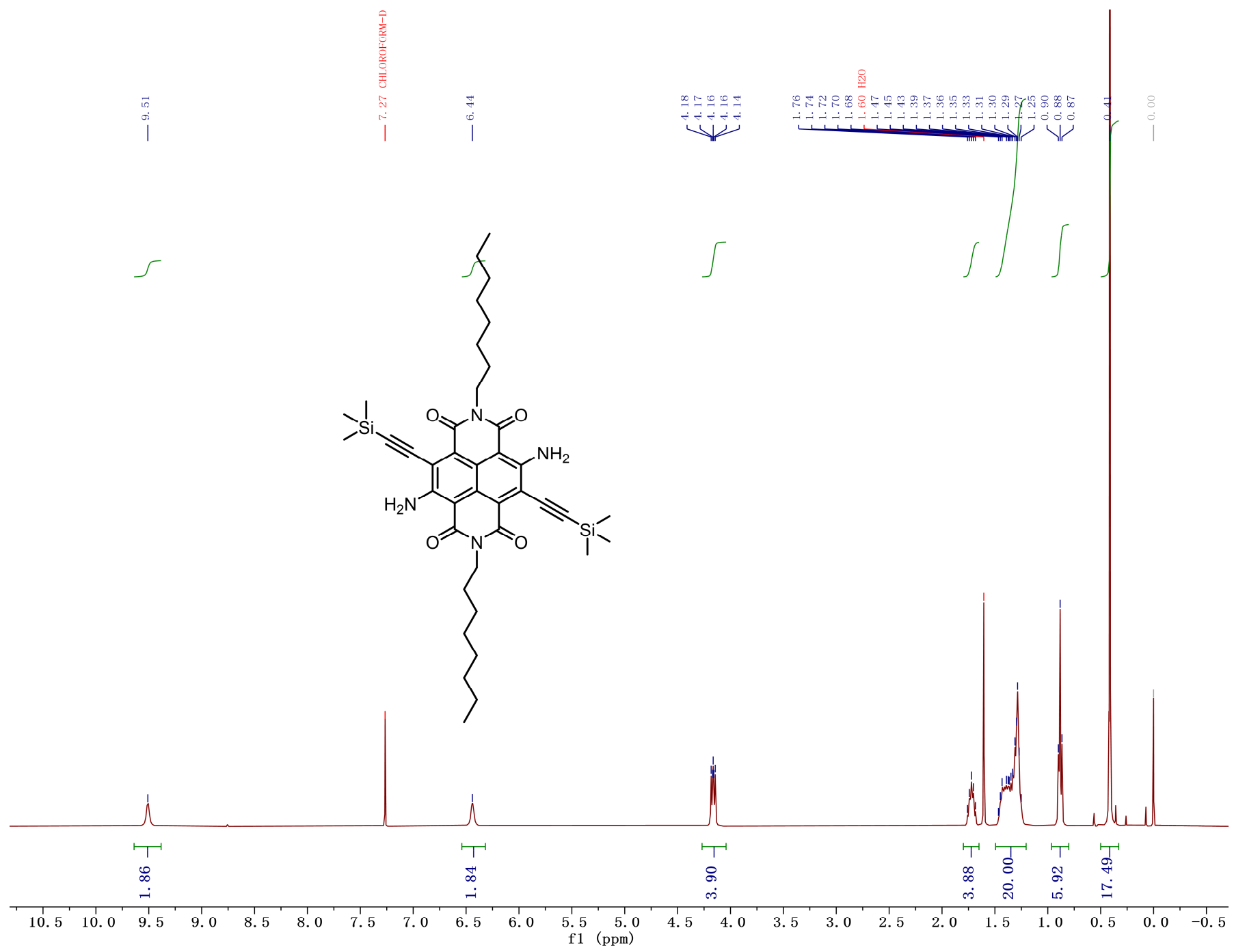

Figure S29. ^{1}H NMR spectrum (400 MHz) of **3b** in $CDCl_3$.

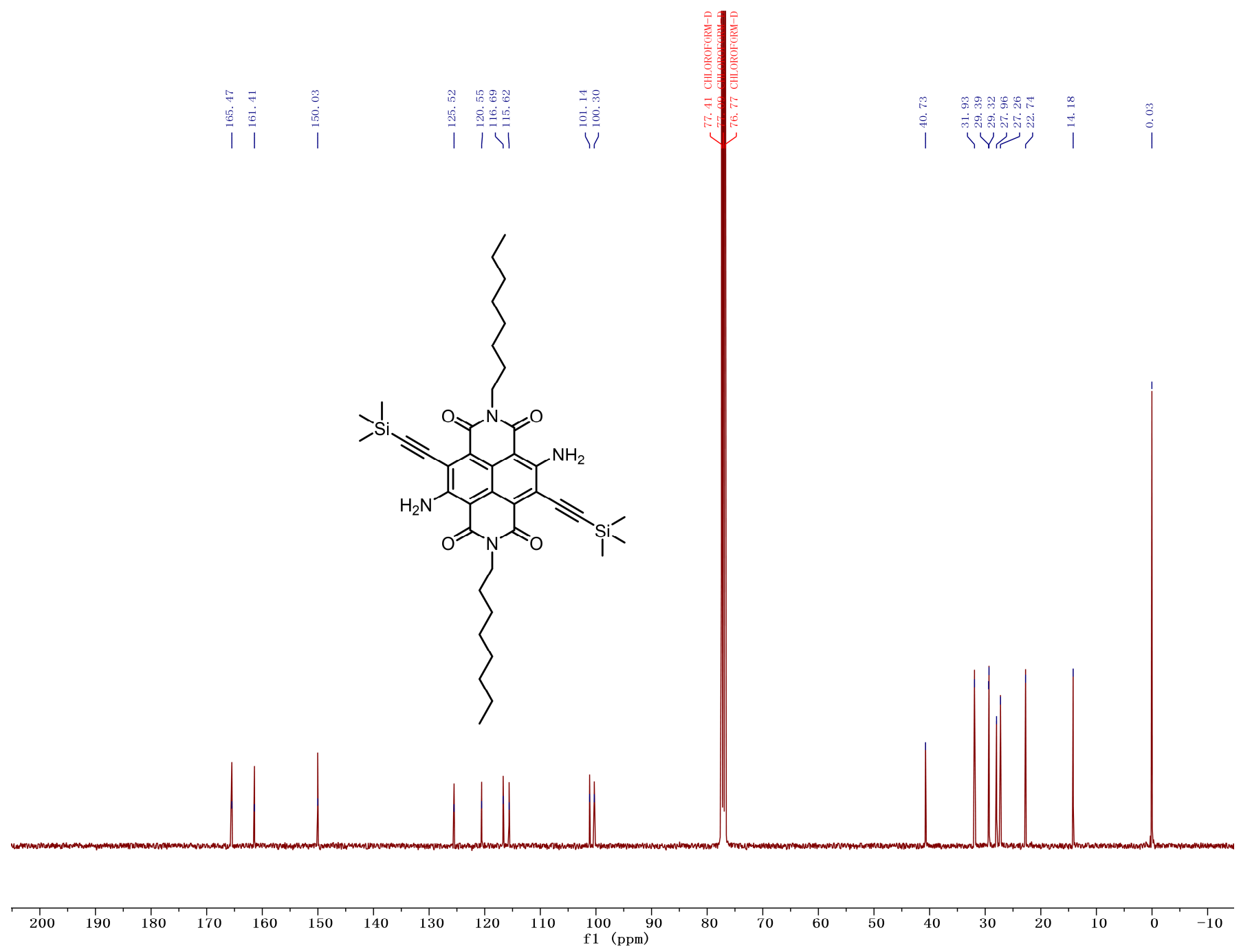

Figure S30. ^{13}C{^{1}H} NMR spectrum (100 MHz) of **3b** in $CDCl_3$.

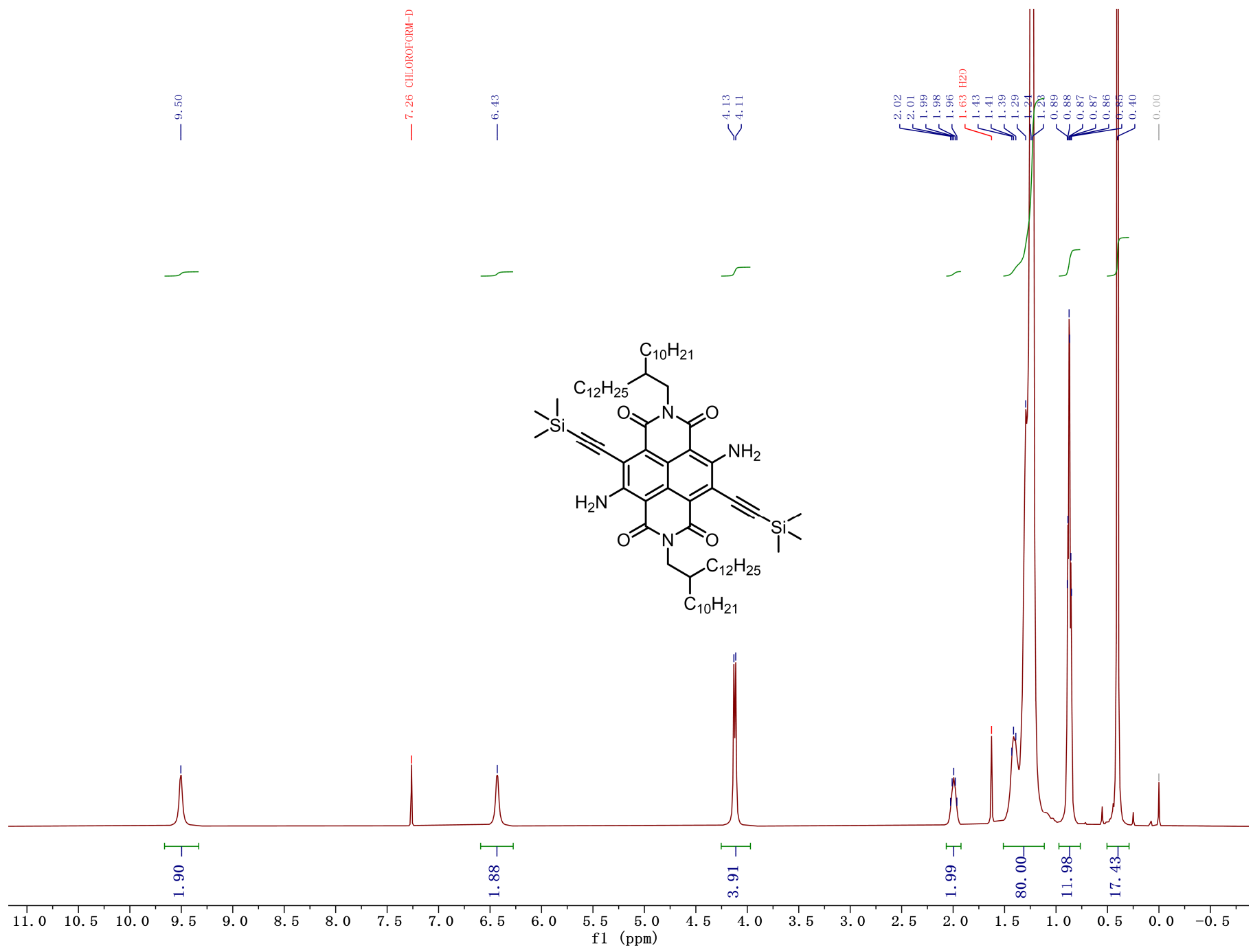

Figure S31. ^{1}H NMR spectrum (400 MHz) of **3c** in $CDCl_3$.

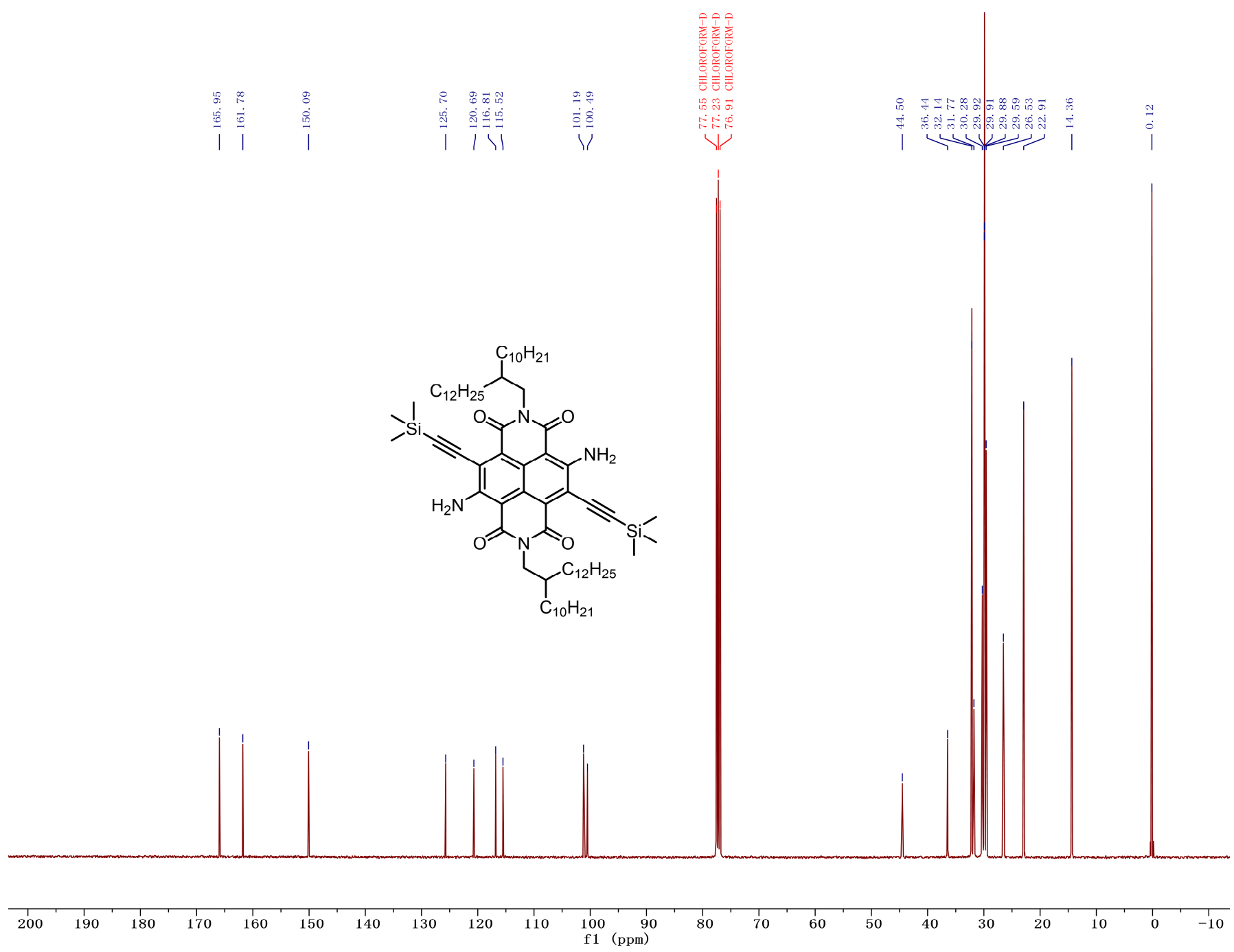

Figure S32. $^{13}C\{^1H\}$ NMR spectrum (100 MHz) of **3c** in $CDCl_3$.

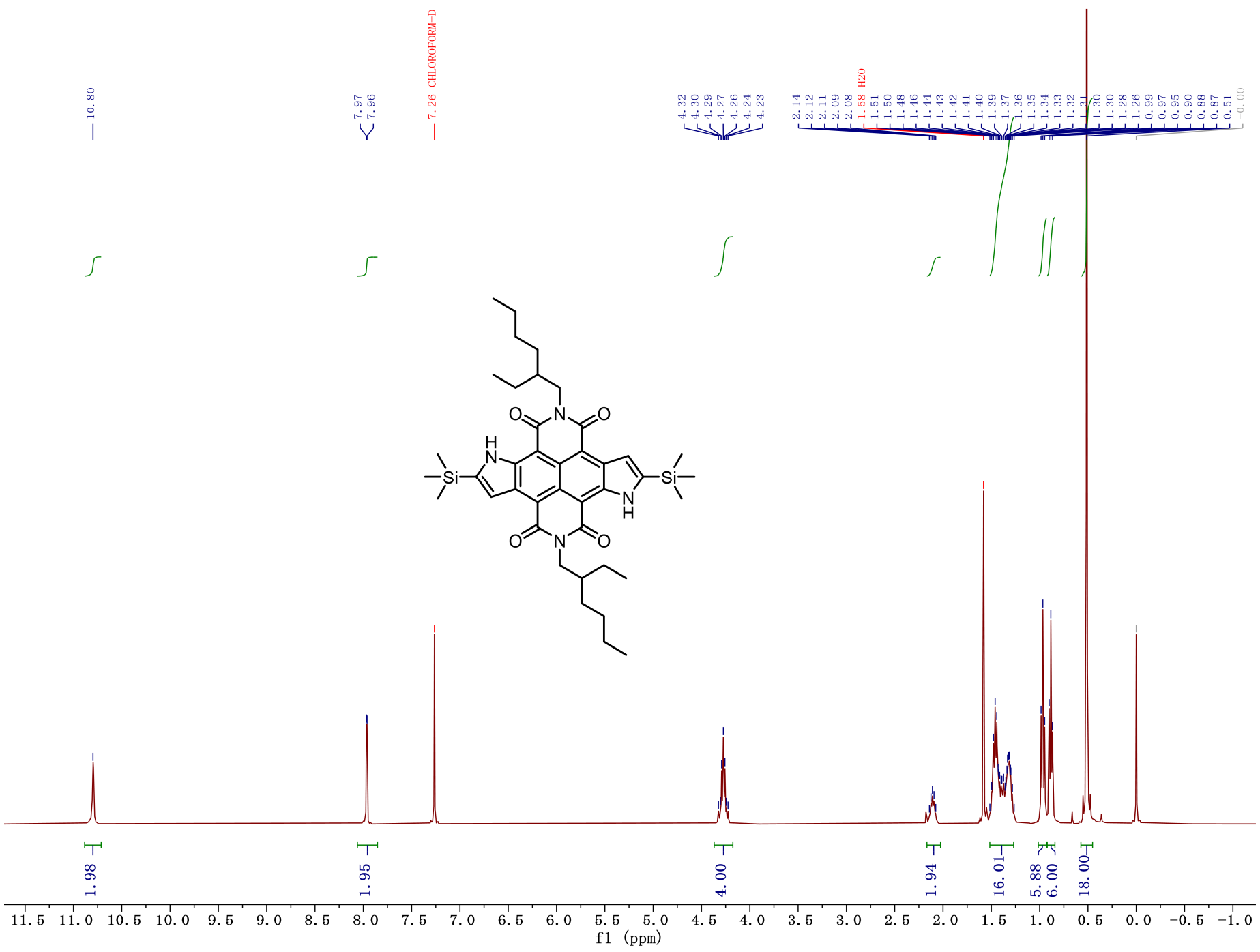

Figure S33. ^{1}H NMR spectrum (400 MHz) of **4a** in $CDCl_3$.

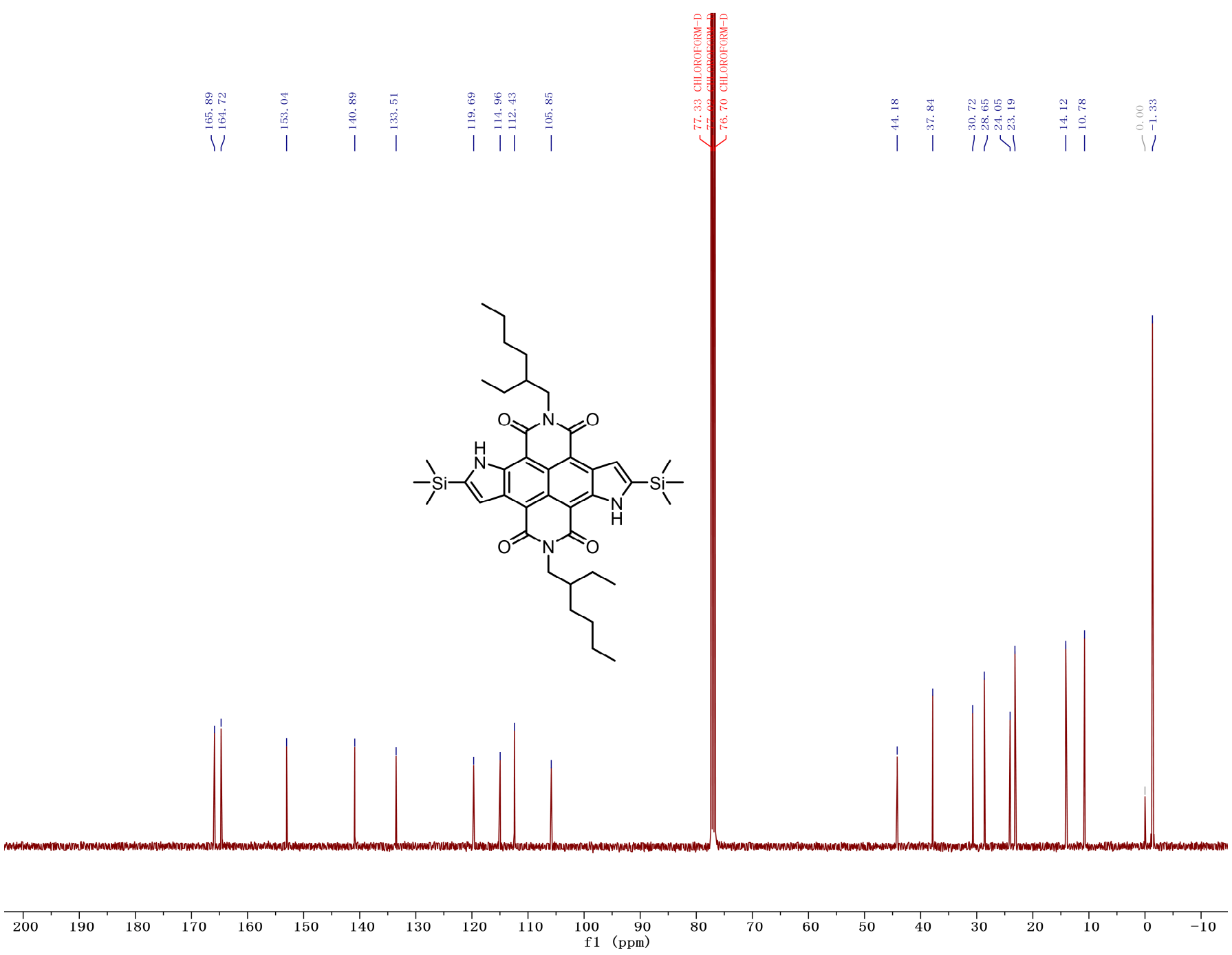

Figure S34. $^{13}C\{^{1}H\}$ NMR spectrum (100 MHz) of **4a** in $CDCl_3$.

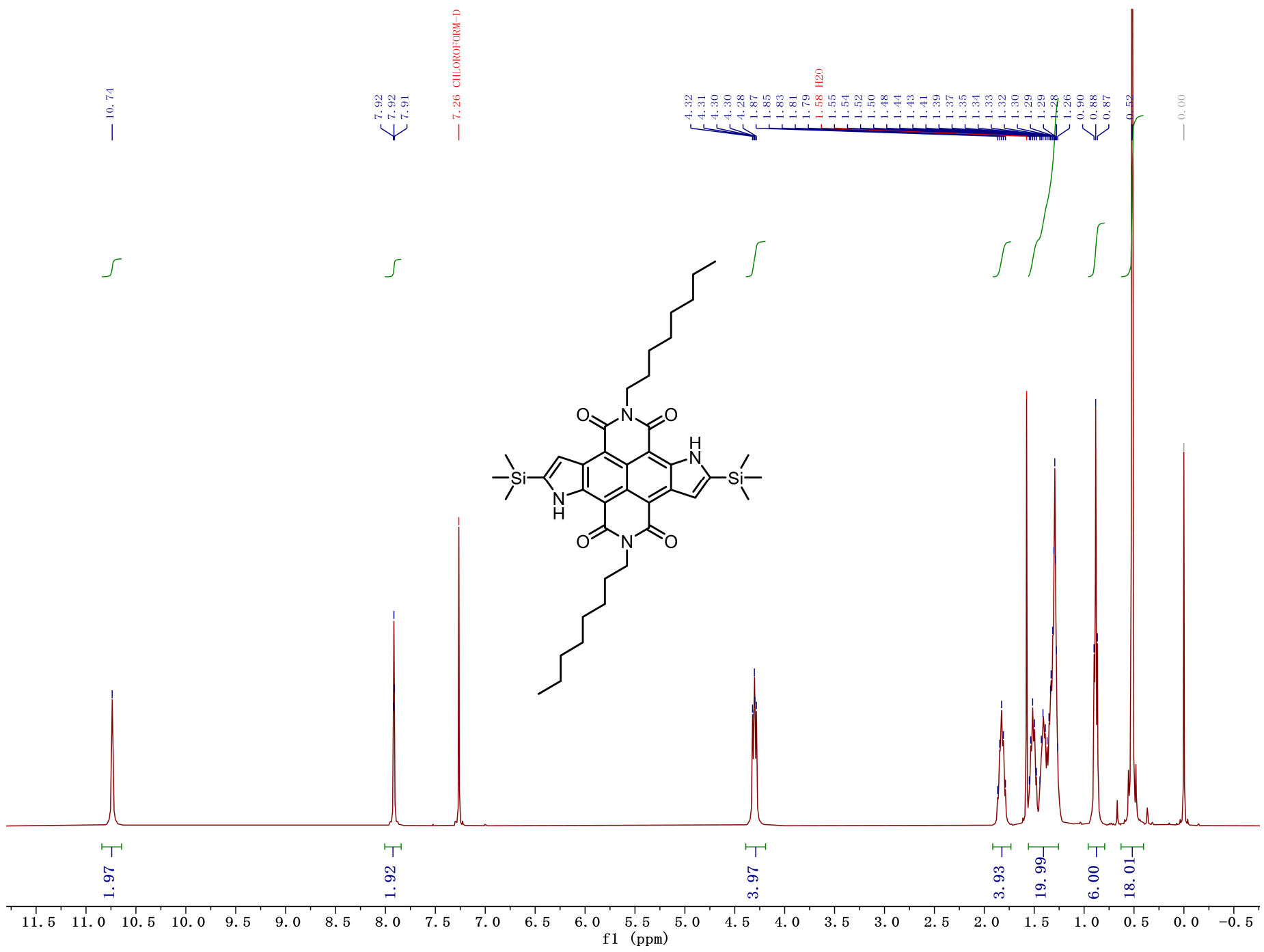

Figure S35. ^{1}H NMR spectrum (400 MHz) of **4b** in $CDCl_3$.

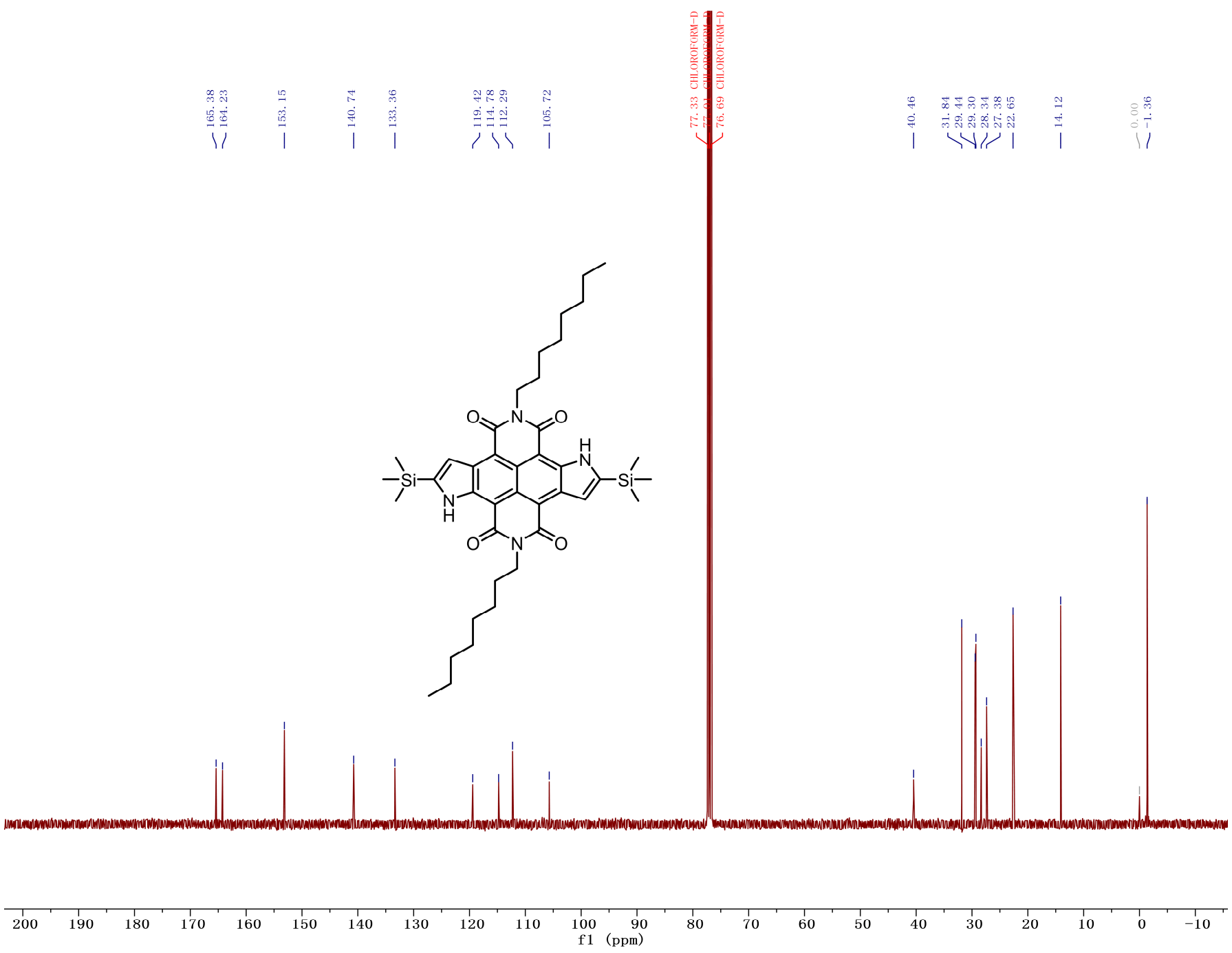

Figure S36. $^{13}C\{^{1}H\}$ NMR spectrum (100 MHz) of **4b** in $CDCl_3$.

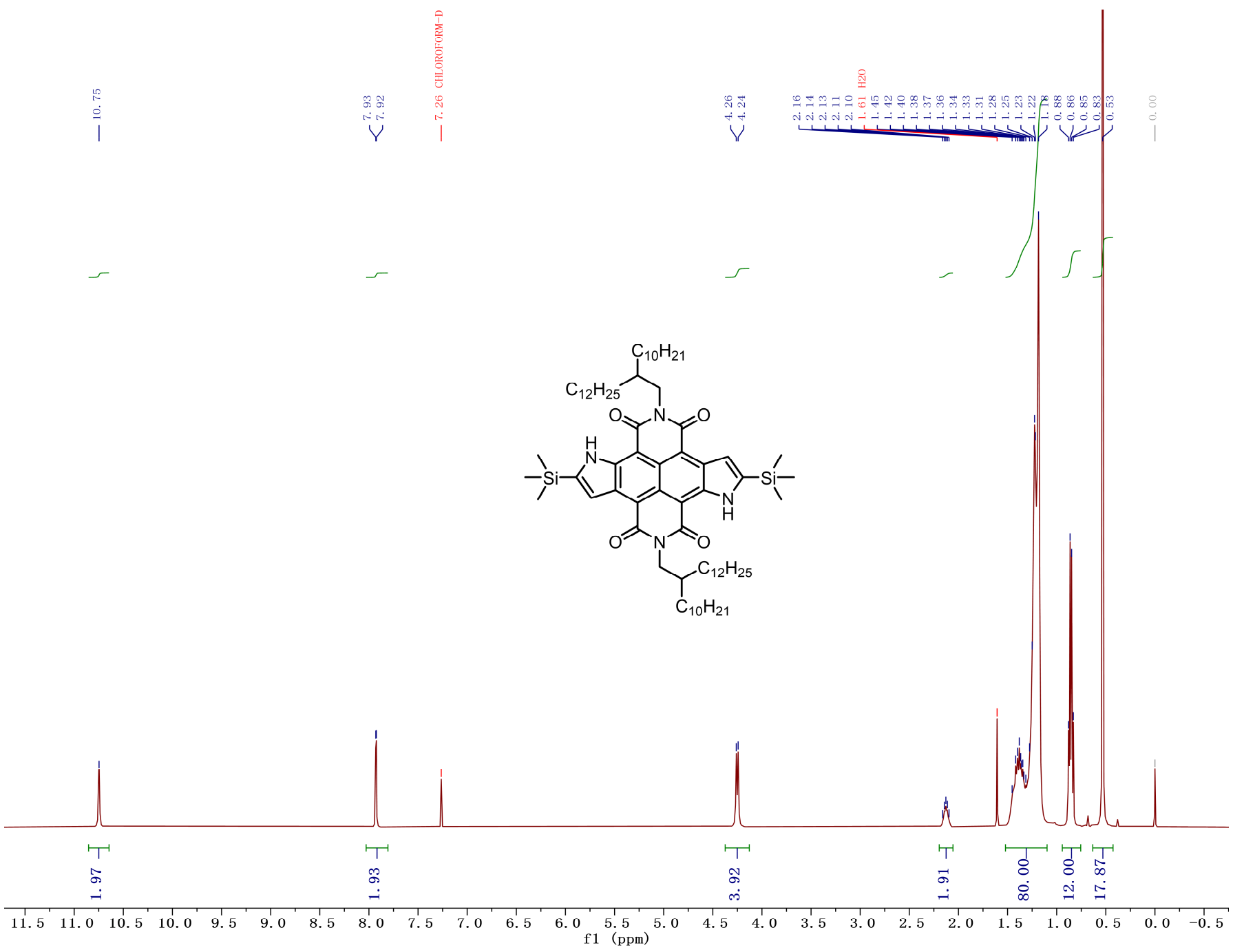

Figure S37. ^{1}H NMR spectrum (400 MHz) of **4c** in $CDCl_3$.

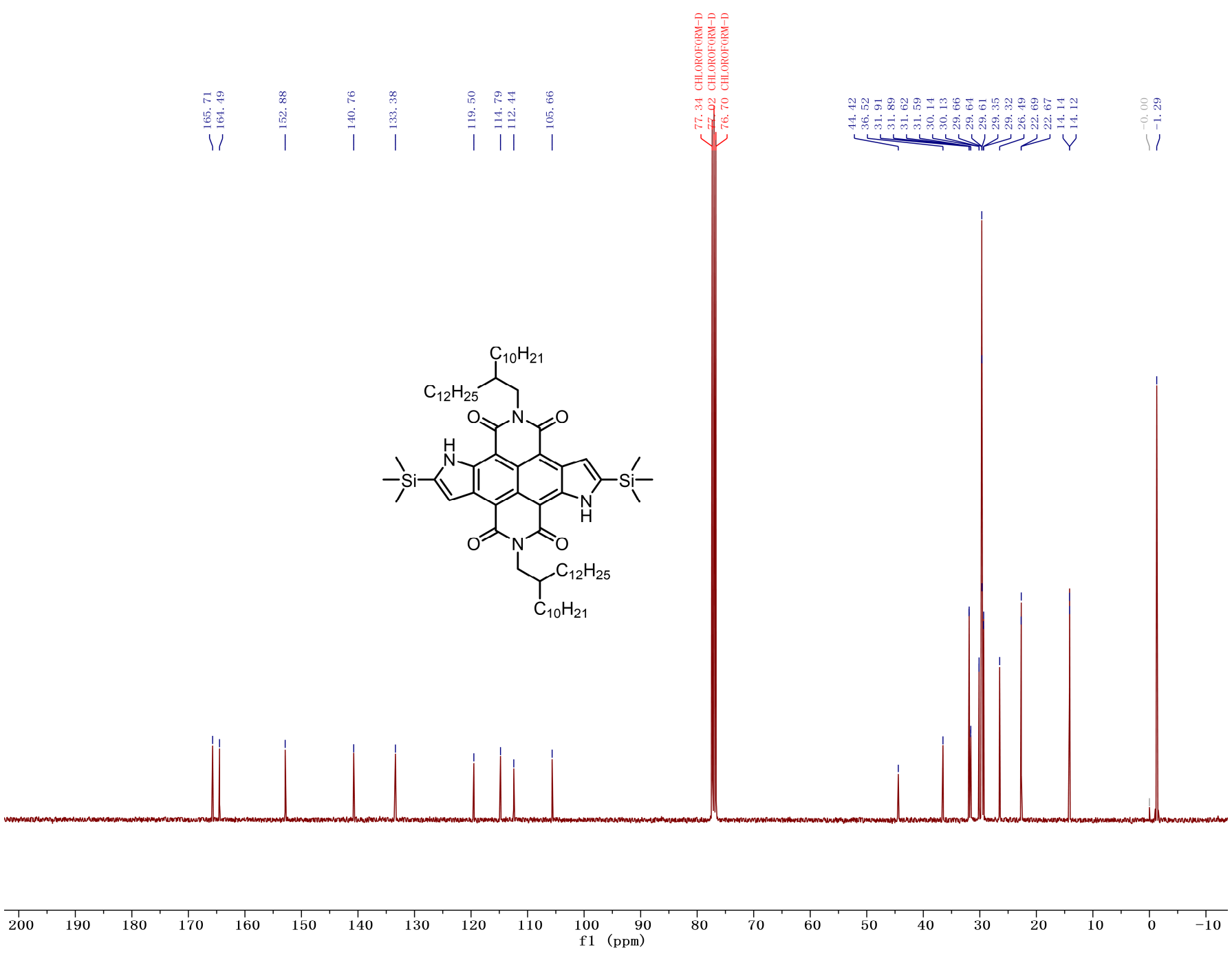

Figure S38. $^{13}C\{^1H\}$ NMR spectrum (100 MHz) of **4c** in $CDCl_3$.

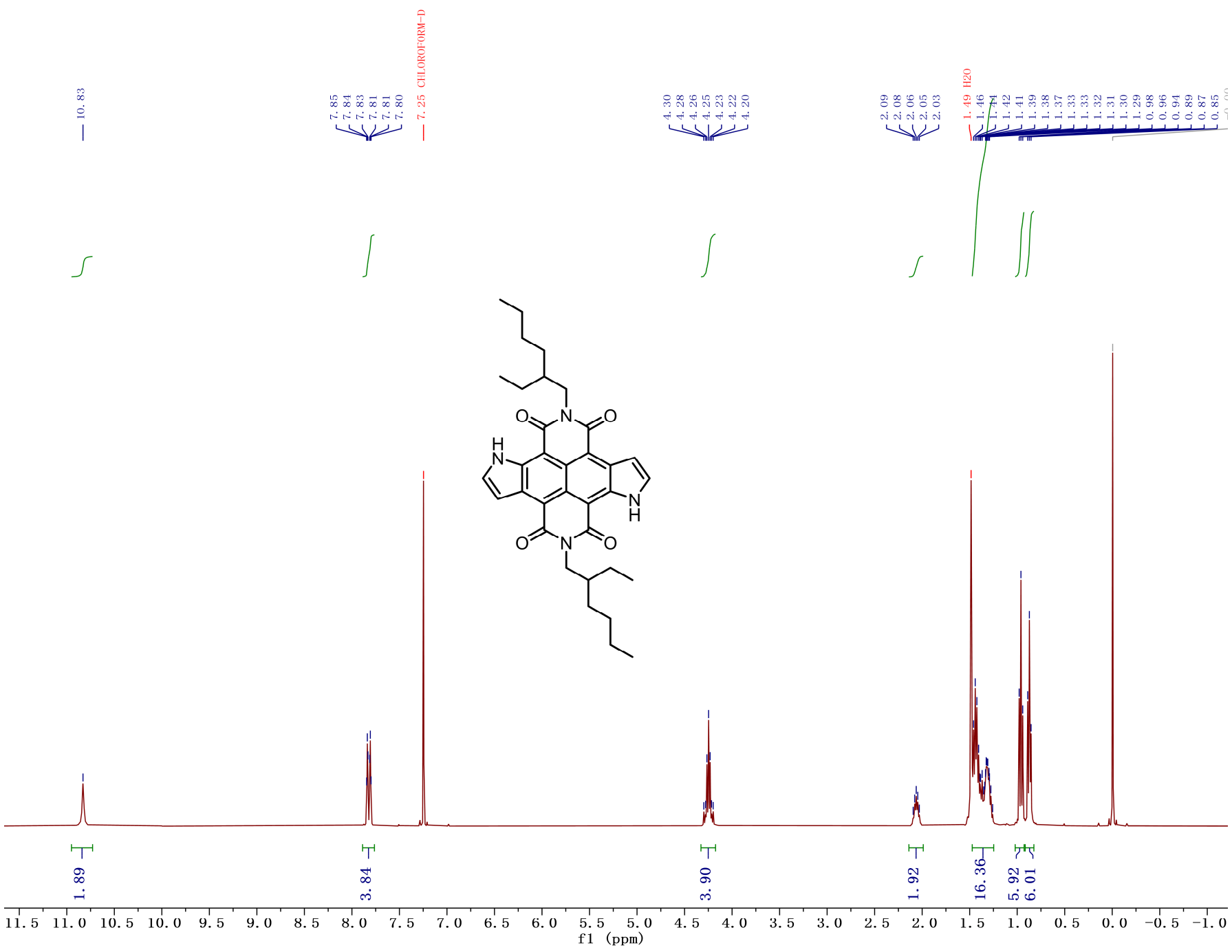

Figure S39. ^{1}H NMR spectrum (400 MHz) of **5a** in 1,1,2,2-$C_2D_2Cl_4$ at 363 K.

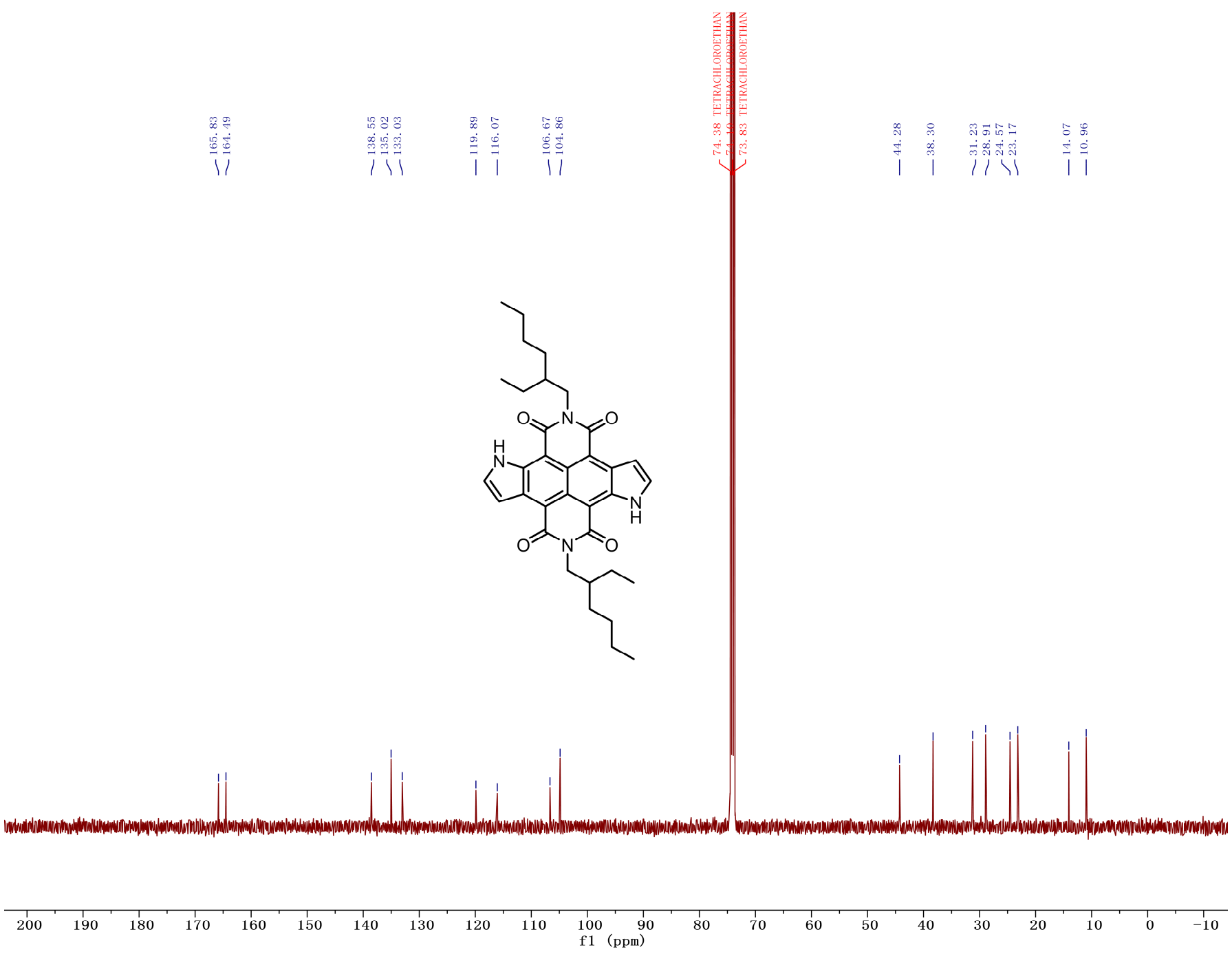

Figure S40. $^{13}C\{^{1}H\}$ NMR spectrum (100 MHz) of **5a** in 1,1,2,2-$C_2D_2Cl_4$ at 363 K.

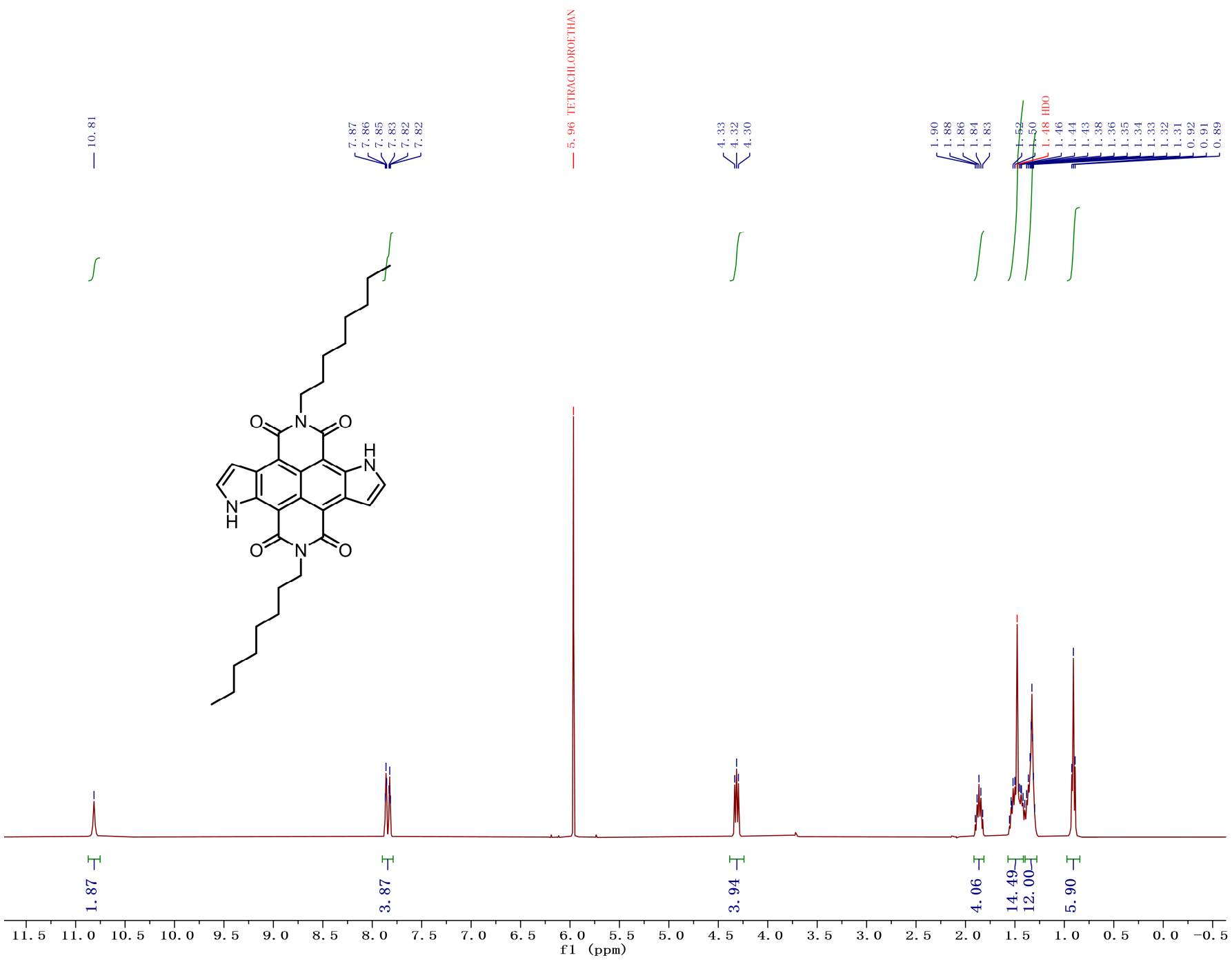

Figure S41. ^{1}H NMR spectrum (400 MHz) of **5b** in 1,1,2,2-$C_2D_2Cl_4$ at 363 K.

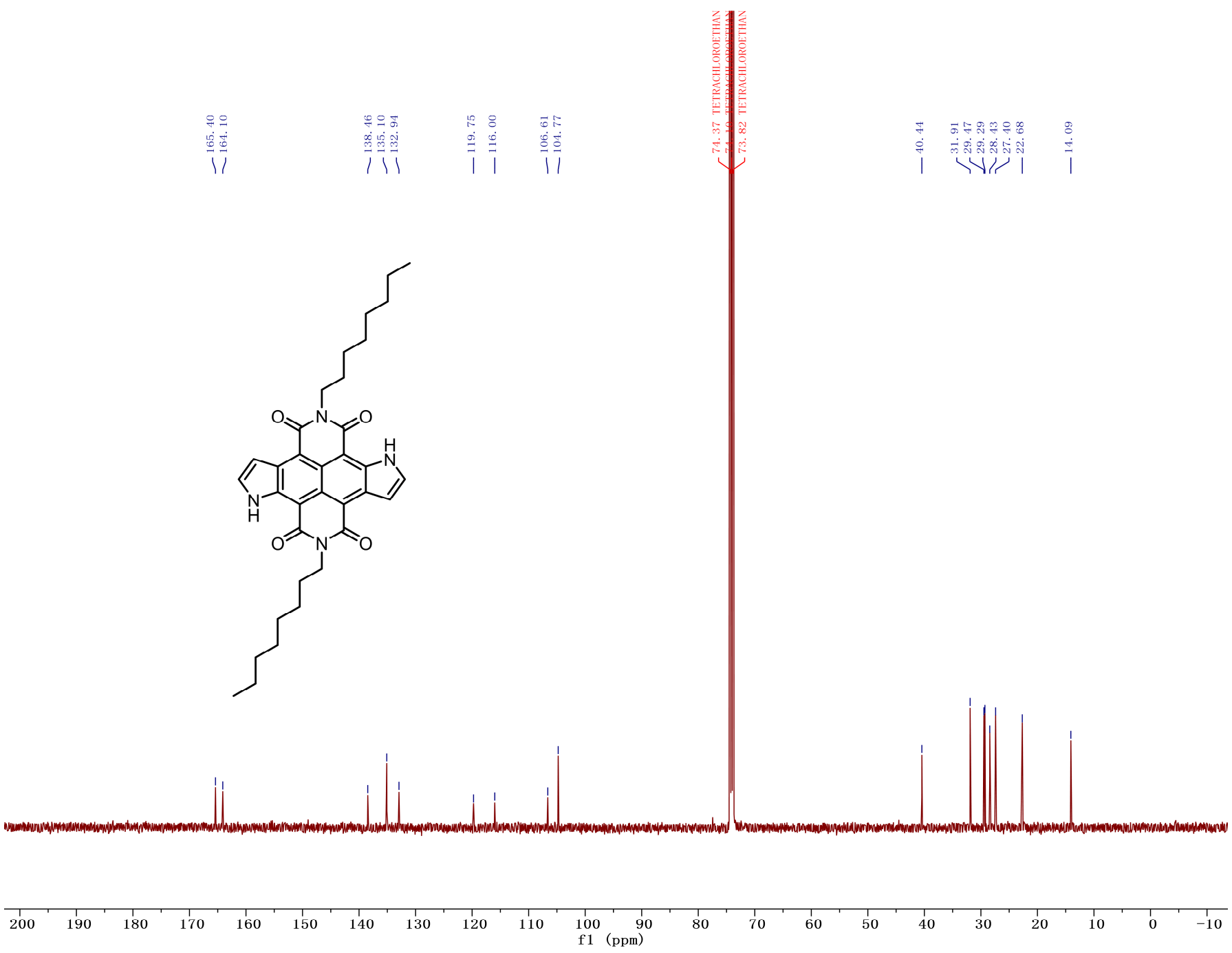

Figure S42. $^{13}C\{^{1}H\}$ NMR spectrum (100 MHz) of **5b** in 1,1,2,2-$C_2D_2Cl_4$ at 363 K.

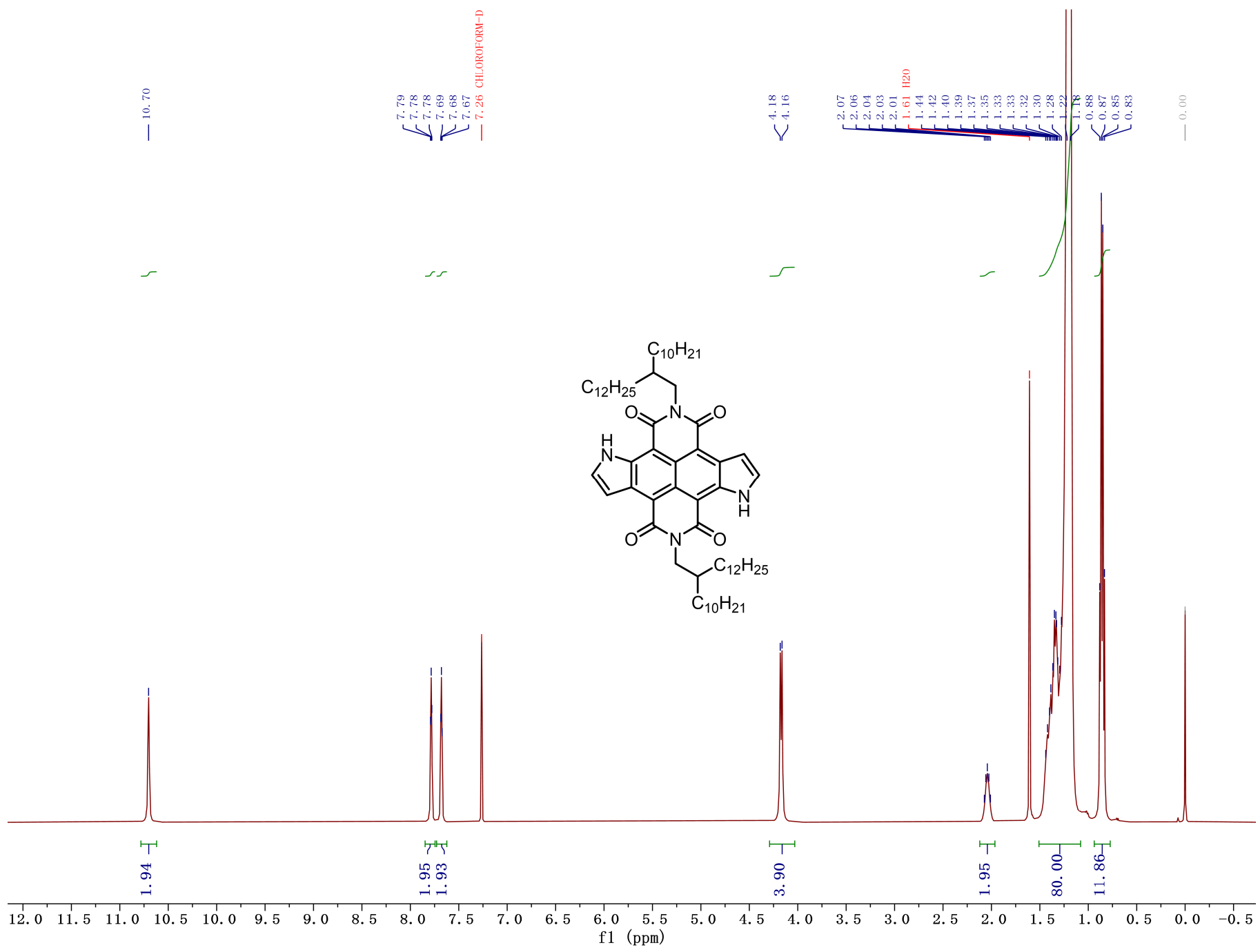

Figure S43. ^{1}H NMR spectrum (400 MHz) of **5c** in $CDCl_3$.

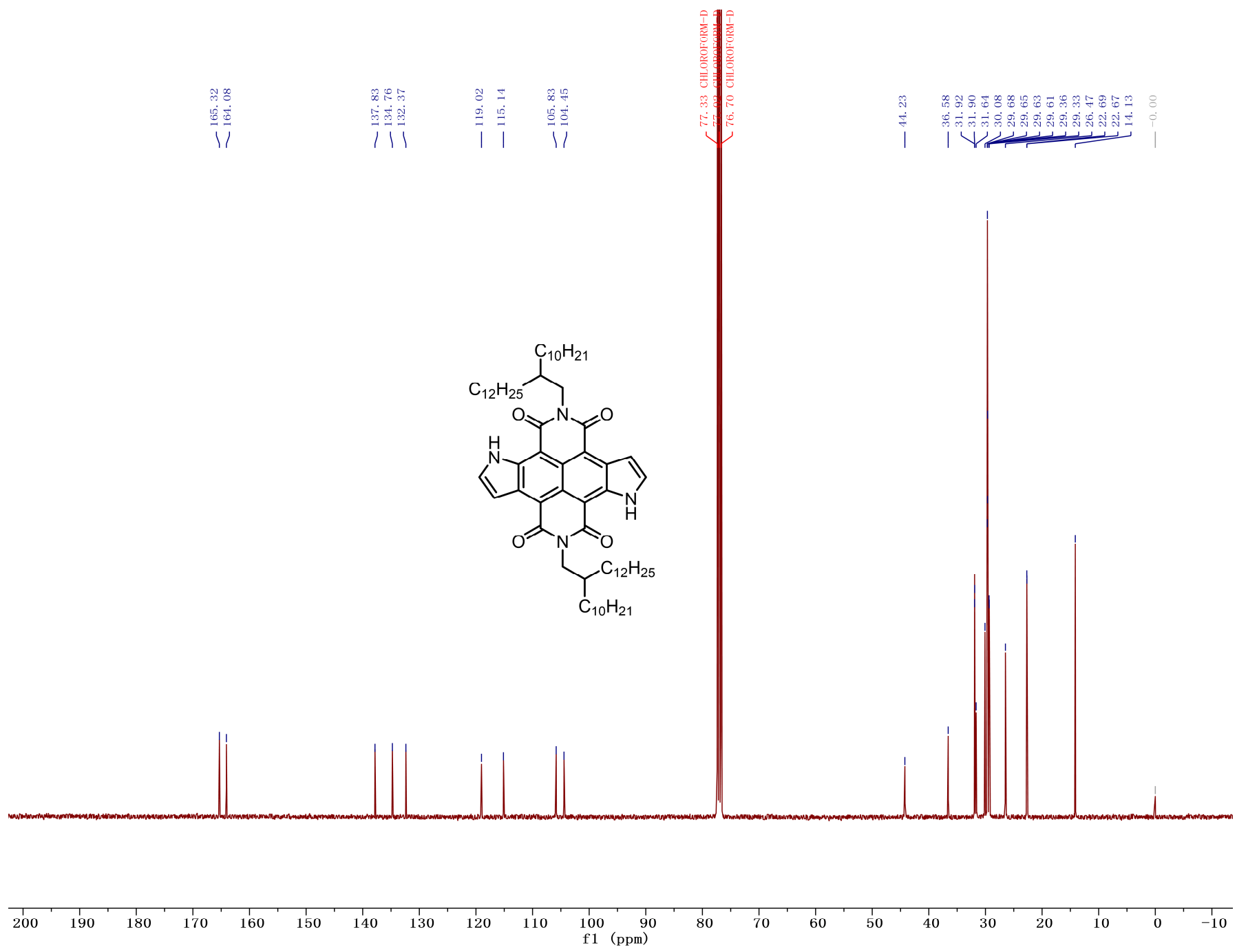

Figure S44. $^{13}C\{^{1}H\}$ NMR spectrum (100 MHz) of **5c** in $CDCl_3$.

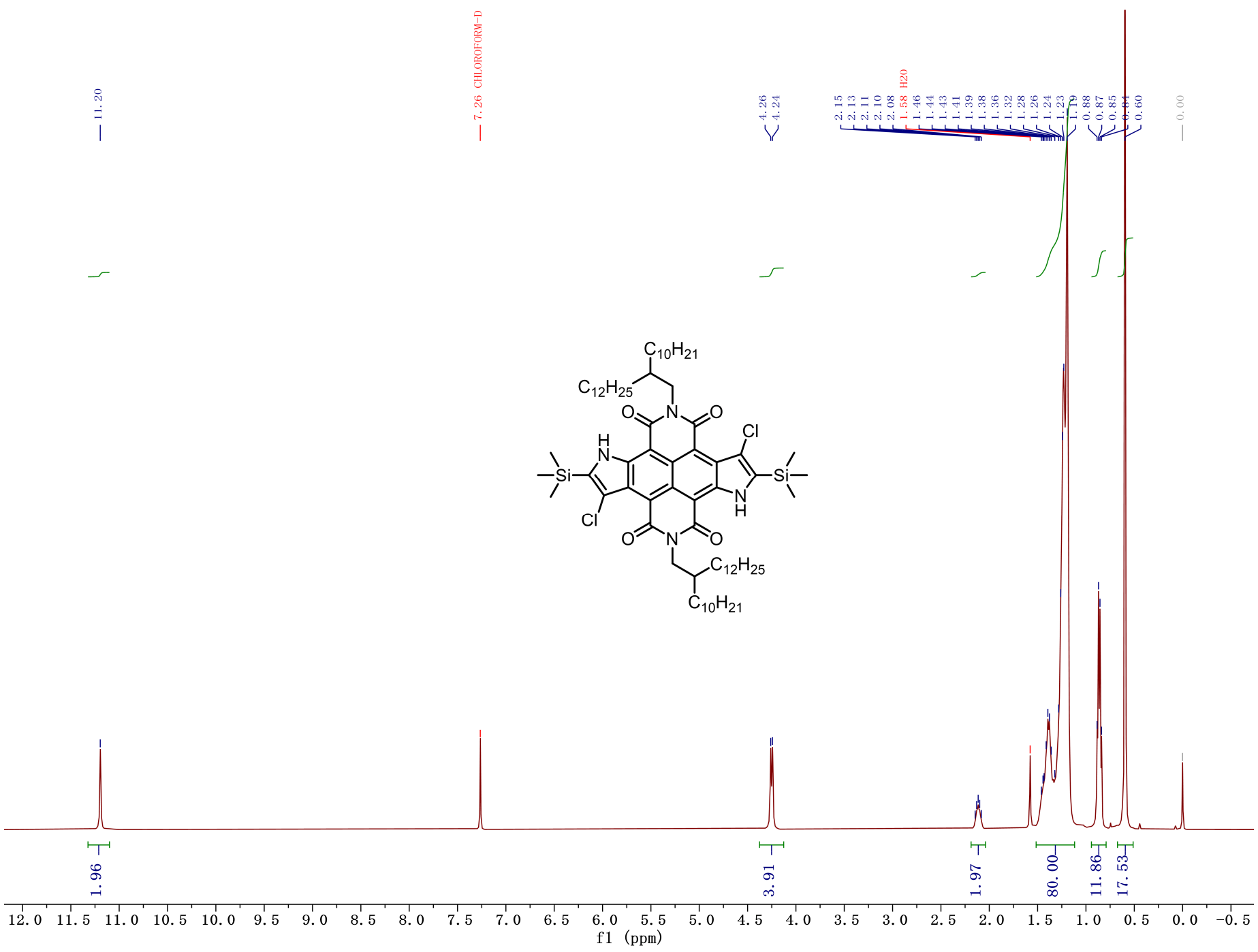

Figure S45. ^{1}H NMR spectrum (400 MHz) of **6** in $CDCl_3$.

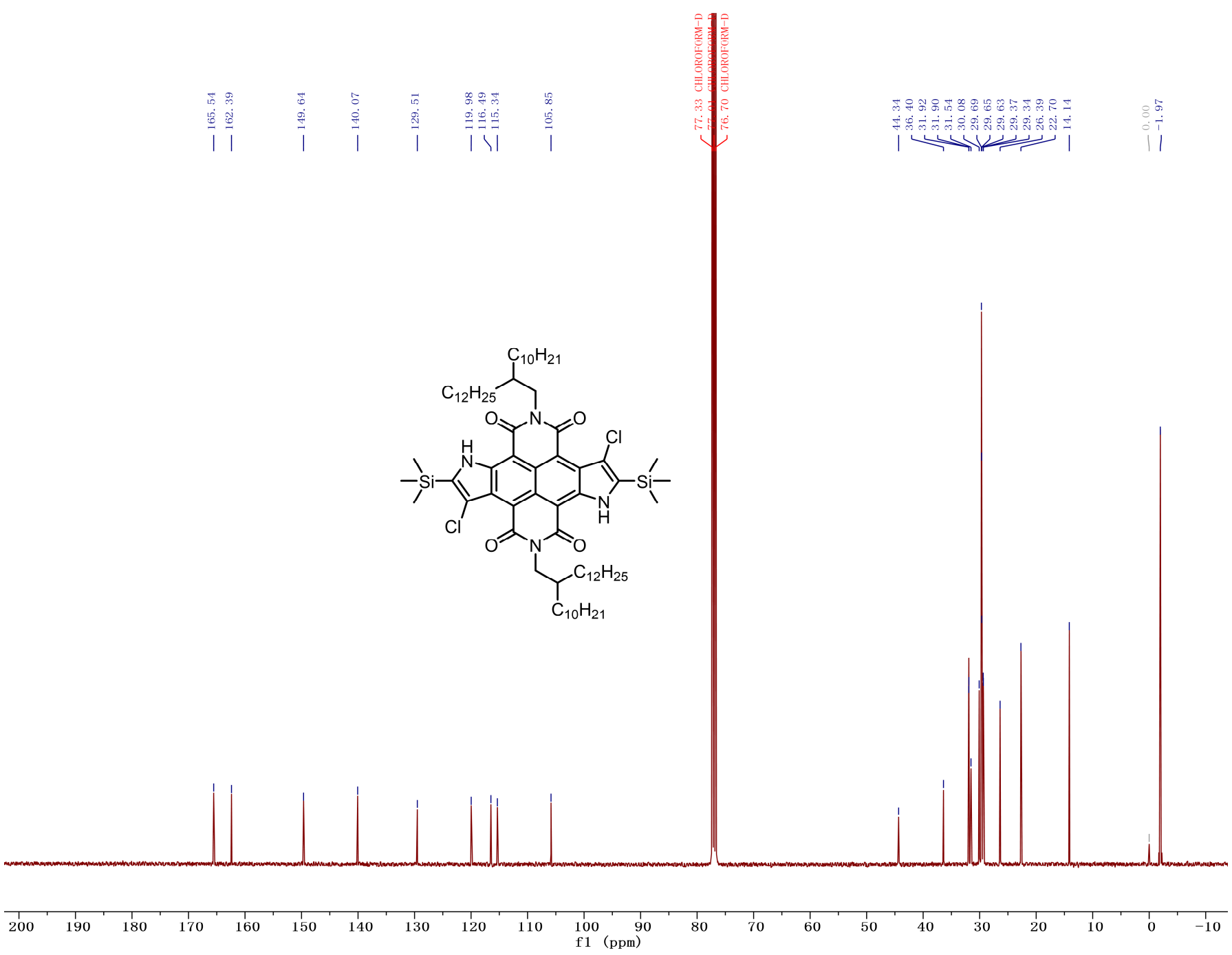

Figure S46. $^{13}C\{^1H\}$ NMR spectrum (100 MHz) of **6** in in $CDCl_3$.

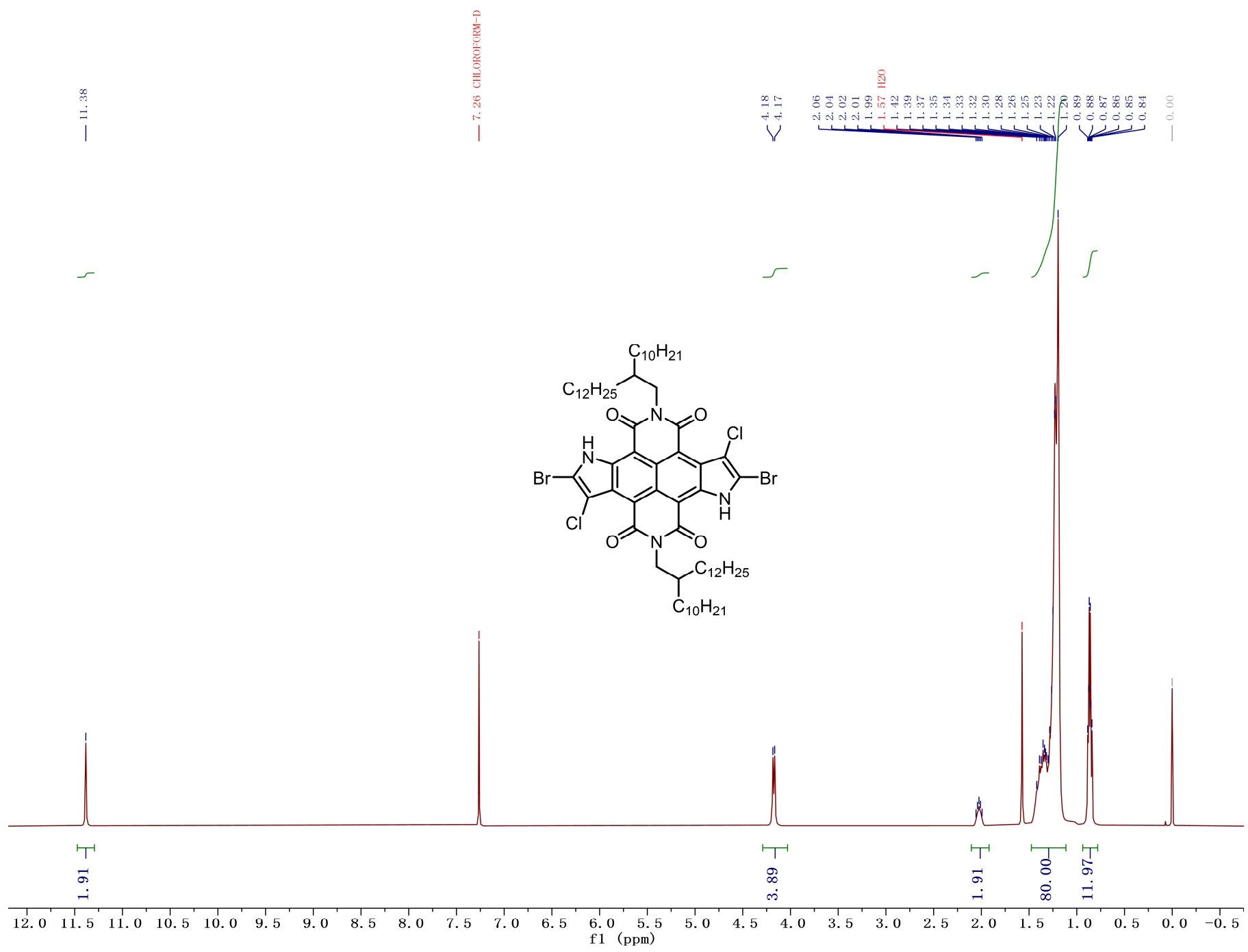

Figure S47. ^{1}H NMR spectrum (400 MHz) of **7** in $CDCl_3$.

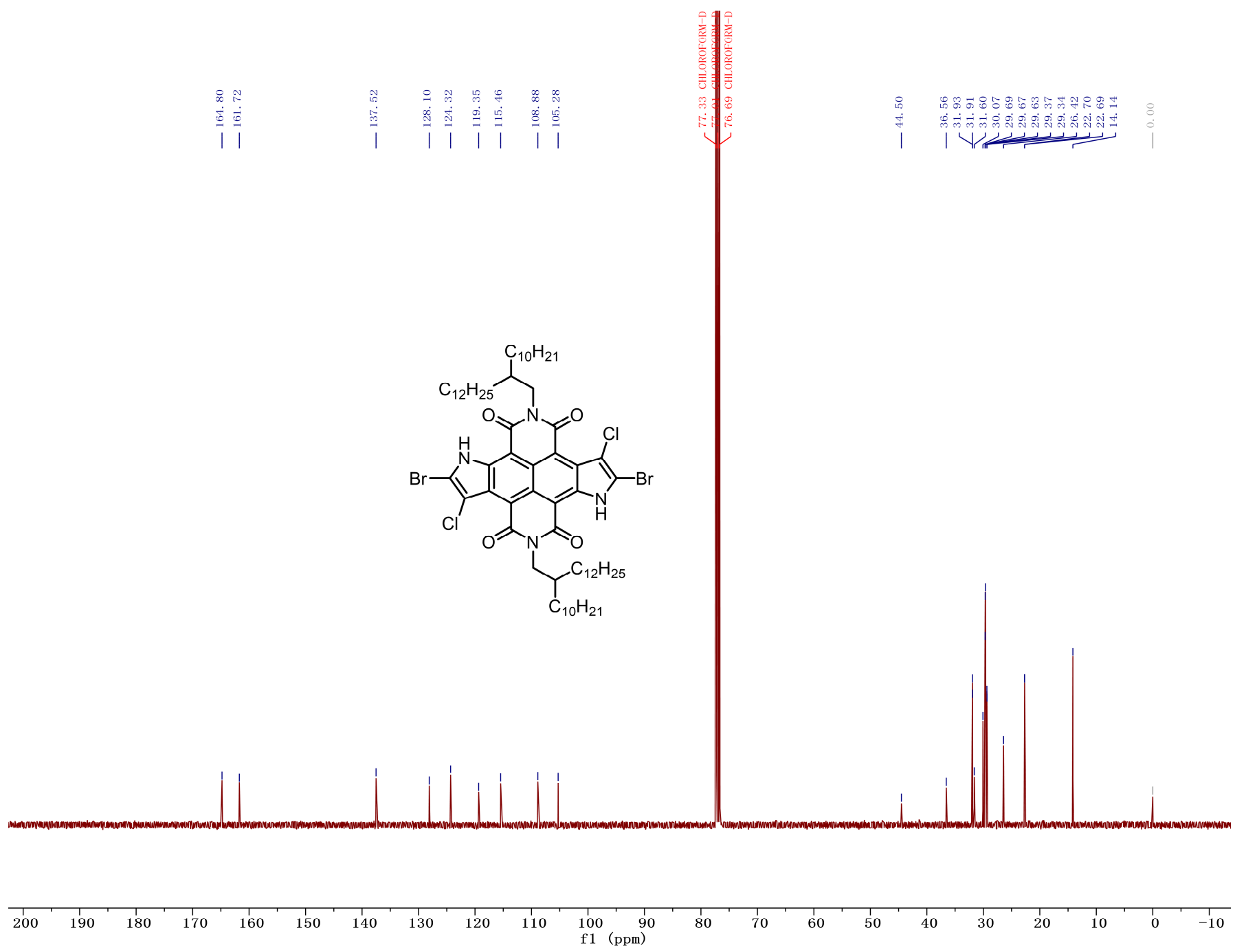

Figure S48. $^{13}C\{^{1}H\}$ NMR spectrum (100 MHz) of **7** in in $CDCl_3$.

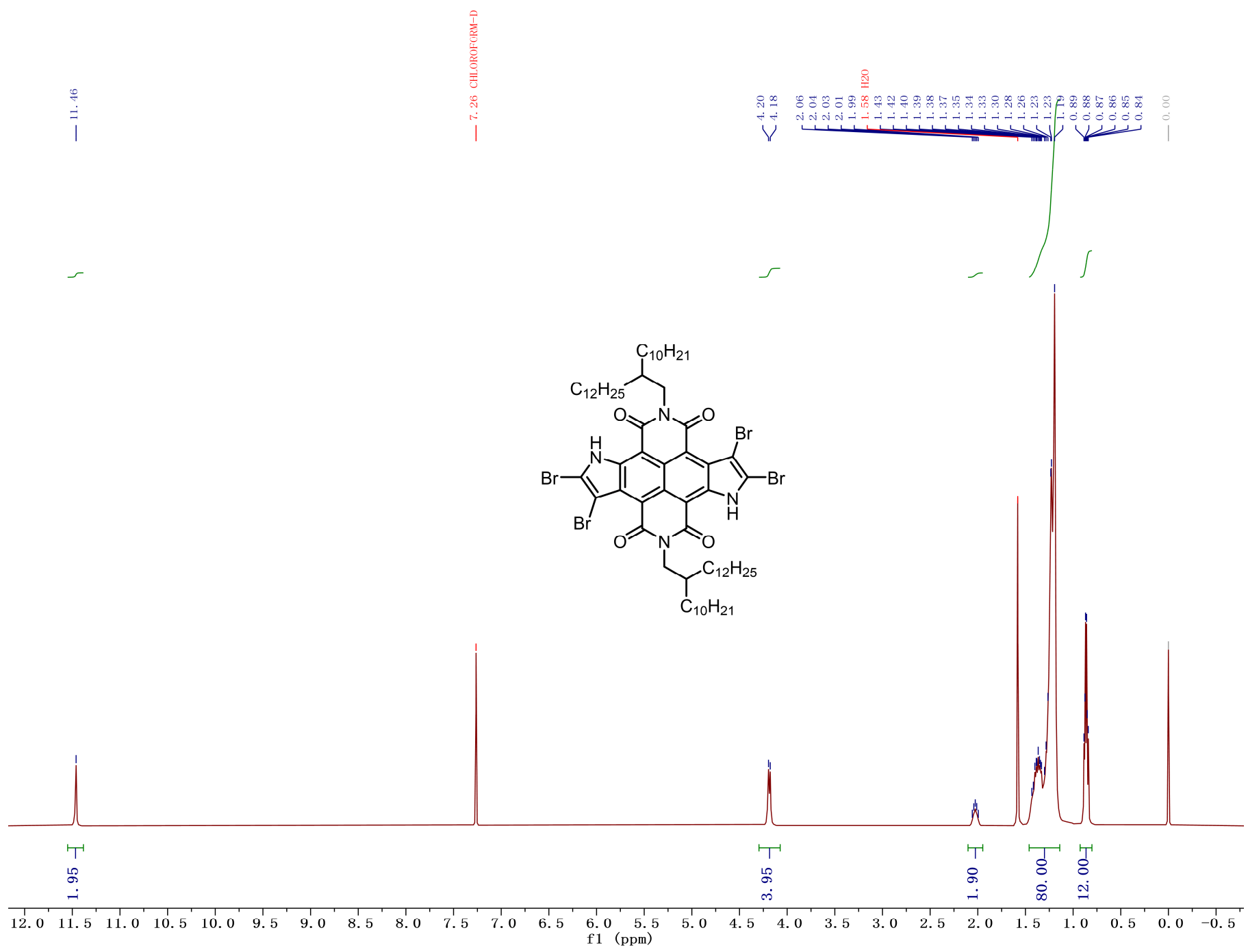

Figure S49. ^{1}H NMR spectrum (400 MHz) of **4Br-NDPI-DT** in $CDCl_3$.

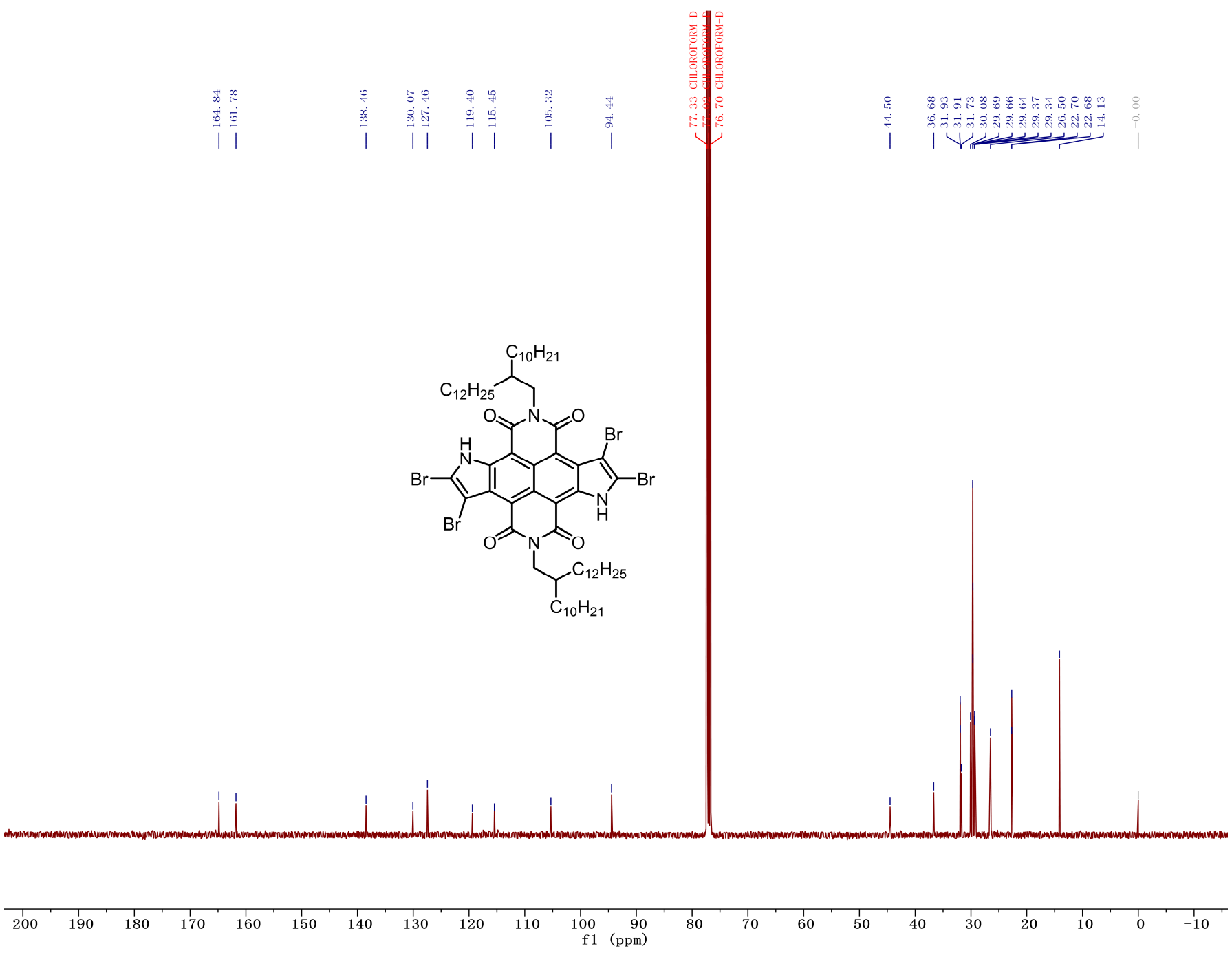

Figure S50. $^{13}C\{^{1}H\}$ NMR spectrum (100 MHz) of **4Br-NDPI-DT** in $CDCl_3$.

Reference:

1. Fukutomi, Y.; Nakano, M.; Hu, J.-Y.; Osaka, I.; Takimiya, K., Naphthodithiophenediimide (NDTI): Synthesis, Structure, and Applications. *J. Am. Chem. Soc.* **2013,** *135* (31), 11445-11448.
2. Frisch, M. J.; Trucks, G. W.; Schlegel, H. B.; Scuseria, G. E.; Robb, M. A.; Cheeseman, J. R.; Scalmani, G.; Barone, V.; Petersson, G. A.; Nakatsuji, H.; Li, X.; Caricato, M.; Marenich, A. V.; Bloino, J.; Janesko, B. G.; Gomperts, R.; Mennucci, B.; Hratchian, H. P.; Ortiz, J. V.; Izmaylov, A. F.; Sonnenberg, J. L.; Williams; Ding, F.; Lipparini, F.; Egidi, F.; Goings, J.; Peng, B.; Petrone, A.; Henderson, T.; Ranasinghe, D.; Zakrzewski, V. G.; Gao, J.; Rega, N.; Zheng, G.; Liang, W.; Hada, M.; Ehara, M.; Toyota, K.; Fukuda, R.; Hasegawa, J.; Ishida, M.; Nakajima, T.; Honda, Y.; Kitao, O.; Nakai, H.; Vreven, T.; Throssell, K.; Montgomery Jr., J. A.; Peralta, J. E.; Ogliaro, F.; Bearpark, M. J.; Heyd, J. J.; Brothers, E. N.; Kudin, K. N.; Staroverov, V. N.; Keith, T. A.; Kobayashi, R.; Normand, J.; Raghavachari, K.; Rendell, A. P.; Burant, J. C.; Iyengar, S. S.; Tomasi, J.; Cossi, M.; Millam, J. M.; Klene, M.; Adamo, C.; Cammi, R.; Ochterski, J. W.; Martin, R. L.; Morokuma, K.; Farkas, O.; Foresman, J. B.; Fox, D. J. *Gaussian 16 Rev. C.01*, Wallingford, CT, 2016.
3. Wang, Z. py.Aroma: An Intuitive Graphical User Interface for Diverse Aromaticity Analyses *Chemistry* [Online], 2024, p. 1692-1703.
4. a) Valeev, E. F.; Coropceanu, V.; da Silva Filho, D. A.; Salman, S.; Brédas, J.-L., Effect of Electronic Polarization on Charge-Transport Parameters in Molecular Organic Semiconductors. *J. Am. Chem. Soc.* **2006,** *128* (30), 9882-9886; b) Ottonelli, M.; Piccardo, M.; Duce, D.; Thea, S.; Dellepiane, G., Koopmans' Transfer Integral Calculation: A Comparison between the Hartree-Fock and the Density Functional Results. *Energy Procedia* **2012,** *31*, 31-37.
5. Ito, Y.; Virkar, A. A.; Mannsfeld, S.; Oh, J. H.; Toney, M.; Locklin, J.; Bao, Z., Crystalline Ultrasmooth Self-Assembled Monolayers of Alkylsilanes for Organic Field-Effect Transistors. *J. Am. Chem. Soc.* **2009,** *131* (26), 9396-9404.
6. Xu, X.; Yao, Y.; Shan, B.; Gu, X.; Liu, D.; Liu, J.; Xu, J.; Zhao, N.; Hu, W.; Miao, Q., Electron Mobility Exceeding 10 cm2 V−1 s−1 and Band-Like Charge Transport in Solution-Processed n-Channel Organic Thin-Film Transistors. *Adv. Mater.* **2016,** *28* (26), 5276-5283.
7. a) Gao, X.; Di, C.-a.; Hu, Y.; Yang, X.; Fan, H.; Zhang, F.; Liu, Y.; Li, H.; Zhu, D., Core-Expanded Naphthalene Diimides Fused with 2-(1,3-Dithiol-2-Ylidene)Malonitrile Groups for High-Performance, Ambient-Stable, Solution-Processed n-Channel Organic Thin Film Transistors. *J. Am. Chem. Soc.* **2010,** *132* (11), 3697-3699; b) Suraru, S.-L.; Zschieschang, U.; Klauk, H.; Würthner, F., A core-extended naphthalene diimide as a p-channel semiconductor. *Chem. Commun.* **2011,** *47* (41), 11504-11506; c) Chen, X.; Guo, Y.; Tan, L.; Yang, G.; Li, Y.; Zhang, G.; Liu, Z.; Xu, W.; Zhang, D., Dithiazole-fused naphthalene diimides toward new n-type semiconductors. *J. Mater. Chem. C* **2013,** *1* (6), 1087-1092; d) Hu, Y.; Gao, X.; Di, C.-a.; Yang, X.; Zhang, F.; Liu, Y.; Li, H.; Zhu, D., Core-Expanded Naphthalene Diimides Fused with Sulfur Heterocycles and End-Capped with Electron-Withdrawing Groups for Air-Stable Solution-Processed n-Channel Organic Thin Film Transistors. *Chem. Mater.* **2011,** *23* (5), 1204-1215.
